# Coumarin derivatives from *Ainsliaea fragrans* and their anticoagulant activity

**DOI:** 10.1038/srep13544

**Published:** 2015-08-28

**Authors:** Liang Lei, Yong-bo Xue, Zhong Liu, Si-si Peng, Yan He, Yang Zhang, Rong Fang, Jian-ping Wang, Zeng-wei Luo, Guang-min Yao, Jin-wen Zhang, Geng Zhang, Hong-ping Song, Yong-hui Zhang

**Affiliations:** 1 Puai Hospital Affiliated to Tongji Medical College, Huazhong University of Science and Technology, Wuhan 430030, People’s Republic of China; 2 Hubei Key Laboratory of Natural Medicinal Chemistry and Resource Evaluation, School of Pharmacy, Tongji Medical College, Huazhong University of Science and Technology, Wuhan 430030, People’s Republic of China; 3 Tongji Hospital Affiliated to Tongji Medical College, Huazhong University of Science and Technology, Wuhan 430030, People’s Republic of China; 4 Department of Pharmacy, Wuhan First Hospital, Wuhan 430022, Hubei, People’s Republic of China

## Abstract

Coumarin derivatives are an important class of C_6_–C_3_ plant metabolites that show a variety of bioactivities. Currently, most clinical anticoagulant agents are coumarins, such as warfarin, dicoumarol and acenocoumarol, and patients taking these drugs must be monitored for adverse reactions. In a search for safe and effective anticoagulant compounds from Chinese herbal medicine, a screening procedure on the whole plant of *Ainsliaea fragrans* was performed. The phytochemical investigation of this plant afforded five new coumarin derivatives, including a pair of natural 4-hydroxycoumarin enantiomers (**1**), a pair of coumarin enantiomers with a rare polycyclic pyrano[3-2c] carbon skeleton (**2**) and a 7-hydroxycoumarin derivative (**3**), together with 5 known biogenetically related compounds (**4**–**8**). Enantioseparation of **1** and **2** produced optically pure compounds **1a**, **1b**, **2a** and **2b**. The absolute configurations of the new compounds were confirmed by single-crystal X-ray diffraction analysis. In addition, we evaluated the anticoagulant activity of all isolates via activated partial thromboplastin time (APTT), thrombin time (TT) and prothrombin time (PT) assays *in vitro* and *in vivo*. Of note, compound **3** displayed potent anticoagulant activity and no significant hepatic or renal toxicity, which could make it a promising agent for further preclinical evaluation for preventing abnormal blood clotting.

Coumarins are a well-known class of secondary metabolites in plants^1^,^2^,^3^ and fungi^4^,^5^,^6^ Owing to their structural features, coumarins are an important type of substrate in the areas of natural product modification and synthetic chemistry^7^,^8^,^9^. Among the various coumarin derivatives, 4-hydroxycoumarins, which have a special enol moiety, have shown particularly high activity in chemical synthesis and can act as potent metal ligands and starting material^10^,^11^. The C3-substituted 4-hydroxycoumarins in particular have attractive biological activities^12^, especially anticoagulant activity.

Cardio-cerebrovascular disease caused by thromboembolism poses a serious threat to human health. Coumarins are widely used in the clinic for antithrombotic therapy; for example, warfarin, which used to be a rodenticide, is now used as an anticoagulant^13^,^14^. However, the therapeutic use of coumarin agents is severely limited by their associated adverse reactions, such as platelet disease and haemorrhages.

In order to search for novel, highly efficient anticoagulant compounds with low toxicity from Chinese herbal medicine, a study was conducted on extracts of *Ainsliaea fragrans* Champ (Compositae).

*Ainsliaea fragrans*, also known as “xing-xiang-tu-er-feng”, is mainly distributed in the south of China^15^ and is still applied as a prescription medicine for treating chronic cervicitis. A few compounds have been reported to be found in this plant, including seven sesquiterpenoids^16^,^17^,^18^, six sesquiterpene glycosides^19^ and two phenolic compounds^20^,^21^. Herein, we report five new coumarin derivatives and five other known, biogenetically related coumarin derivatives isolated from this plant. Two pairs of new natural C3-substituted 4-hydroxycoumarin enantiomers **1** and **2** were enantioseparated successfully. A preliminary assay was carried out to evaluate the anticoagulant activity of all of the isolates.

## Results and Discussion

This study is focused on identifying the structure of coumarin derivatives from *Ainsliaea fragrans* and investigating the anticoagulant activity of these isolates *in vitro* and *in vivo*.

### Structural elucidation

**Figure 1:**
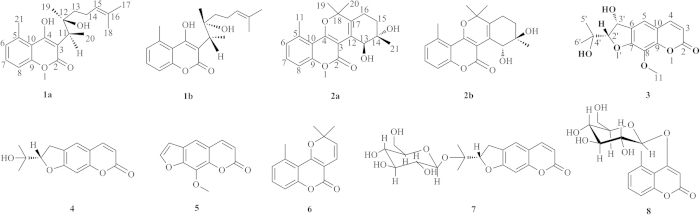
Isolated compounds from extracts of *Ainsliaea fragrans*.

Compound **1** was obtained as colourless needles. Its molecular formula, C_20_H_26_O_4,_ was determined by HRESIMS at *m/z* 331.1894 [M + H^+^] [calcd. for C_20_H_27_O_4_^+^ *m/z* 331.1904], indicating eight degrees of unsaturation. The IR spectrum of **1** showed absorption bands assignable to a benzene group and a conjugated ester group (1601 and 1645 cm^−1^). The ^1^H NMR spectrum showed three aromatic olefinic protons at *δ*_H_ 7.01 (br d, *J* = 7.4 Hz, H-6), 7.34 (dd, *J* = 7.4, 8.0 Hz, H-7) and 7.24 (br d, *J* = 8.0 Hz, H-8); five methyl signals at *δ*_H_ 1.58 (s, H_3_-17), 1.67 (s, H_3_-18), 2.78(s, H_3_-21), 1.54 (d, *J* = 7.3 Hz, H_3_-20) and 1.50 (s, H_3_-19); and two methylene signals at *δ*_H_ 1.98 (m, H-13) and 2.26 (m, H-14). (Table 1) The ^13^C NMR and DEPT spectra revealed the presence of 20 carbon resonances, including one conjugated ester, ten olefinic carbons, five methyls, and two methylenes. The presence of a 1, 2, 3-trisubstituted phenyl moiety was supported by the NMR information as follows: *δ*_H_ 7.01 (H-6) to *δ*_C_ 128.8 (C-6), *δ*_H_ 7.34 (H-7) to *δ*_C_ 132.2 (C-7), and *δ*_H_ 7.24 (H-8) to *δ*_C_ 116.1 (C-8). A characteristic single peak (*δ*_H_ 2.78, *δ*_C_ 24.9) suggested an aromatic methyl. The 1D NMR data of **1** were similar to those of cyclobrachycoumarin^22^,^23^,^24^, which indicated that **1** was a 5-methylcoumarin derivative. Of the 20 carbon resonances, ten were typical of a 5-methylcoumarin moiety, whereas the additional ten carbons consisted of two isoprene moieties, as shown by HMBC correlations from H-11 to C-12/C-20/C-19, from H_3_-20 to C-11/C-3/C-12, from H_3_-19 to C-11/C-12/C-13, and from H_3_-18/H_3_-17 to C-16/C-15. The two isoprene moieties were connected by C-13 and C-14, as shown by the ^1^H-^1^H COSY spectrum vicinal couplings between H-14 (*δ* 2.26) and H-13 (*δ* 1.98). Furthermore, the 5-methylcoumarin moiety and the isoprene side-chain were connected at C-3, as shown by the HMBC correlations from H-11 to C-3/C-4 and from H_3_-20 to C-3/C-11. All of these signals suggested that **1** was a C-3 substituted 5-methylcoumarin. (Fig. 1)

**Figure 2:**
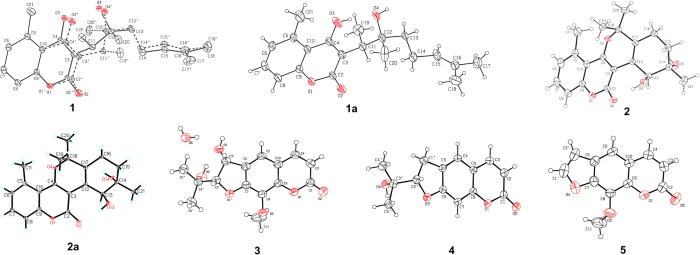
ORTEP drawings of compounds 1, 1a, 2, 2a, 3, 4, and 5.

The relative configuration of the two ortho-position chiral carbon atoms (C-11 and C-12) could not be unambiguously determined by NOE correlations. After many attempts, a suitable crystal of **1** was obtained from a solvent system of CH_2_Cl_2_/MeOH/H_2_O (Fig. 2). However, the single-crystal X-ray diffraction experiment showed that compound **1** was a mixture of two enantiomers in a ratio of 62.7%:37.3%. Therefore, the planar structure of **1** was constructed as a natural C3-substituted 4-hydroxy coumarin.

**Figure 3:**
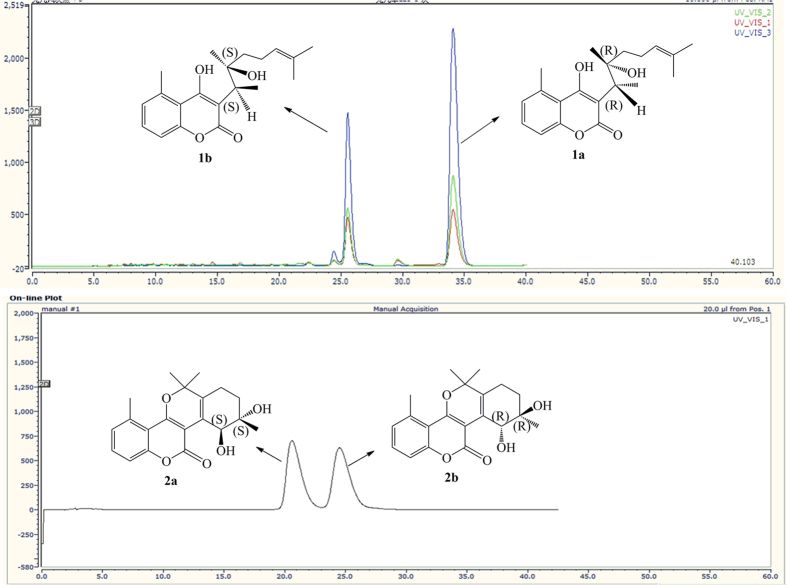
Enantioseparation of compounds 1 and 2 by chiral columns.

**Figure 4:**
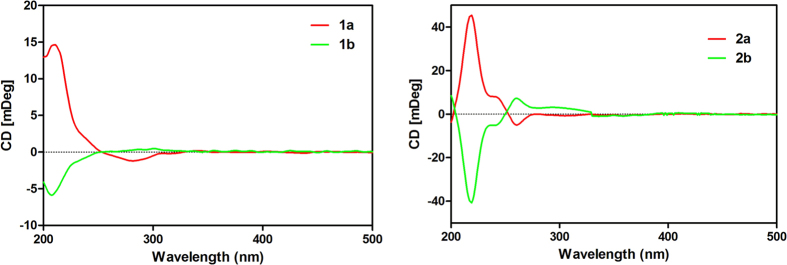
CD spectra of compounds 1a, 1b, 2a and 2b.

Chiral analysis and optical resolution of **1** were achieved by HPLC with a CHIRALPAK AS-H chiral column (*n*-hexane/*i*-PrOH, 95:5) at 0.8 ml/min, which afforded compounds **1a** and **1b**. The relative peak area ratio of **1a** to **1b** was approximately 2:1, consistent with the X-ray result (62.7%:37.3%). (Fig. 3) Similarly, a suitable crystal of **1a** for the single-crystal X-ray diffraction experiment was obtained. Remarkably, the melting point of **1** was at 184 °C, but the melting points of **1a** and **1b** were increased to 191 °C and 190 °C, respectively. Finally, the absolute configuration of **1a** was confirmed to be 11*R*, 12*R*, and the absolute configuration of **1b** was confirmed as 11*S*, 12*S*, by combining the X-ray result of **1** and the CD spectrum of **1a** (Fig. 4). Thus, the structures of **1a** and **1b** were assigned and named ainsliaeasin A1 and ainsliaeasin A2.

Compound **2** was obtained as colourless needles. Its formula C_20_H_22_O_5_ was determined by HRESIMS at *m/z* 343.1531 [M + H^+^] [calcd. for C_20_H_23_O_5_^+^ *m/z* 331.1540]. The IR bands at 1671.2 and 1605.9 suggested a benzene group and a conjugated ester group. Its ^1^H NMR spectrum (Table 1) showed three phenyl protons at *δ*_H_ 7.02 (br d, *J* = 7.5 Hz, H-6), 7.34 (dd, *J* = 7.5, 8.0 Hz, H-7), and 7.15 (br d, *J* = 8.0 Hz, H-8), which indicated a 1, 2, 3-substituted phenyl moiety. A characteristic methyl singlet (*δ*_H_ 2.69, *δ*_C_ 23.1) implied that **2** was also a 5-methylcoumarin. The ^13^C NMR of **2** showed 20 carbon resonances comprising a conjugated ester carbonyl, ten sp^2^ carbons, two quaternary sp^3^, two sp^3^ methine, one sp^3^ methylene, and four methyls. The ^1^H and ^13^C NMR spectra of **2** were extremely similar to those of gerberlin B^25^, as further supported by the 2D NMR spectroscopic spectra, including ^1^H-^1^H COSY, HSQC, and HMBC spectra. However, the specific rotatory and CD spectra of **2** could not be detected. To confirm the planar structure of **2**, a single-crystal X-ray diffraction experiment was performed, which indicated that the space group of **2** was a mixture of two enantiomers.

Like **1**, **2** was resolved to **2a** and **2b** using a chiral column (CHIRALPAK AD) under reverse phase conditions (Acetonitrile/H_2_O = 40/60) at 0.5 ml/min. The relative retention times for **2a** and **2b** were 20.8 min and 24.9 min, respectively. The relative peak areas of **2a** and **2b** were approximately 1:1, which was highly consistent with the X-ray result (ee 50%). In addition, the melting point of **2** was 208 °C, but the melting points of **2a** and **2b** increased to 214 °C and 213 °C, respectively. The successful X-ray diffraction with Cu-K*α*, which resulted in a Flack parameter of 0.11(15), allowed an unambiguous assignment of the absolute configuration of **2a** as 12*S*, 13*S*. Based on the X-ray of **2** and CD spectrum of **2a**, the absolute configuration of compound **2b** was confirmed as 12*R*, 13*R*. Finally, the structures of **2a** and **2b** were assigned and named ainsliaeasin B1 and ainsliaeasin B2.

Compound **3** was obtained as colourless needles. Its molecular formula C_15_H_16_O_6_ was determined by HRESIMS at *m/z* 293.1013 [M + H^+^] [calcd. for C_15_H_17_O_6_^+^ *m/z* 293.1020]. The UV spectrum exhibited *λ*_max_ at 323 nm and 211 nm, suggesting the presence of a benzene conjugated system and an unsaturated ester moiety, as shown by IR bands at 1695.3, 1609.3, and 1578.1. The NMR spectrum of **3** was similar to that of **4** nodakenetin^26^, except for the presence of one more methoxy group and one more hydroxy group. This observation was supported by the HMBC data, which showed correlations of H_3_-11 (*δ*_H_ 4.06, s) to C-8 (*δ*_C_ 132.6) and H-2′ (*δ*_H_ 4.41, d) to C-3′ (*δ*_C_ 72.5). The hydroxy group was also observed in the ^1^H-^1^H COSY spectrum from H-2′ to H-3′ (*δ*_H_ 5.37, d). The absolute configuration of **3** was determined as 2′*S* and 3′*R* by X-ray diffraction with a Flack parameter of 0.09(12). As a result, the structure of **3** was assigned and named ainsliaeasin C.

To date, only two polycyclic pyrano [3-2c] coumarins (gerberlin A and gerberlin B) have been isolated from *Gerbera saxatilis*^25^, so **2** is the third example of this carbon skeleton. In nature, chiral natural products are usually produced in optically pure forms; however, occasionally, both enantiomers are formed^27^. In this study, the enantiomers of **1** and **2** were isolated then enantioseparated to obtain compounds **1a**, **1b**, **2a**, and **2b**.

Other compounds found included xanthotoxin (**5)**^28^, bothrioclinin (**6**)^29^, nodakenin (**7**)^30^, and gerberinside (**8**)^31^.

### *In vitro* coagulation studies

All of the compounds isolated from this plant were analysed for their anticoagulant activities by monitoring the activated partial thromboplastin time (APTT), thrombin time (TT) and prothrombin time (PT). Coumarins usually interfere with the intrinsic coagulation process by inhibiting the vitamin K conversion cycle, but not with the extrinsic process^32^. Consistent with this finding, compound **4**, without 3′-hydroxyl, and compound **5**, without 2′-isopropyl, presented no anticoagulation effects. However, compound **3**, with a hydroxyl group at the 3′-position, showed moderate anticoagulant activity *in vitro* by significantly increasing the PT, exceeding the full scale of the instrument. The other compounds were inactive according to these three parameters. (Table 2)

By comparing the structures of compounds **3**, **4** and **5**, it was concluded that the anticoagulant activity is closely related to the 3′-hydroxy and 2′-isopropyl moieties. Subsequently, compound **3** was found to exert anticoagulant activity at a minimum concentration of 1 mg/ml. (Table 2)

### *In vivo* coagulation studies

To estimate the putative *in vivo* efficacy, we performed studies in Wistar rats to measure anticoagulant activity. Initially, the dose and sampling time of warfarin were tested on rats at 1 mg/kg, 0.5 mg/kg, and 0.2 mg/kg after 1 day, 2 days, and 3 days.

To evaluate the *in vivo* anticoagulant activity of compounds **1**–**8**, the APTT, PT, and TT were determined on the third and fifth days after administration. After treating for 3 days, compound **7** markedly prolonged the TT (P < 0.01), whereas compound **8** extended it to an insignificant degree (P > 0.05). However, compounds **1**, **2**, **3**, **4**, **5**, and **6** did not change the APTT, PT or TT on the third day compared with the negative control group. (Table 3)

To better characterize the anticoagulant profile, changes in the APTT, PT, and TT values were also determined on the fifth day. The PT and TT were prolonged in rats treated with compounds **3** and **4**, whereas compounds **5** and **6** increased the TT value but not to a significant degree (P > 0.05). The TT values of group **7** and group **8** reached normal levels on the fifth day. (Table 3) The result showed that compound **3** exhibited anticoagulant activity at 1 mg/kg in Wistar rats.

Moreover, no death was found in rats treated with compound **3**. However, one death caused by haemorrhage was observed in each of the groups treated with warfarin, compound **1** and compound **7** on the third day, and another death was observed in each of the groups treated with warfarin, compound **1**, compound **4** and compound **8** on the fifth day. As a result, compound **3** was shown to be less toxic than the other compounds.

After the dissection of the rats on the seventh day, no significant liver or renal toxicity was observed in any of the groups of rats.

## Conclusion

In this study, five new coumarins were isolated from *Ainsliaea fragrans* Champ. The chiral resolution of enantiomers (**1** and **2**) led to optically pure compounds **1a**, **1b**, **2a** and **2b**. Their planar structures and absolute configurations were determined by NMR, X-ray and CD analysis. Biologically, the anticoagulant activity of all of the isolates was evaluated. Compound **3** had anticoagulant activity both *in vitro* and *in vivo*. Additionally, compound **3** proved to be less toxic than warfarin and showed no significant liver or kidney toxicity. However, it is important to note that the results of the current study are preliminary, pending confirmation of the anticoagulant activity *in vitro* and *in vivo.* Further research is necessary to evaluate the action and mechanism of action of compound **3**.

## Methods

### General

The melting point (uncorrected) was determined on an apparatus made by Beijing TECH INSTRUMENT CO. LTD. Optical rotations were measured with a Perkin Elmer spectropolarimeter. The UV spectra were measured on a VARIAN SARY 50 spectrophotometer. The IR spectra were recorded using a BRUKER VERTEX 70 spectrometer. The NMR experiments were run on a Bruker AM-400 spectrometer. The HRFABMS data were obtained on a VG 7070-HF spectrometer. Column chromatographic separations were carried out using silica gel H60 and ODS as packing materials. HSGF254 silica gel TLC plates were used for analytical TLC. The HPLC columns consisted of a Welch Material column (XB-C18, 10 *μ*m, 10 × 250 mm), a normal phase chiral column (CHIRALPAK AS-H, 10 *μ*m, 4.6 mm × 250 mm, part no. 20325), and a reversed phase chiral column (CHIRALPAK AD-RH, 10 *μ*m, 4.6 mm × 150 mm, part no. 19724). The automatic coagulative instrument (Sysmex CA-7000), activated partial Thromborel S, Thrombin and Dade Actin Activated Cephaloplastin Reagent were commercial reagents from Siemens Healthcare Diagnostics Products GmbH. The semi-automatic biochemical analyser (MC-4000, Germany).

### Plant material

The *Ainsliaea fragrans* Champ whole plants were collected from Shiyan City, Hubei Province, P. R. C., in 2013 and identified by Dr. Jian-Ping Wang, Tongji Medical Collage, Huazhong University of Science and Technology. A voucher specimen (No. 20130701) was deposited at Hubei Key Laboratory of Natural Medicinal Chemistry and Resource Evaluation, School of Pharmacy, Tongji Medical College, Huazhong University of Science and Technology.

### Extraction and isolation

The whole plant of *Ainsliaea fragrans* (20 kg) was percolated with 95% industrial ethanol at room temperature. The filtrate was concentrated in vacuo. The residue was partitioned with petroleum ether (200 g), EtOAc (120 g), and *n*-BuOH (150 g), successively.

The petroleum ether portion was subjected to column chromatography over silica gel eluted with a petroleum-acetone gradient to afford five fractions (A–E). Fraction B was separated into three subfractions, B1, B2, and B3. Compound **1** (15.0 mg, t_*R*_ 46.2 min) was separated from B2 by semipreparative HPLC (80:20 MeOH–H_2_O, 230 nm, 2.0 ml/min), while **1a** and **1b** were obtained from a chiral column (CHIRALPAK AS-H) under normal phase conditions (n-hexane : isopropanol = 9:1) at 0.5 ml/min using a UV detector at 230 nm, 254 nm and 210 nm; the relative retention times of **1a** and **1b** were 34.3 min and 25.5 min, respectively. Compound **5** (50 g) was recrystallized from fraction C, while compound **6** (15 g) was recrystallized from fraction D.

The EtOAc portion was subjected to column chromatography over silica gel eluted with a gradient system of CH_3_Cl–MeOH (100:1–1:100) to give five fractions F–J. Fraction F was separated into three subfractions, F1A, F1B and F1C, by Sephadex LH-20 using MeOH. Fraction F1B was also separated into two subfractions, F1BA and F1BB, by silica gel using a system of MeOH–CHCl_3_ (40:1–30:1). Compound **7** (4.0 mg) was separated from fraction F1BA on silica gel using a system of MeOH–CHCl_3_ (30:1–20:1). Similarly, compounds **3** (11.3 mg) and **4** (25.6 mg) were separated from fraction G. Compound **2** (19 mg) was separated from fraction H on silica gel using a system of MeOH–CHCl_3_ (20:1–10:1), and compounds **2a** (1.2 mg) and **2b** (1.1 mg) were separated on a chiral column (CHIRALPAK AD) under reverse phase conditions (acetonitrile : H_2_O = 40 : 60) at 0.5 ml/min using a UV detector at 230 nm; the relative retention times were 20.8 min and 24.9 min, respectively.

The *n*-BuOH portion was separated using a silica gel column eluted with a CH_3_Cl-MeOH gradient to afford four fractions to obtain **7** (22 mg) and **8** (180 mg).

### Spectroscopic data of the isolated compounds

Compound **1**: colourless needles; m.p. 184.0–185.0 °C; 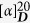 + 1.0 (*c*, 0.6, MeOH); UV (MeOH) νmax (log ε) 208 (3.52) nm, 296 (4.52) nm; IR(KBr) νmax 3222.1, 2923, 1644.8, 1600.9, 1563.7, 1332.2, 784.5, 744.8; ^1^H (Py-*d*_5_, 400 MHz) and ^13^C(Py-*d*_5_, 100 MHz) NMR data, see Table 1; HRESIMS m/z 331.1849 [M + H^+^] [calcd. for C_20_H_27_O_4_^+^ m/z 331.1904].

Compound **1a**: colourless needles; m.p. 191.0–192.0 °C; 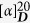 + 18.0 (c, 0. 3, MeOH); CD (CH_3_OH, 3.0 mM) λ_extr._ (θ [mdeg cm^2^ dmol^−1^]) 282 (−1.2), 210 (+14.6).

Compound **1b**: colourless tiny needles; m.p. 190.0–191.0 °C; 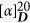 − 12.0 (c, 0.3, MeOH); CD (CH_3_OH, 1.0 mM) λ_extr._ (*θ* [mdeg cm^2^ dmol^−1^]) 301 (+0.5), 208 (−5.9).

Compound **2**: colourless needles; m.p. 208.0 °C; 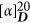 0 (*c*, 0.8, MeOH); UV (MeOH) νmax (log ε) 214 (3.46) nm, 265 (3.44) nm, 342 (4.40) nm; IR(KBr) νmax 3425.6, 2921.1, 1671.1, 1605.8, 1584.9, 1368.7, 1009.0, 786.7, 740.3; ^1^H (CDCl_3_, 400 MHz) and ^13^C(CDCl_3_, 100 MHz) NMR data, see Table 1; HRESIMS m/z 343.1531 [M + H^+^] [calcd. for C_20_H_23_O_5_^+^ m/z 331.1540].

Compound **2a**: colourless needles; m.p. 214.0 °C; 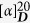 + 22 (*c*, 0.5, MeOH); CD (CH_3_OH, 3.0 mM) λ_extr_. (*θ* [mdeg cm^2^ dmol^−1^]) 261 (−4.8), 240 (+8.5), 218 (+45.1).

Compound **2b**: colourless needles; m.p. 213.0 °C; 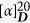 − 20 (*c*, 0.5, MeOH); CD (CH_3_OH, 3.0 mM) λ_extr_. (*θ* [mdeg cm^2^ dmol^−1^]) 256 (+7.8), 240 (−4.5), 218 (−39.5).

Compound **3**: colourless needles; m.p. 106.0–107.0 °C; 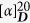 − 4.0 (*c*, 0.3, MeOH); UV (MeOH) νmax (log ε) 211 (3.77) nm, 323 (4.70) nm; CD (CH_3_OH, 3.0 mM) λ_extr._ (*θ* [mdeg cm^2^ dmol^−1^]) 301 (+7.5), 256 (−3.8), 228 (−19.5), 207 (+32.8); IR(KBr) νmax 3425.6, 2921.9, 1695.3, 1609.3, 1576.1, 1407.9, 1151.1, 775.5; ^1^H(CD_3_OD, 400 MHz) and ^13^C(CD_3_OD, 100 MHz) NMR data, see Table 1. HRESIMS m/z 293.1013 [M + H^+^] [calcd. for C_20_H_23_O_5_^+^ m/z 293.1020].

### Single-Crystal X-ray Diffraction Analysis and Crystallographic Data of Compounds 1, 1a, 2, 2a, 3, 4, and 5.

Diffraction intensity data for compounds **1**, **1a**, **2**, **2a**, **3**, **4**, and **5** were acquired on a Bruker APEX-II diffractometer employing graphite-monochromatized Cu Kα radiation (λ = 1.54178 Å) at 298(2) K or Mo Kα radiation (λ = 0.71073 Å) at 298(2) K. The data were collected by Bruker APEX2 software and reduced with Bruker SAINT. Structure solution and refinement were performed with the SHELXTL program package. All of the non-hydrogen atoms were refined anisotropically. The hydrogen atom positions were geometrically idealized and allowed to ride on their parent atoms. The crystal structures of compounds **1**, **1a**, **2**, **2a**, **3**, **4**, and **5** were drawn by ORTEP 3 for Windows (version 2.02). All of the data can be obtained free of charge from the CCDC via http://www.ccdc.cam.ac.uk/Community/Requestastructure/Pages/DataRequest.aspx.

*Crystal data for* **1**: colourless needles, C_20_H_26_O_4_; *M*_W_ = 330.41; Cu K*α* (*λ* = 1.54178 Å); temperature = 296 (2) K; triclinic; space group P-1; a = 7.2247(2) Å, b = 7.6916(2) Å, c = 16.5807 (3) Å, *α* = 79.1020(10)°, *β* = 82.8350(10)°, *γ* = 89.8210 (10)°; *V* = 897.50(4) Å^3^, *Z* = 2, D_calcd_ = 1.223 mg/m^3^, crystal size 0.20 × 0.10 × 0.10 mm^3^, Final *R* indices: R_1_ = 0.0482, w_R2_ = 0.1293; reflections collected: 13646. (CCDC No. 1028802).

*Crystal data for* **1a**: colourless needles, C_20_H_26_O_4_; M_W_ = 330.41; Cu K*α* (*λ* = 1.54178 Å); Temperature = 296 (2) K; triclinic; space group P1; a = 7.2264(2) Å, b = 7.6852(2) Å, c = 16.5860(3) Å, *α* = 100.8970(10)°, *β* = 96.9830(10)°, *γ* = 90.1240(10)°; *V* = 897.51(4) Å^3^, *Z* = 2, D_calcd_ = 1.223 mg/m^3^, crystal size 0.20 × 0.10 × 0.10 mm^3^, Final *R* indices: R_1_ = 0.0681, w_R2_ = 0.1914; reflections collected: 19991; Flack parameter = 0.3 (3). (CCDC No. 981339).

*Crystal data for* **2**: colourless needles, C_20_H_22_O_5_; M_W_ = 342.38; Cu K*α* (*λ* = 1.54178 Å); temperature = 298 (2) K; monoclinic; space group P2(1)/c; a = 5.65620(10) Å, b = 13.7286 (2) Å, c = 21.7344(4) Å, *α* = 90°, *β* = 91.2250(10)°, *γ* = 90°; *V* = 1687.33 (5) Å^3^, *Z* = 4, D_calcd_ = 1.348 mg/m^3^, crystal size 0.30 × 0.30 × 0.20 mm^3^, Final R indices: R_1_ = 0.0352, w_R2_ = 0.0949; reflections collected: 36236. (CCDC No. 1028800).

*Crystal data for* **2a**: colourless needles, C_20_H_22_O_5_; *M*_W_ = 342.38; Cu K*α* (*λ* = 1.54178 Å); temperature = 100 (2) K; orthorhombic; space group P21212; a = 13.7242(3) Å, b = 22.2290(5) Å, c = 5.5733(10) Å, *α* = 90°, *β* = 90°, *γ* = 90°; *V* = 1700.28 (6) Å^3^, *Z* = 4, D_calcd_ = 1.337 mg/m^3^, crystal size 1.60 × 0.18 × 0.11 mm^3^, Final *R* indices: R_1_ = 0.0376, w_R2_ = 0.1143; reflections collected: 12603; Flack parameter = 0.11(15). (CCDC No. 1028801).

*Crystal data for* **3**: colourless needles, C_15_H_18_O_7_; M_W_ = 310.29; Cu K*α* (*λ* = 1.54178 Å); temperature = 196 (2) K; monoclinic; space group P2(1); a = 5.5649(10) Å, b = 12.7775(3) Å, c = 10.2937(2) Å, *α* = 90°, *β* = 93.9340(10)°, *γ* = 90°; *V* = 730.21 (3) Å^3^, *Z* = 2, D_calcd_ = 1.411 mg/m^3^, crystal size 0.12 × 0.12 × 0.10 mm^3^, Final *R* indices: R_1_ = 0.0265, w_R2_ = 0.0734; reflections collected: 22485; flack parameter = 0.09(12). (CCDC No. 981338).

Crystal data for **4** (CCDC No. 981340) and **5** (CCDC No. 981341), see supporting information.

### Anticoagulant activity assay *in vitro* 33

Assays were performed for each sample using an automatic coagulative instrument (Sysmex CA-7000 System) according to the instructions provided by the biological reagent provider (Siemens Healthcare Diagnostics Products Gmbh).

Fresh whole blood (50 ml), collected in sodium citrate coagulation test tubes, was donated by the first author of this paper, which was approved by the ethics committee of Puai Hospital. After centrifugation (3000 rpm, 8 min), the supernatants (270 *μ*L) were divided into containers. Compounds **1**–**8** were diluted for use with normal saline (with 1% DMSO) at 1 mg/ml. Then, all of the isolates (30 *μ*L) were added into the plasma sequentially. After incubation in a 37 °C thermostatic water bath for 10 minutes, all of the samples were analysed with the automatic coagulative instrument, which had been supplied with activated partial Thromborel S, Throbin, Dade Actin Activated Cephaloplastin Reagent (Siemens Healthcare Diagnostics Products Gmbh) and calcium-chloride solutions. All of the tests were performed in an automated environment.

### Anticoagulant activity assay *in vivo* 34

The methods were carried out in Wistar rats accordance the European Community guidelines for the use of experimental animals and all experimental protocols were approved by the ethics committee of Puai Hospital. Rats were kept in polyethylene cages with wood shavings as bedding and maintained in a temperature controlled room at 20 ± 1 °C with a 12/12 h lighting schedule (lights on at 08:00 h, off at 20:00 h) and a relative humidity of 50% for at least 2 weeks prior to use.

All of the experiments were performed using adult male Wistar rats (250–300 g, body wt, Institute of Laboratory Animals of Sichuan Academy of Medical Sciences, SCXK 2013-24.) The animals were grouped and housed with seven per cage/group. The warfarin (0.2 mg/kg) (WUHAN XIANGHESHUNDA FINE CHEMICAL CO. LTD, XH20150206) and the tested compounds (1 mg/kg) were dissolved in sodium carboxymethyl cellulose and administered to animals by gavage for three days. Citrated blood was collected from the eye socket on the third day and the fifth day.

Platelet-poor plasma was prepared by centrifugation for measuring the APTT, PT, and TT on a semi-automatic biochemical analyser (MC-4000, Germany). All of the data are expressed in relative fold values, compared with the values obtained with the control group. The data were tested for statistical significance by nonparametric two-tailed Mann-Whitney test using the SPSS 17.0 software. A value of P < 0.05 was considered significant.

## Additional Information

**How to cite this article**: Lei, L. *et al.* Coumarin derivatives from *Ainsliaea fragrans* and their anticoagulant activity. *Sci. Rep.* **5**, 13544; doi: 10.1038/srep13544 (2015).

## Supplementary Material

Supporting Information

## Figures and Tables

**Table 1:** ^1^H and ^13^C NMR Data of Compounds 1, 2 and 3 a.

1^b^			2^c^			3^c^		
NO	*δ*_H_	*δ*_C_	NO	*δ*_H_	*δ*_C_	NO	*δ*_H_	*δ*_C_
1			1			1		
2		166.6	2		163.2	2		162.9
3		108.8	3		103.5	3	6.23 (d, 9.5)	113.0
4		165.5	4		161.9	4	7.85 (d, 9.5)	146.4
5		139.1	5		137.2	5	7.29 (s)	119.9
6	7.01 (d, 7.4)	128.8	6	7.02 (d, 7.5)	127.9	6		130.7
7	7.34 (dd, 7.4, 8.0)	132.2	7	7.34 (dd, 7.5, 8.0)	131.7	7		155.1
8	7.24 (d, 8.0)	116.1	8	7.15 (d, 8.0)	115.2	8		132.6
9		155.6	9		154.1	9		149.2
10		117.9	10		114.5	10		115.6
11	3.99 (q, 7.3)	41.2	11	2.69 (s)	23.6	11	4.06 (s)	61.3
12		77.3	12		123.3	1′		
13	1.97 (dd, 1.8, 6.8) 1.99 (dd, 1.8, 6.6)	42.2	13	4.16 (d, 3.8)	70.7	2′	4.41 (d, 6.0)	93.3
14	2.26 (m, overlap) 2.26 (m, overlap)	25.2	14		70.9	3′	5.37 (d, 6.0)	72.5
15	5.20 (ddd, 1.3, 7.0, 8.3)	125.8	15	1.72 (ddd, 1.0, 2.9, 6.6, 13.5) 1.94 (ddd, 6.2, 9.8, 13.5)	29.6	4′		73.1
16		133.0	16	2.07 (ddd, 2.9, 6.2, 18.0) 2.33 (ddd, 6.6, 9.8, 18.0)	22.3	5′	1.48 (s)	26.5
17	1.58 (s)	18.8	17		132.0	6′	1.51 (s)	27.1
18	1.67 (s)	27.0	18		82.1			
19	1.50 (s)	26.7	19	1.59 (s)	25.5			
20	1.54 (d, 7.3)	14.0	20	1.42 (s)	24.8			
21	2.78 (s)	24.9	21	2.69 (s)	23.6			

^a^Recorded at 400 and 100 MHz for ^1^H and^13^C. *J* values (Hz) are shown in parentheses.

^b^Spectra obtained in Pyridine-*d*_5_.

^c^Spectra obtained in CD_3_OD.

**Table 2:** PT, APTT, and TT of normal human platelet-poor plasma.

Reference/Fraction/Compounds	PT	APTT	TT
Heparin (3U)^a^	_ _ _._^**^^c^	_ _ _._^**^	_ _ _._^**^
Normal saline^b^	10.7 ± 0.02	25.8 ± 0.03	16.3 ± 0.04
1 (1 mg/ml)	10.6 ± 0.02	26.1 ± 0.04	18.1 ± 0.03
2 (1 mg/ml)	10.6 ± 0.00	25.7 ± 0.02	17.6 ± 0.04
3 (1 mg/ml)	_ _ _._^**^	25.8 ± 0.05	17.3 ± 0.03
4 (1 mg/ml)	10.8 ± 0.03	28.0 ± 0.06	17.8 ± 0.03
5 (1 mg/ml)	10.7 ± 0.00	26.1 ± 0.04	17.3 ± 0.05
6 (1 mg/ml)	10.6 ± 0.03	25.4 ± 0.02	17.1 ± 0.00
7 (1 mg/ml)	10.7 ± 0.03	26.3 ± 0.04	17.9 ± 0.01
8 (1 mg/ml)	11.0 ± 0.06	27.2 ± 0.07	17.7 ± 0.03
3 (2 mg/ml)	_ _ _._^**^	26.9 ± 0.07	18.4 ± 0.03
3 (1 mg/ml)	_ _ _._^**^	26.5 ± 0.05	18.4 ± 0.04
3 (0.5 mg/ml)	17.4 ± 0.04^*^	26.3 ± 0.03	17.7 ± 0.06
3 (0.25 mg/ml)	11.3 ± 0.01	25.8 ± 0.08	17.9 ± 0.01
3 (0.125 mg/ml)	11.2 ± 0.06	25.2 ± 0.05	17.3 ± 0.02

^a^Positive control

^b^negative control (1% DMSO)

^c^_ _ _._ exceeded the full scale of the instrument.

All of the data are expressed as mean ± SD (n = 3). ^*^P < 0.05,^**^P < 0.01, compared with the negative control group.

**Table 3:** Effects of compounds 1–8 on the APTT, PT, and TT clotting assays.

Groups	Dose (mg/kg)	3 days later after the last administration				5 days later after the last administration			
		*n*	APTT(*S*)	PT(*S*)	TT(*S*)	*n*	APTT(*S*)	PT(*S*)	TT(*S*)
CMC-Na	–	0	18.8 ± 0.7	29.1 ± 5.1	77.4 ± 10.6	0	19.1 ± 0.5	30.1 ± 4.1	78.4 ± 9.7
Warfarin	0.2	1	28.7 ± 2.3***	53.7 ± 7.3***	79.6 ± 15.8	1	29.5 ± 2.2***	55.7 ± 6.6***	80.6 ± 14.7
1	1.0	1	19.9 ± 1.1	28.7 ± 3.5	87.1 ± 25.8	1	19.6 ± 2.4	26.5 ± 5.1	63.8 ± 6.3
2	1.0	0	18.4 ± 1.1	28.8 ± 6.9	80.4 ± 21.3	0	18.7 ± 1.0	30.0 ± 4.2	73.1 ± 10.1
3	1.0	0	17.5 ± 1.3	26.7 ± 4.2	75.5 ± 18.4	0	19.4 ± 1.6	41.2 ± 4.7***	128.5 ± 30.6**
4	1.0	0	18.7 ± 1.2	30.1 ± 3.9	82.5 ± 12.7	1	18.9 ± 1.2	40.1 ± 8.0*	210.2 ± 67.2*
5	1.0	0	17.8 ± 0.7	29.3 ± 4.8	75.7 ± 11.6	0	18.4 ± 1.3	31.0 ± 6.1	87.0 ± 34.8
6	1.0	0	18.6 ± 1.1	28.0 ± 6.6	74.8 ± 25.0	0	18.3 ± 0.9	31.1 ± 3.8	143.5 ± 106.4
7	1.0	1	19.3 ± 0.6	31.8 ± 3.8	101.1 ± 12.9**	0	17.9 ± 0.3	27.9 ± 2.4	72.9 ± 3.6
8	1.0	0	18.8 ± 1.3	29.1 ± 4.5	108.8 ± 54.7	1	18.2 ± 0.7	28.8 ± 4.5	75.9 ± 9.7

*n* = the number of death of rats caused by haemorrhage.

All of the data are expressed as mean ± SD (n = 3). *P < 0.05, **P < 0.01, ***P < 0.001; compared with the negative control group.
