# Supplementary material for: Coumarin derivatives from Ainsliaea fragrans and their anticoagulant activity
Source: Sci Rep. 2015 Aug 28;5:13544. doi: 10.1038/srep13544 (PMC4551958; doi:10.1038/srep13544)

Supporting Information

Coumarin derivatives from *Ainsliaea fragrans* and their anticoagulant activity

Liang Lei[1 †], Yong-bo Xue[2 †], Zhong Liu[1], Si-si Peng[1], Yan He[2], Yang Zhang[2], Rong Fang[1], Jian-ping Wang[2], Zeng-wei Luo[2], Guang-minYao[2], Jin-wen Zhang[3], Geng Zhang[4], Hong-ping Song[1 *] & Yong-hui Zhang[2 *]

[1] *Puai Hospital Affiliated to Tongji Medical College, Huazhong University of Science and Technology, Wuhan 430030, People's Republic of China*

[2] *Hubei Key Laboratory of Natural Medicinal Chemistry and Resource Evaluation, School of Pharmacy, Tongji Medical College, Huazhong University of Science and Technology, Wuhan 430030, People's Republic of China*

[3] *Tongji Hospital Affiliated to Tongji Medical College, Huazhong University of Science and Technology, Wuhan 430030, People's Republic of China*

[4] *Department of Pharmacy, Wuhan First Hospital, Wuhan 430022, Hubei, People's Republic of China*

Corresponding authors.

Tel./fax: +86 027 83692311(Y.H. Zhang),+86 027-68835014 (H. P. Song);

Email: zhangyh@mails.tjmu.edu.cn (Y. H. Zhang), songhongping@126.com (H. P. Song)

† These authors contributed equally.

Content

Figure S1. HRESIMS of compound **1** ... 3
Figure S2. The UV spectrum of compound **1** ... 3
Figure S3. IR spectrum of Compound **1** ... 4
Figure S4. ^{1}H-NMR of Compound **1** in Pyridine-d_5, 400 MHz ... 4
Figure S5. ^{13}C-NMR and DEPT NMR of Compound **1** in Pyridine-d_5, 100 MHz ... 5
Figure S6. HSQC of Compound **1** in Pyridine-d_5 ... 5
Figure S7. HMBC of Compound **1** in Pyridine-d_5 ... 6
Figure S8. ^{1}H-^{1}H COSY of Compound **1** in Pyridine-d_5 ... 6
Figure S9. NOESY spectrum of Compound **1** in Pyridine-d_5 ... 7
Figure S11. ORTEP View of Compound **1**. ... 8
Figure S12 Enantioseparation of Compound **1** by HPLC. ... 8
Figure S13 The crystal packing of Compound **1a** ... 9
Figure S14 ORTEP View of Compound **1a.** ... 9
Figure S15 HRESIMS of compound **2** ... 10
Figure S16 The UV spectrum of compound **2** ... 10
Figure S17 The IR spectrum of compound **2** ... 11
Figure S18 ^{1}H-NMR of Compound **2** in CD_3Cl, 400 MHz ... 11
Figure S19^{13}C-NMR and DEPT of Compound **2** in CD_3Cl, 100 MHz ... 12
Figure S20 HSQC of Compound **2** in CD_3Cl. ... 12
Figure S21 HMBC of Compound **2** in CD_3Cl. ... 13
Figure S22 ^{1}H-^{1}H COSY of Compound **2** in CD_3Cl. ... 13
Figure S23 NOE of Compound **2** in CD_3Cl. ... 14
Figure S24 The crystal packing of Compound **2** ... 14
Figure S25 Enantioseparation of Compound **2** by HPLC. ... 15
Figure S26 The crystal packing of Compound **2a** ... 15
Figure S27 ORTEP View of Compound **2a** ... 15
Figure S28 HRESIMS of compound **3** ... 16
Figure S29 IR spectrum of Compound **3** ... 16
Figure S30UV spectrum of Compound **3** ... 17
Figure S31 ^{1}H-NMR of Compound **3** in CD_3OD, 400 MHz ... 17
Figure S32 ^{13}C-NMR and DEPT of Compound **3** in CD_3OD, 100 MHz ... 18
Figure S33 HSQC of Compound **3** in CD_3OD. ... 18
Figure S34 HMBC of Compound **3** in CD_3OD. ... 19
Figure S35 ^{1}H-^{1}H COSY of Compound **3** in CD_3OD. ... 19
Figure S36 The NOE of Compound **3** in CD_3OD. ... 20
Figure S37 The crystal packing of Compound **3** ... 20
Figure S38 ^{1}H-NMR of Compound **4** in CD_3OD, 400 MHz ... 21
Figure S39 ^{13}C-NMR and DEPT of Compound **4** in CD_3OD, 100 MHz ... 21
Figure S40 ORTEP View and crystal packing of Compound **4** ... 22
Table 1. Crystal data and structure refinement for Compound **4** ... 23
Figure S41. ^{1}H-NMR of Compound **5** in CD_3Cl, 400 MHz ... 23
Figure S42. ^{13}C-NMR of Compound **5** in CD_3Cl, 100 MHz ... 24
Figure S43 ORTEP View and crystal packing of Compound **5** ... 24
Table 2. Crystal data and structure refinement for Compound **5** ... 25
Figure S44. ^{1}H-NMR of Compound **6** in CD_3Cl, 400 MHz ... 26
Figure S45. ^{13}C-NMR of Compound **6** in CD_3Cl, 100 MHz ... 26
Check CIF of compound **1** ... 27
Check CIF of compound **1a** ... 31
Check CIF of compound **2** ... 35
Check CIF of compound **2a** ... 38
Check CIF of compound **3** ... 41
Check CIF of compound **4** ... 45
Check CIF of compound **5** ... 49

Figure S1. HRESIMS of compound **1**

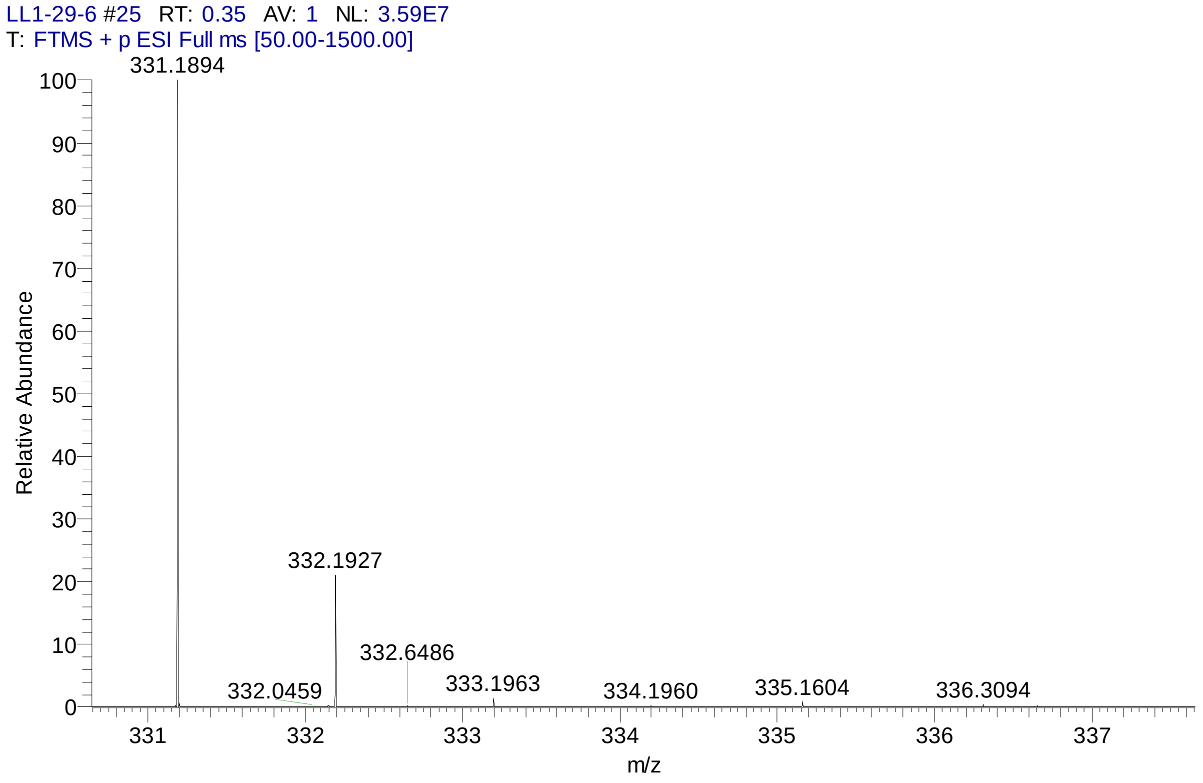

Figure S2. The UV spectrum of compound **1**

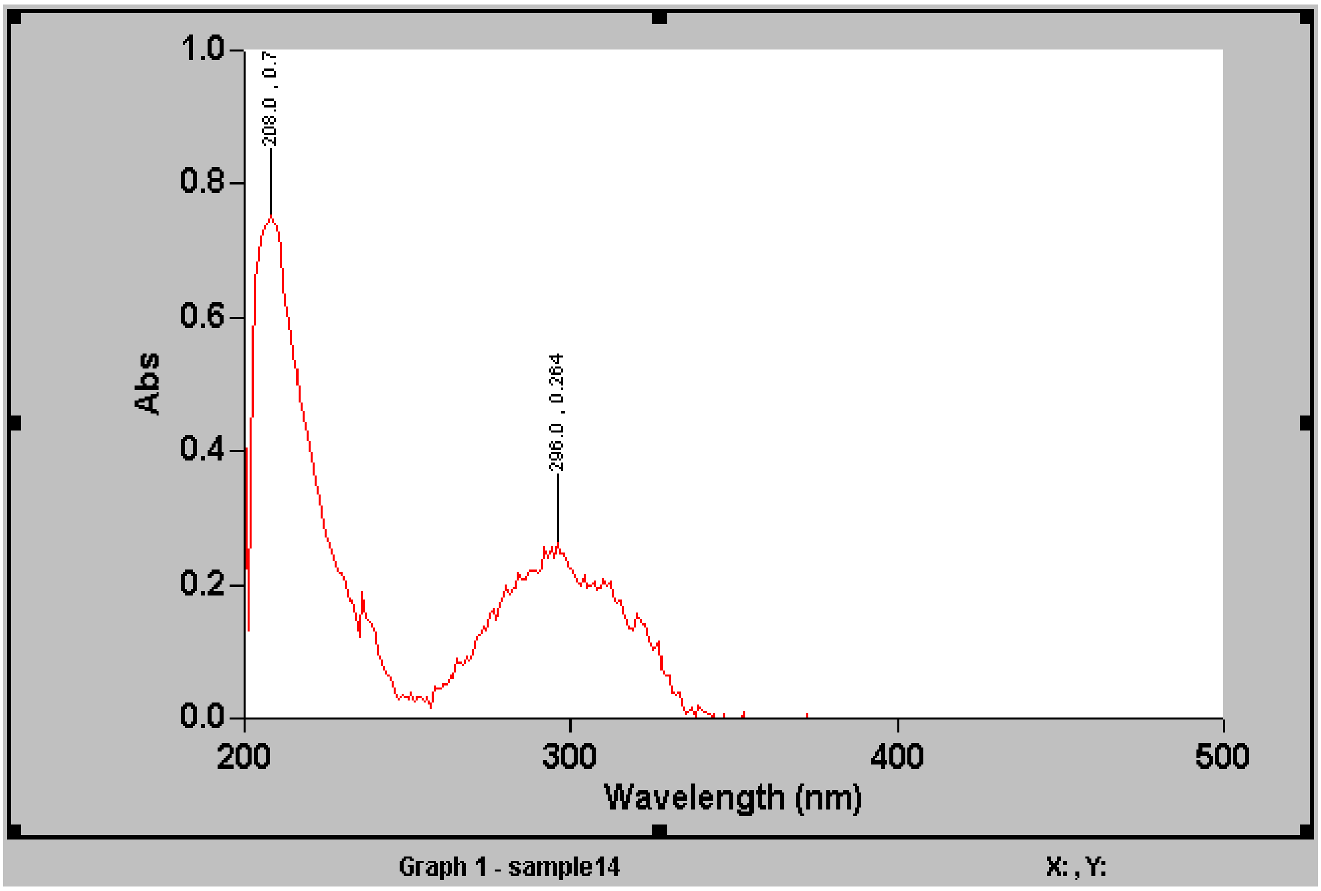

Figure S3. IR spectrum of Compound **1**

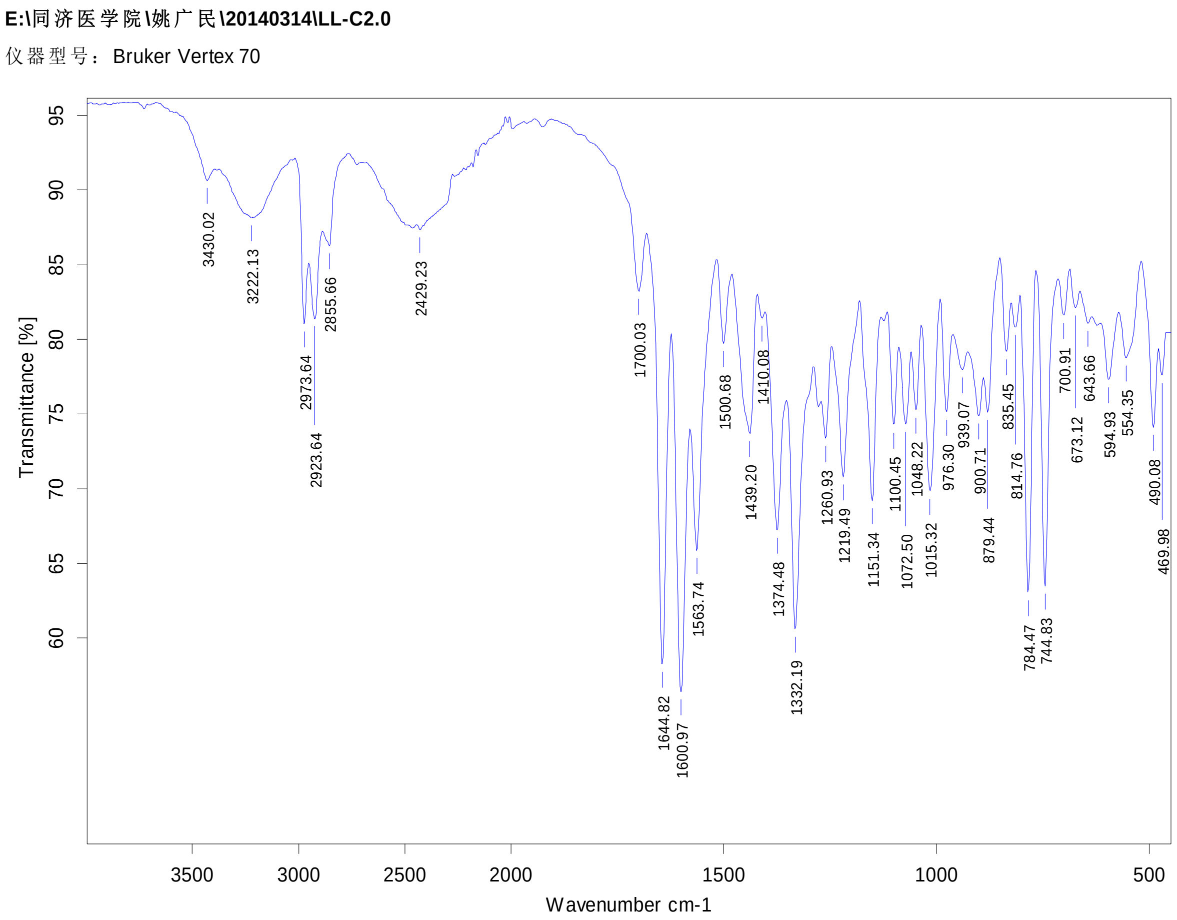

Figure S4. ^{1}H-NMR of Compound **1** in Pyridine-d_5, 400 MHz

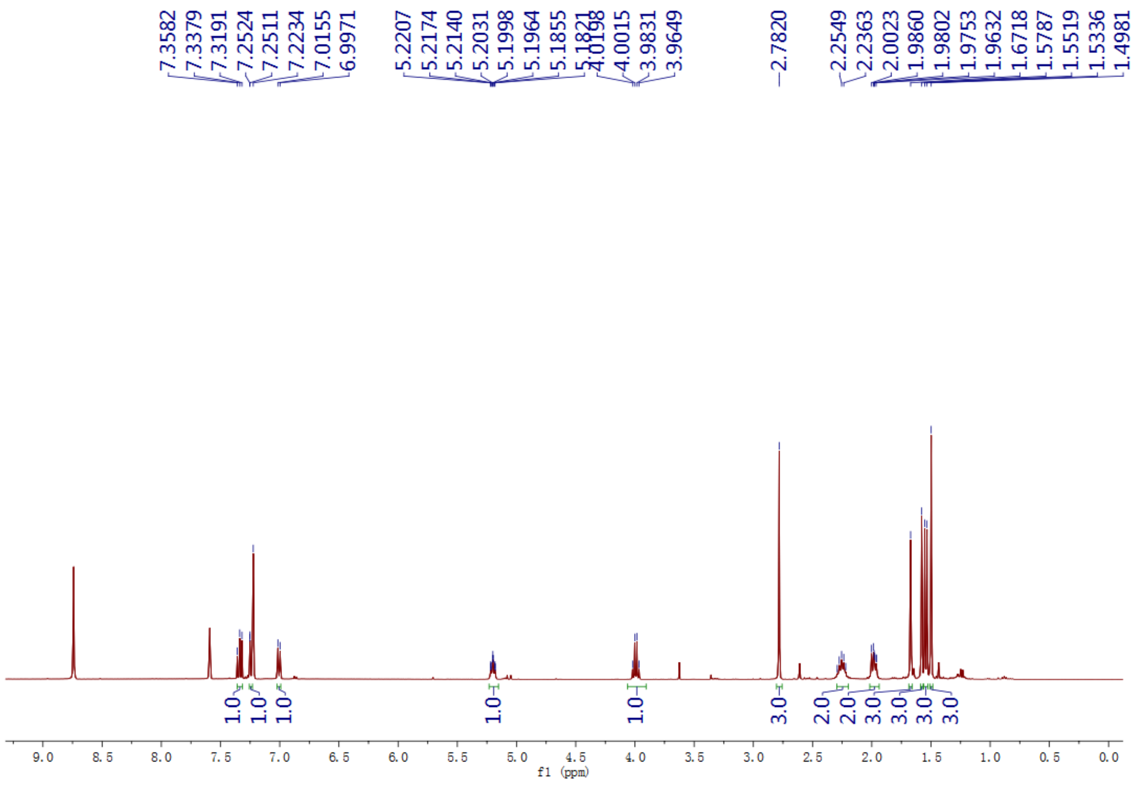

Figure S5. ^{13}C-NMR and DEPT NMR of Compound **1** in Pyridine-d$_5$, 100 MHz

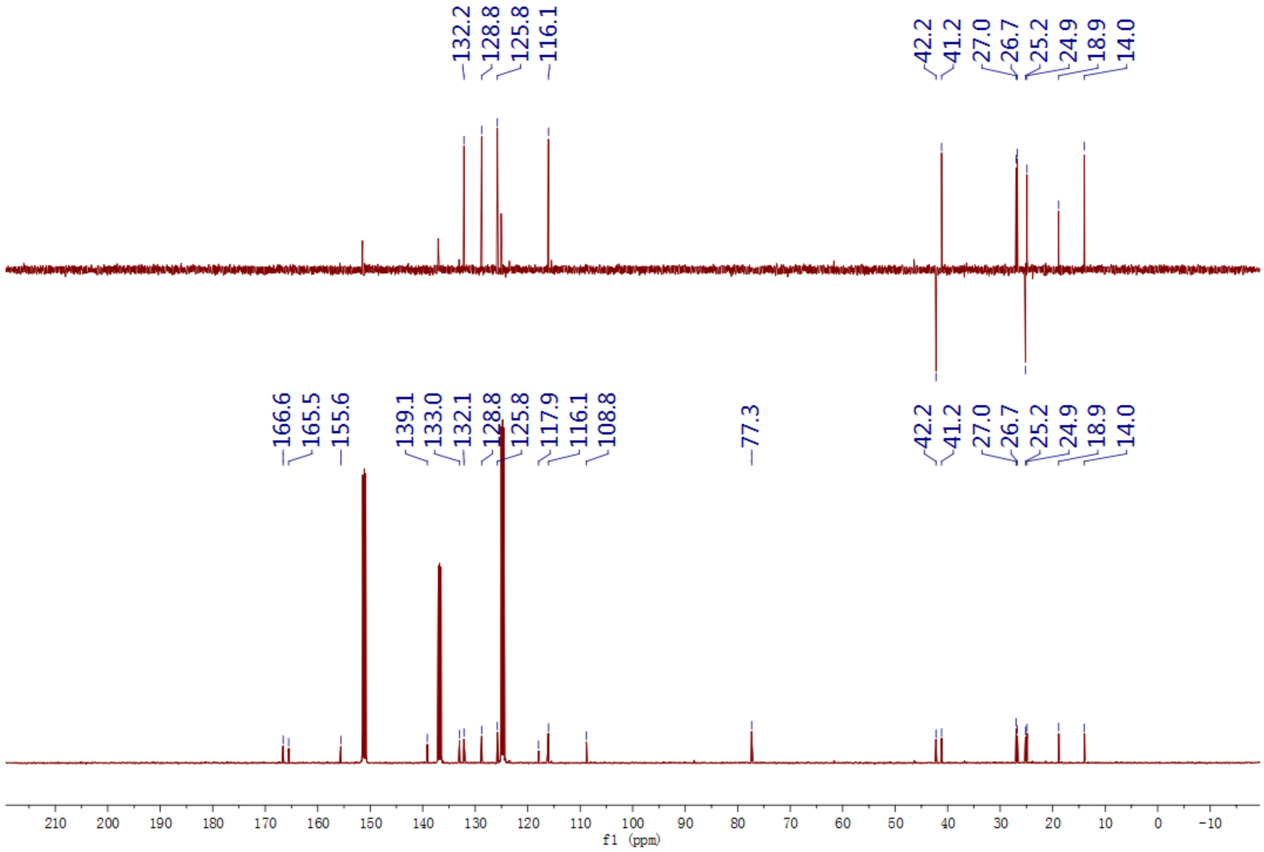

Figure S6. HSQC of Compound **1** in Pyridine-d_5

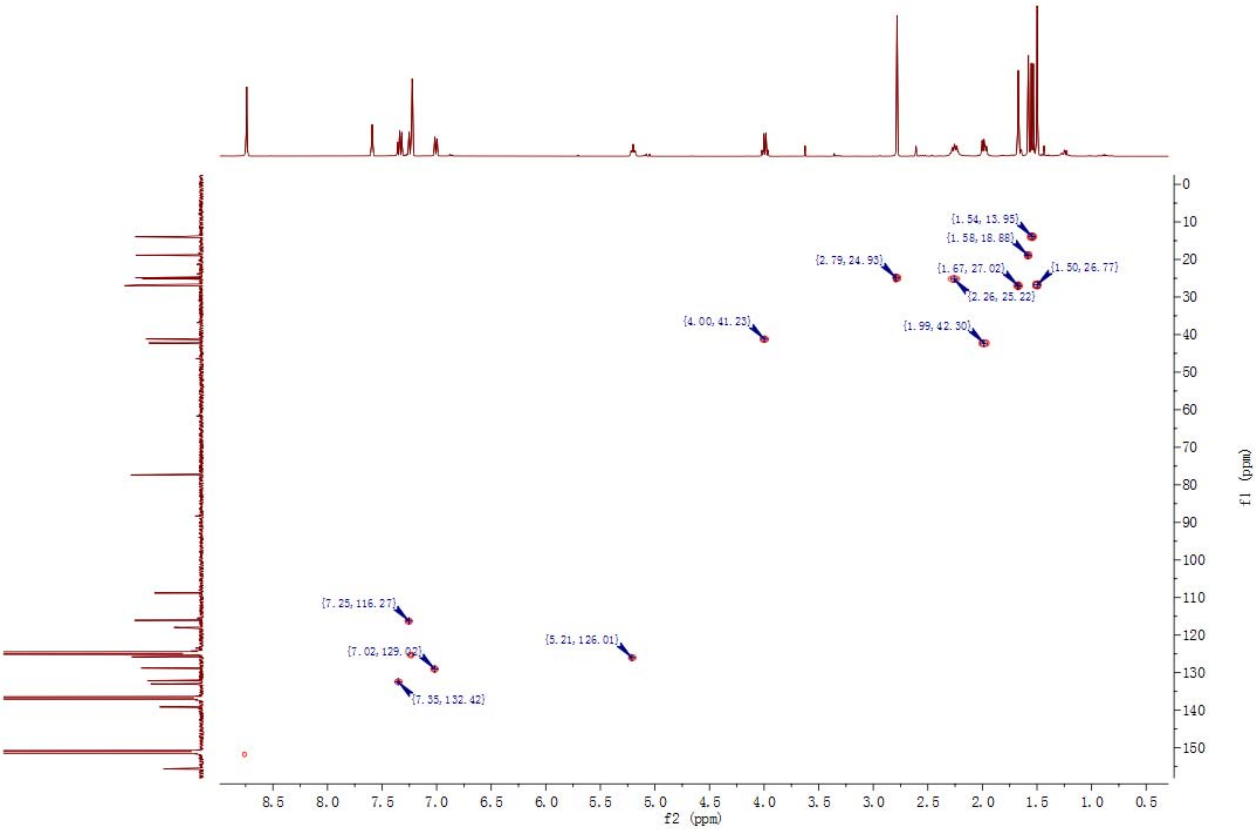

Figure S7. HMBC of Compound **1** in Pyridine-d_5

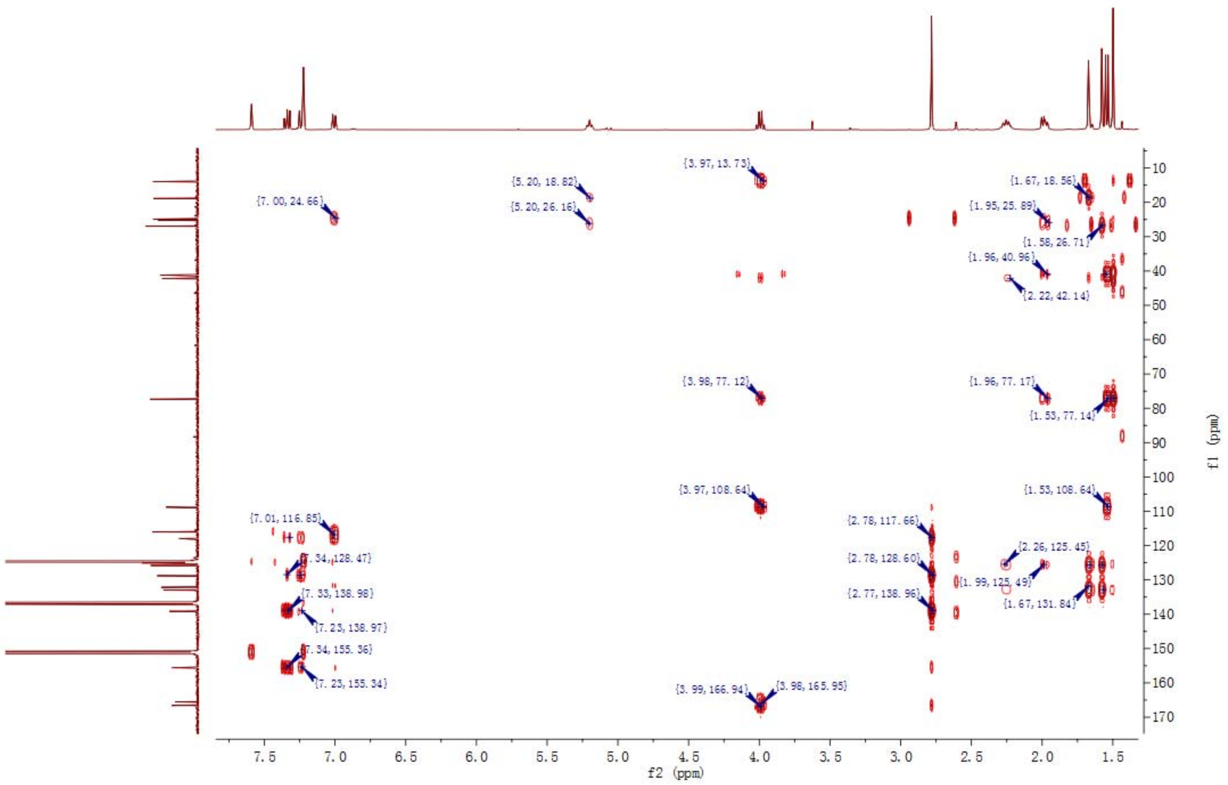

Figure S8. ^{1}H-^{1}H COSY of Compound **1** in Pyridine-d_5

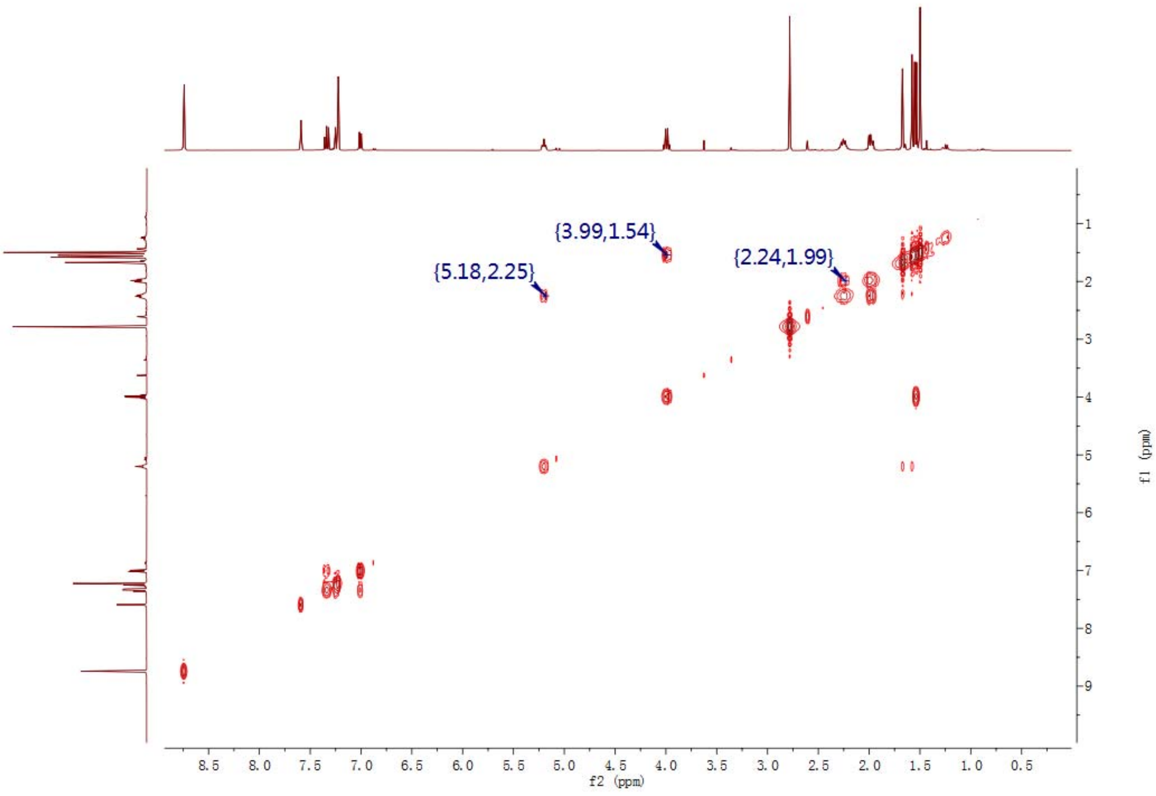

Figure S9. NOESY spectrum of Compound **1** in Pyridine-d_5

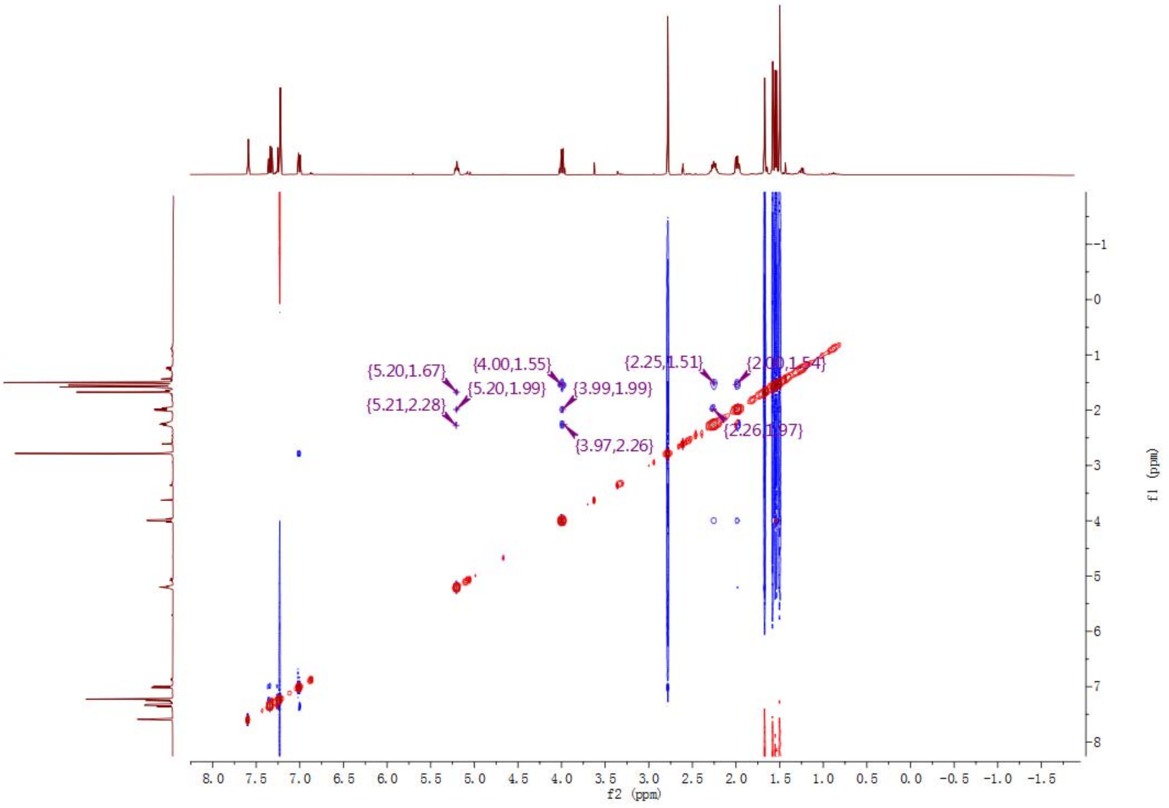

Figure S10 ^{1}H-^{1}H COSY and Key HMBC correlations of compound **1**

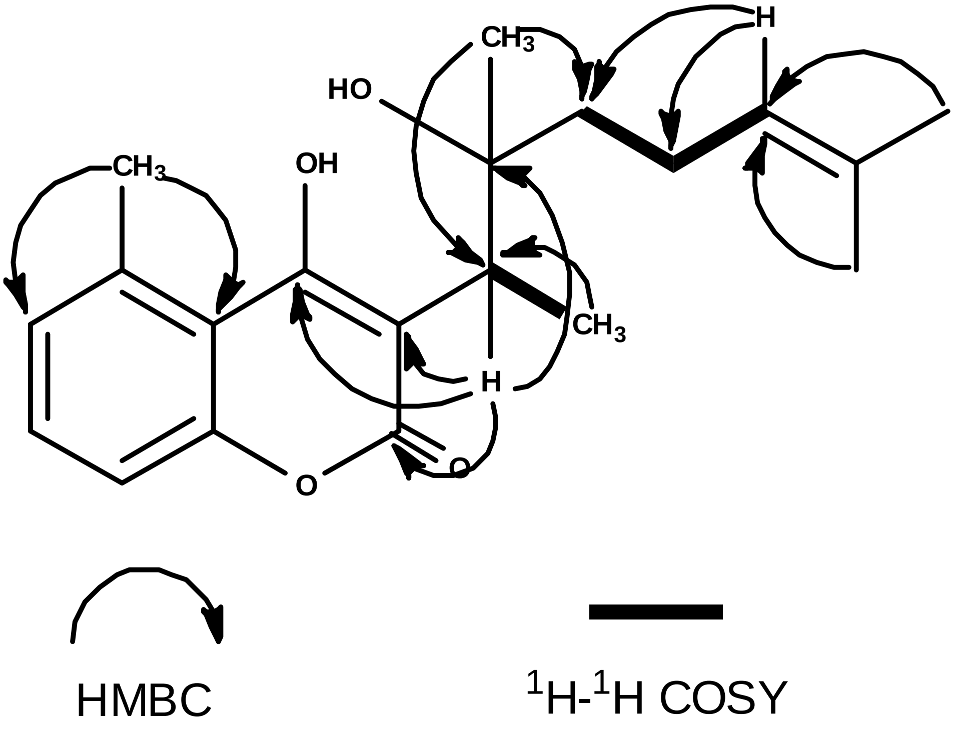

Figure S11. ORTEP View of Compound **1**.

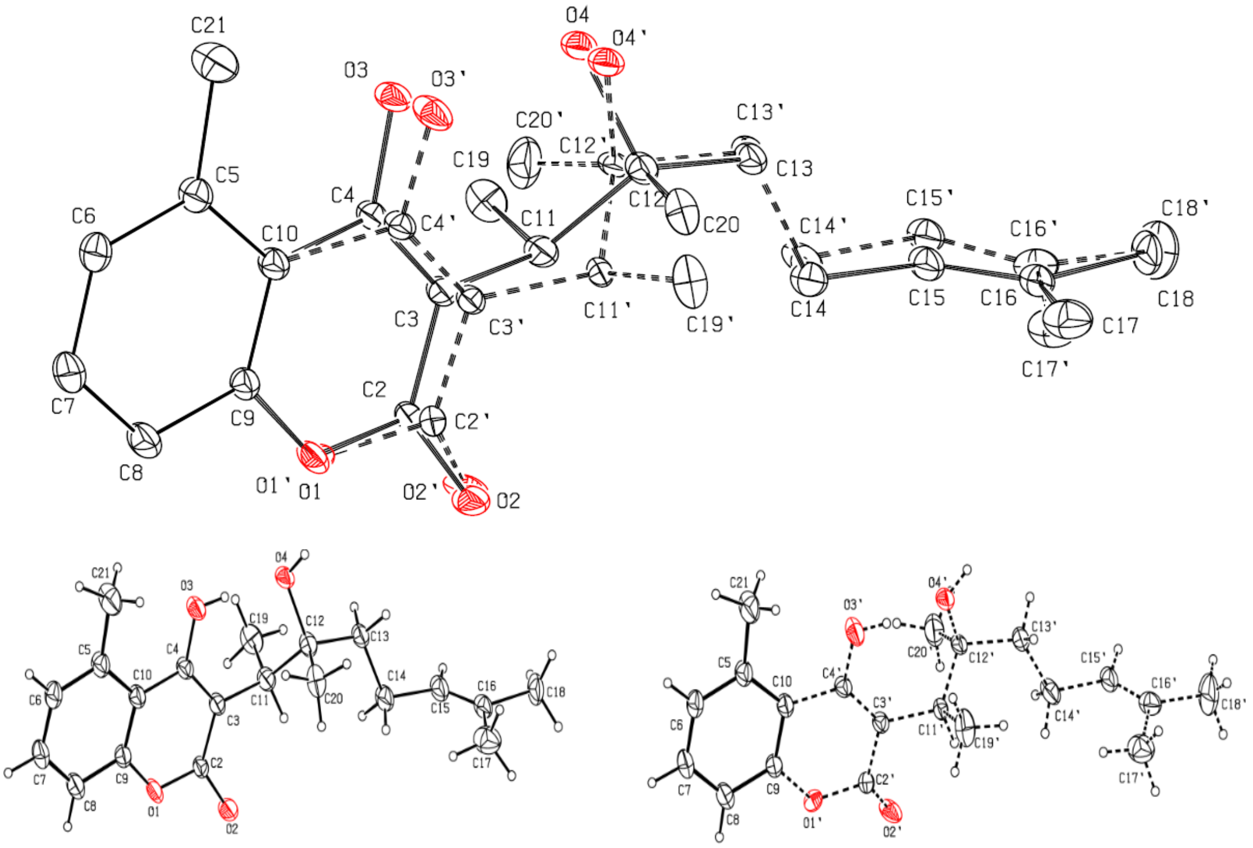

62.7% **37.3%**

Figure S12 Enantioseparation of Compound **1** by HPLC.

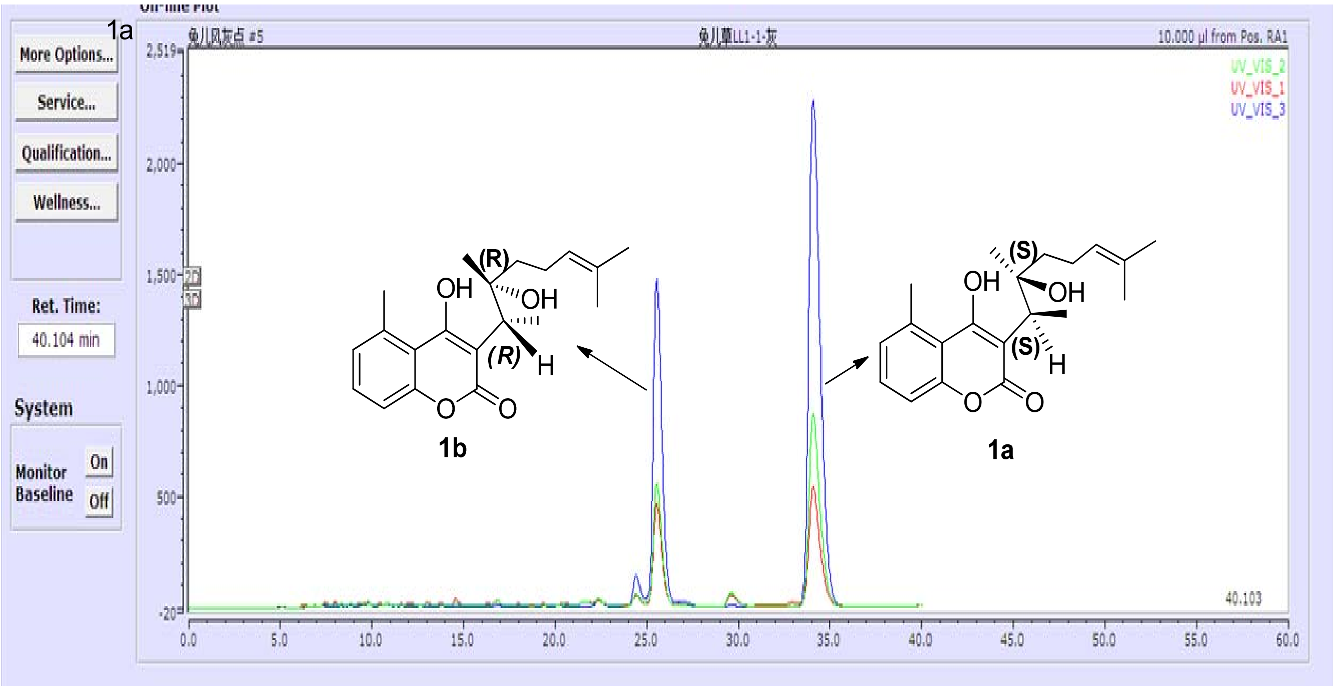

three-wavelength UV detector, the blue curve was at 230nm, green curve was at 210nm, the red curve was at 254nm.

Figure S13 The crystal packing of Compound **1a**

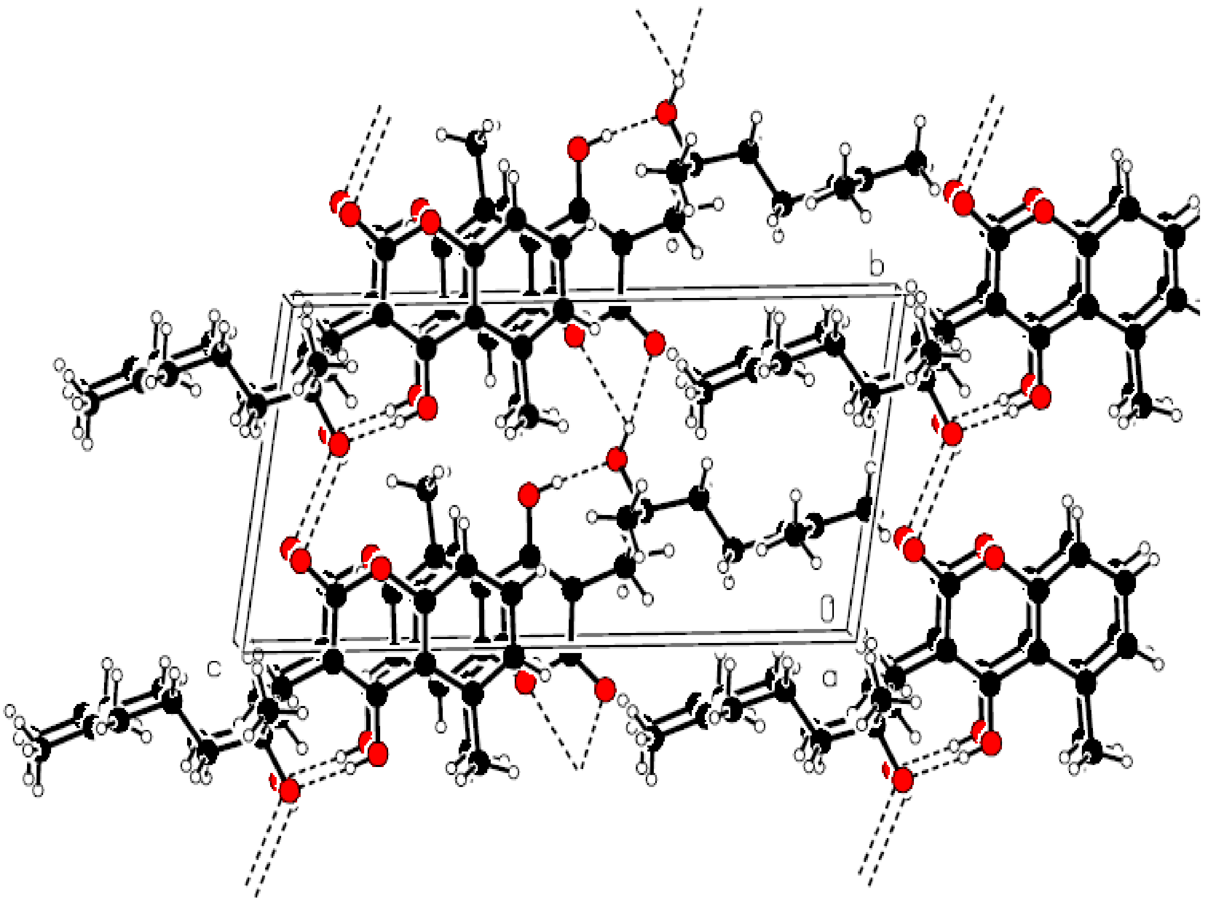

Figure S14 ORTEP View of Compound **1a.**

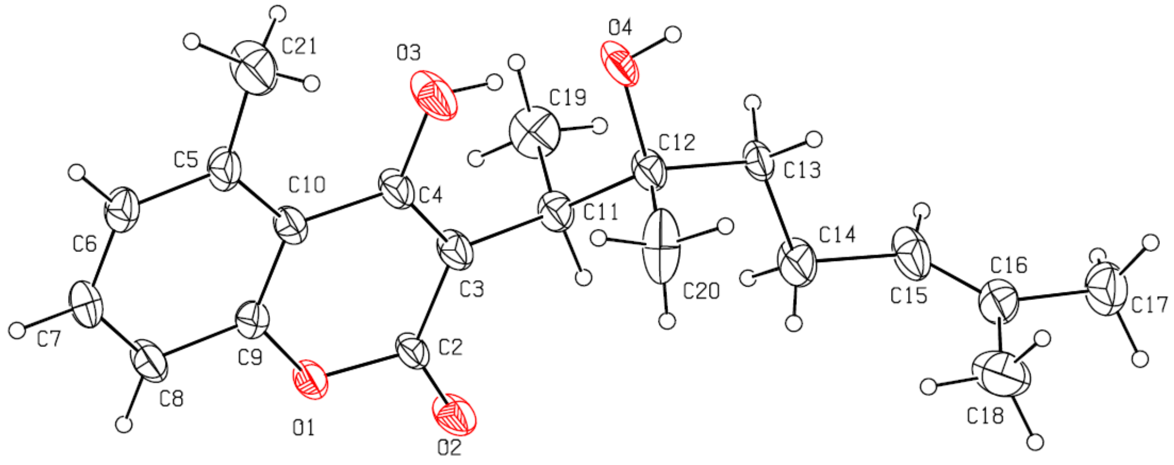

Figure S15 HRESIMS of compound **2**

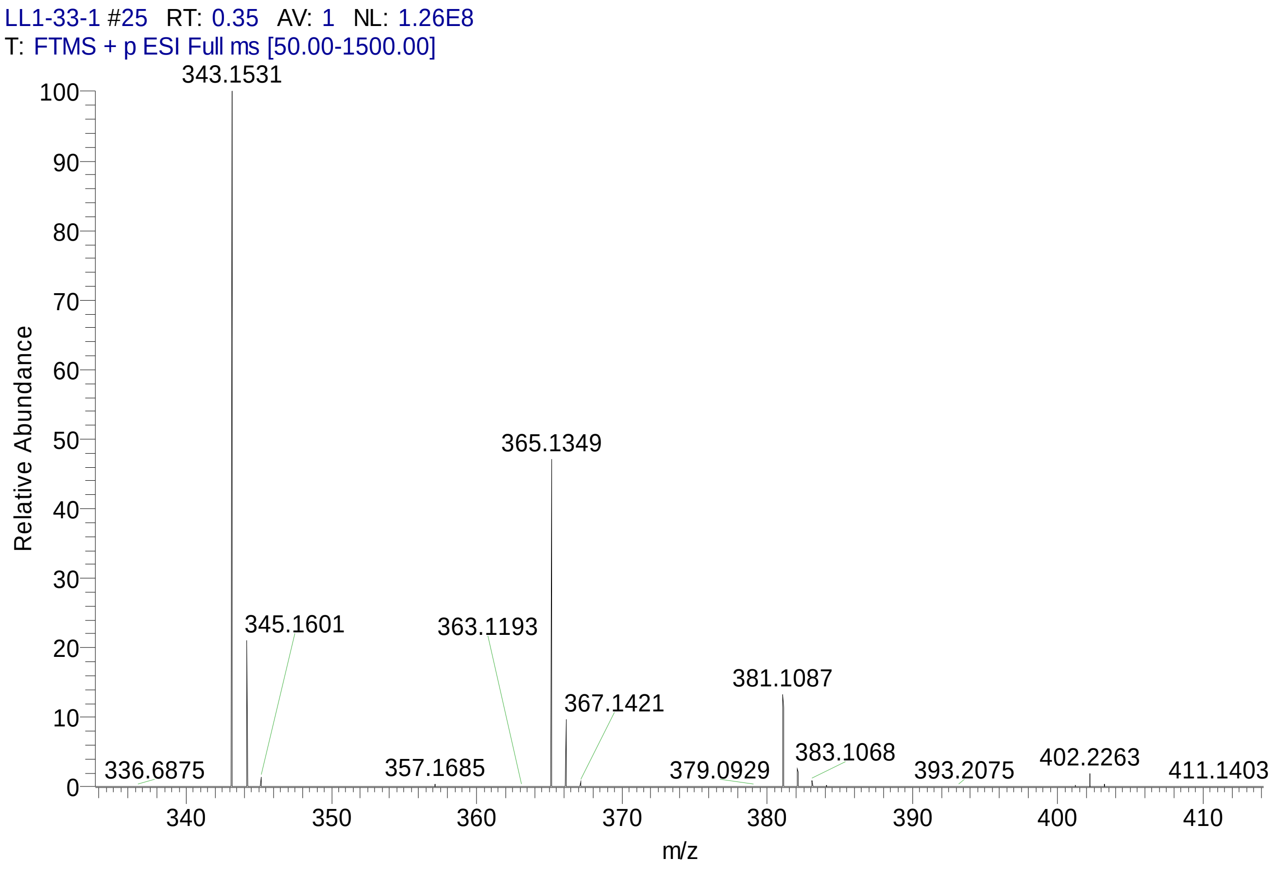

Figure S16 The UV spectrum of compound **2**

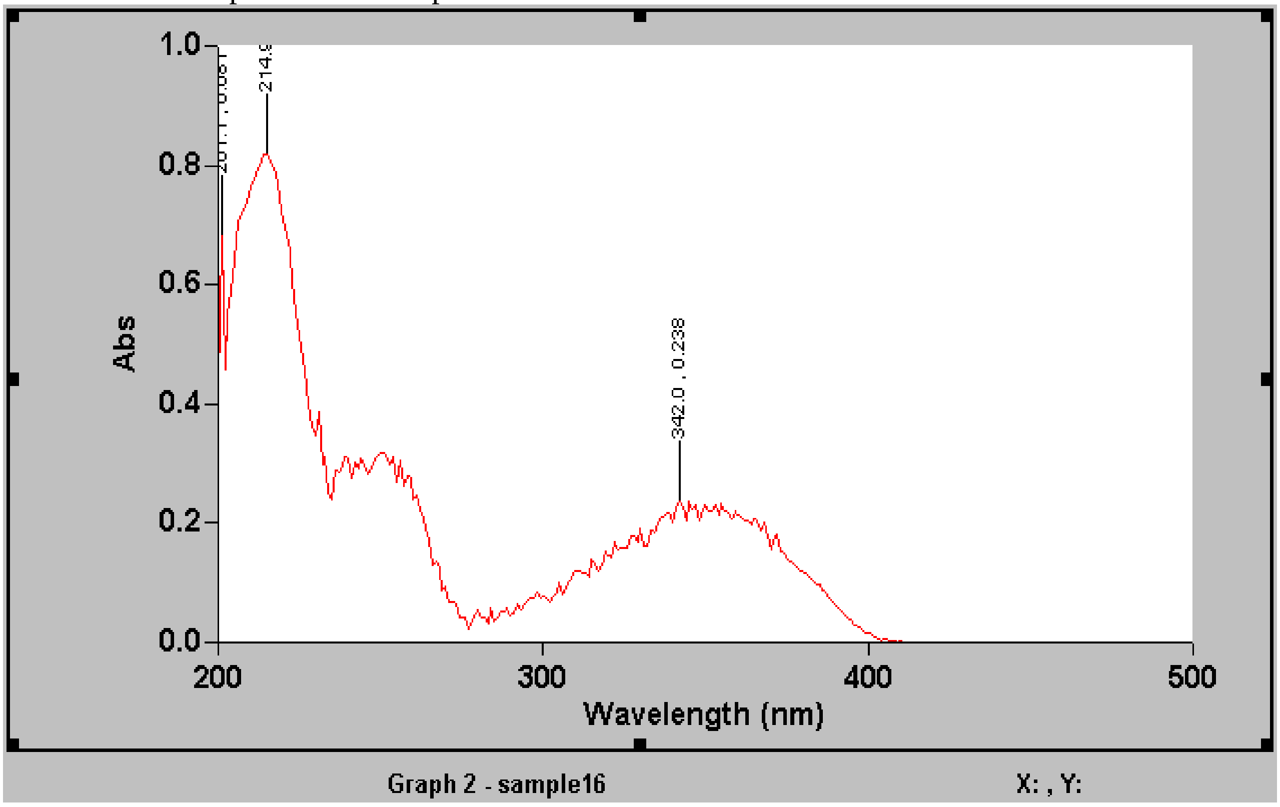

Figure S17 The IR spectrum of compound **2**

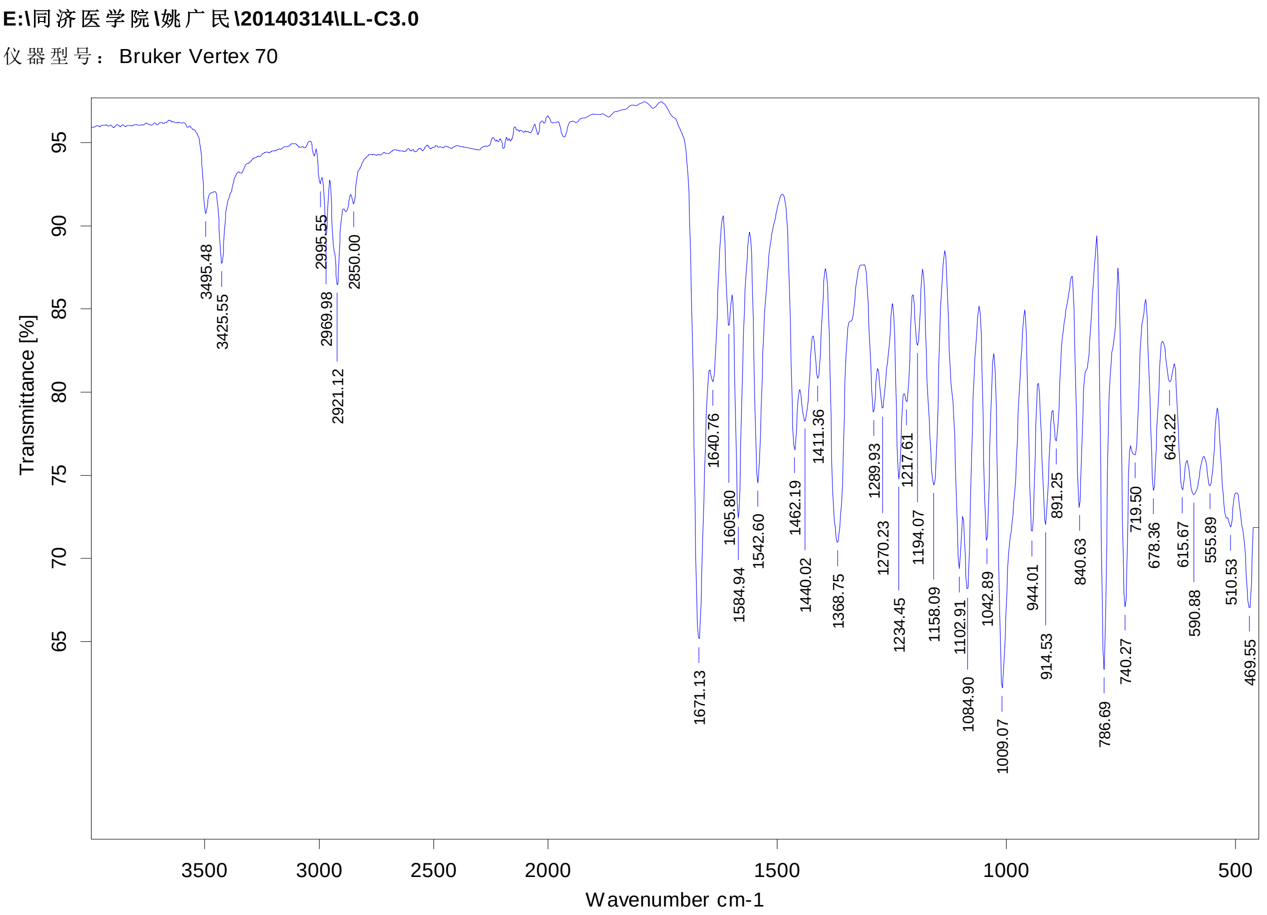

Figure S18 ^{1}H-NMR of Compound **2** in CD_3Cl, 400 MHz

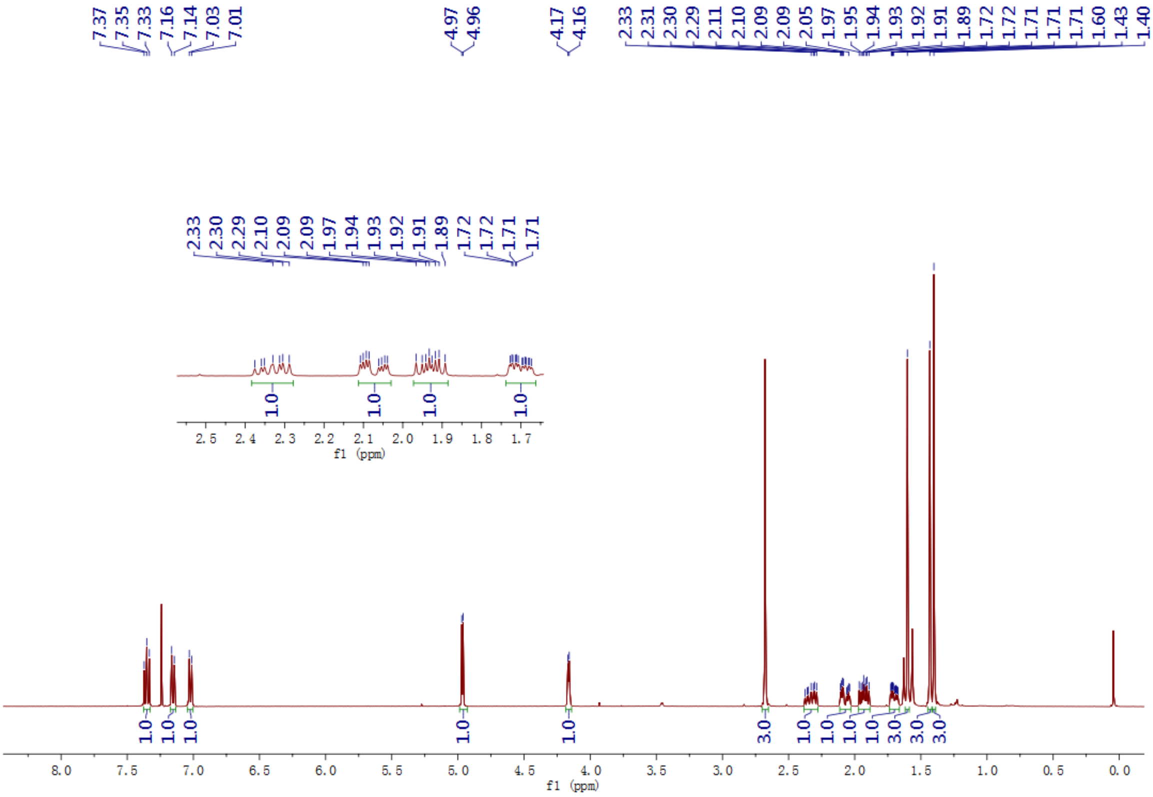

Figure S19^{13}C-NMR and DEPT of Compound **2** in CD_3Cl, 100 MHz

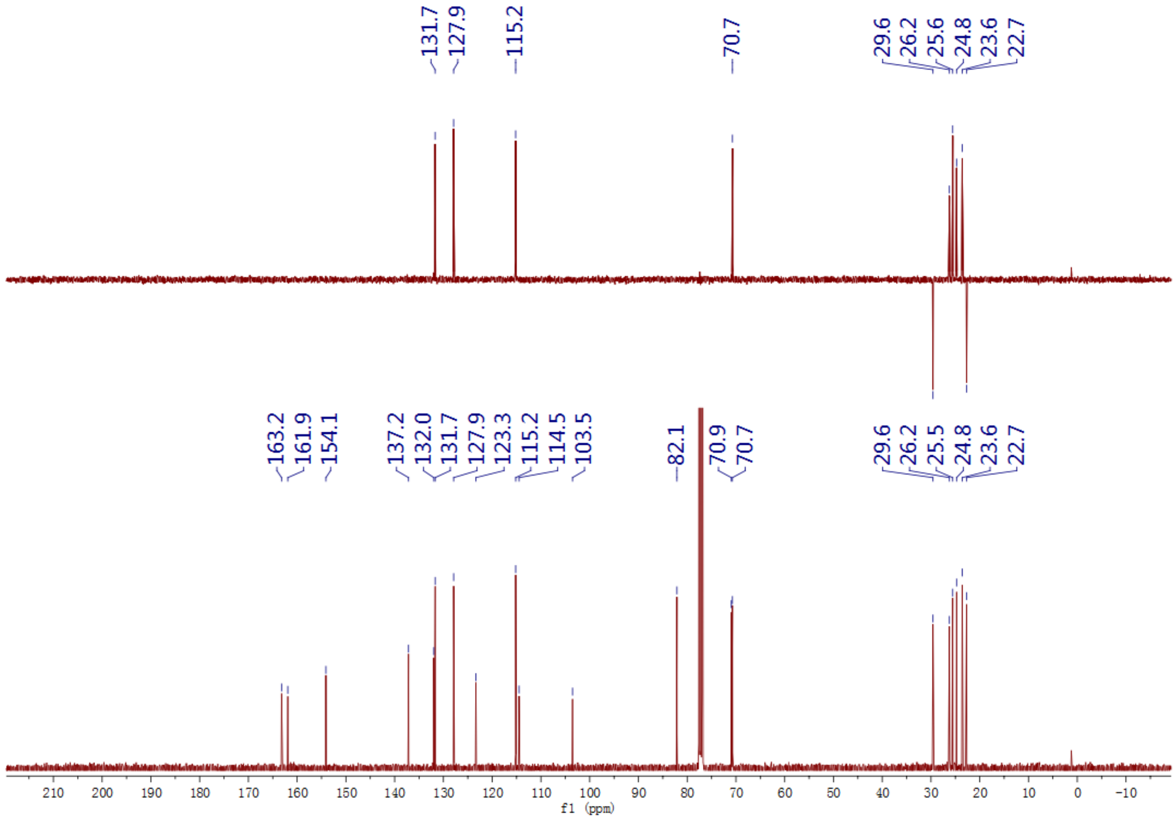

Figure S20 HSQC of Compound **2** in CD_3Cl.

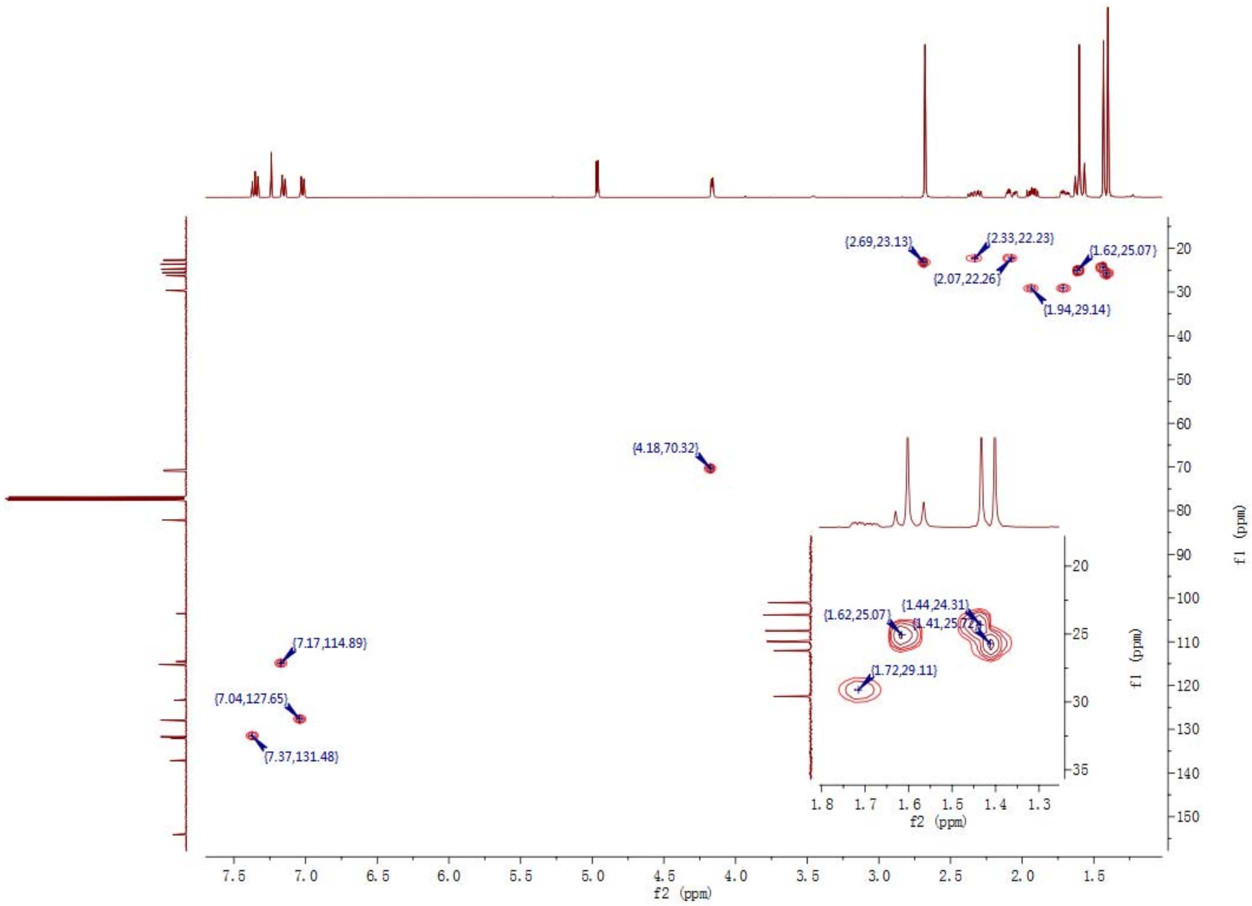

Figure S21 HMBC of Compound **2** in CD_3Cl.

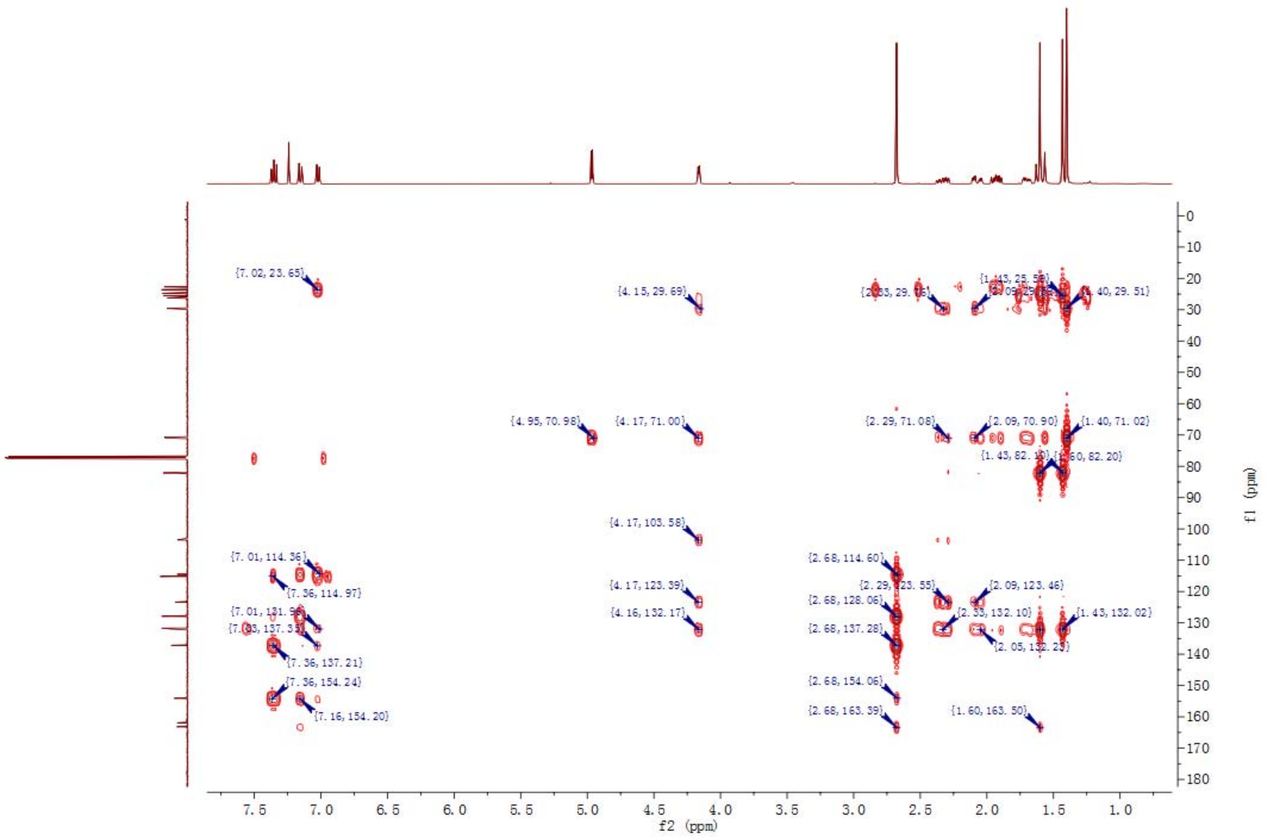

Figure S22 ^{1}H-^{1}H COSY of Compound **2** in CD_3Cl.

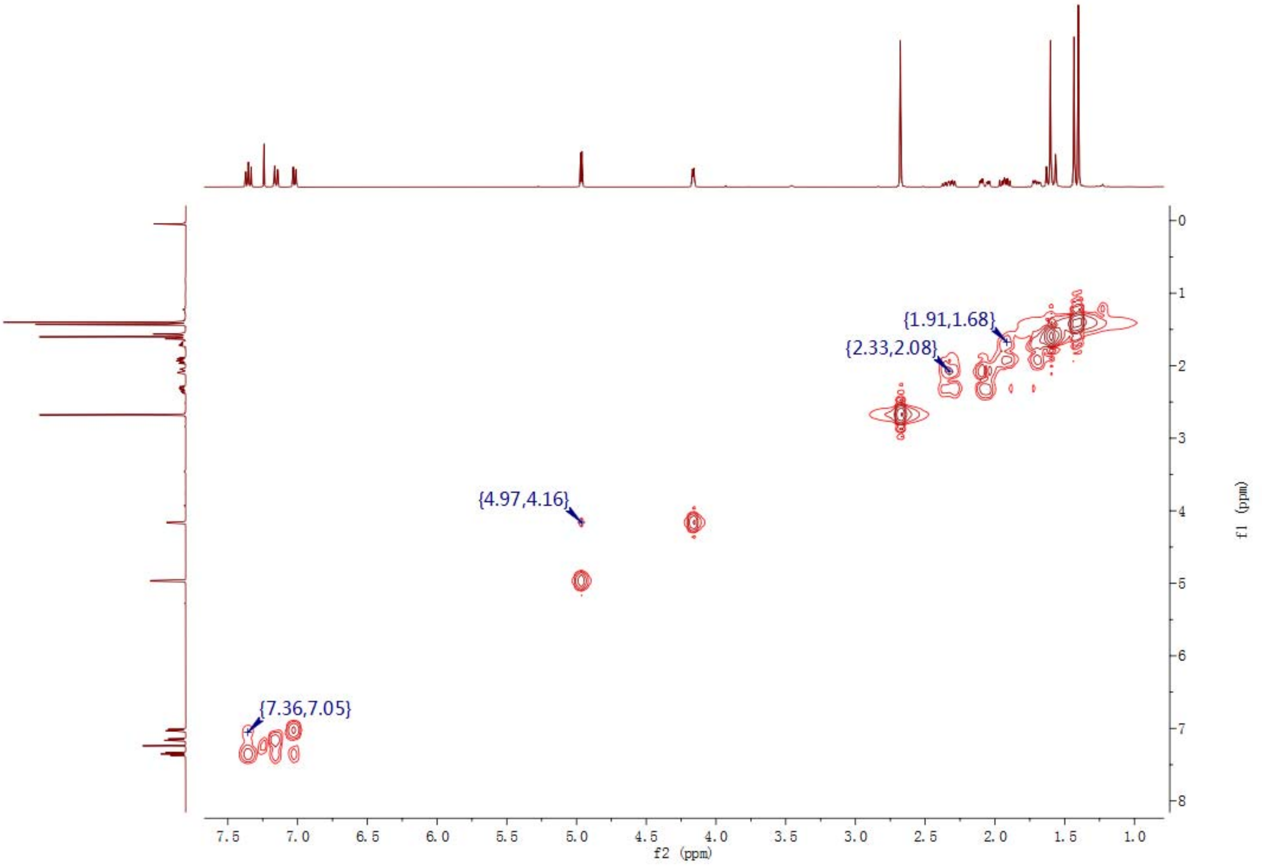

Figure S23 NOE of Compound **2** in CD_3Cl.

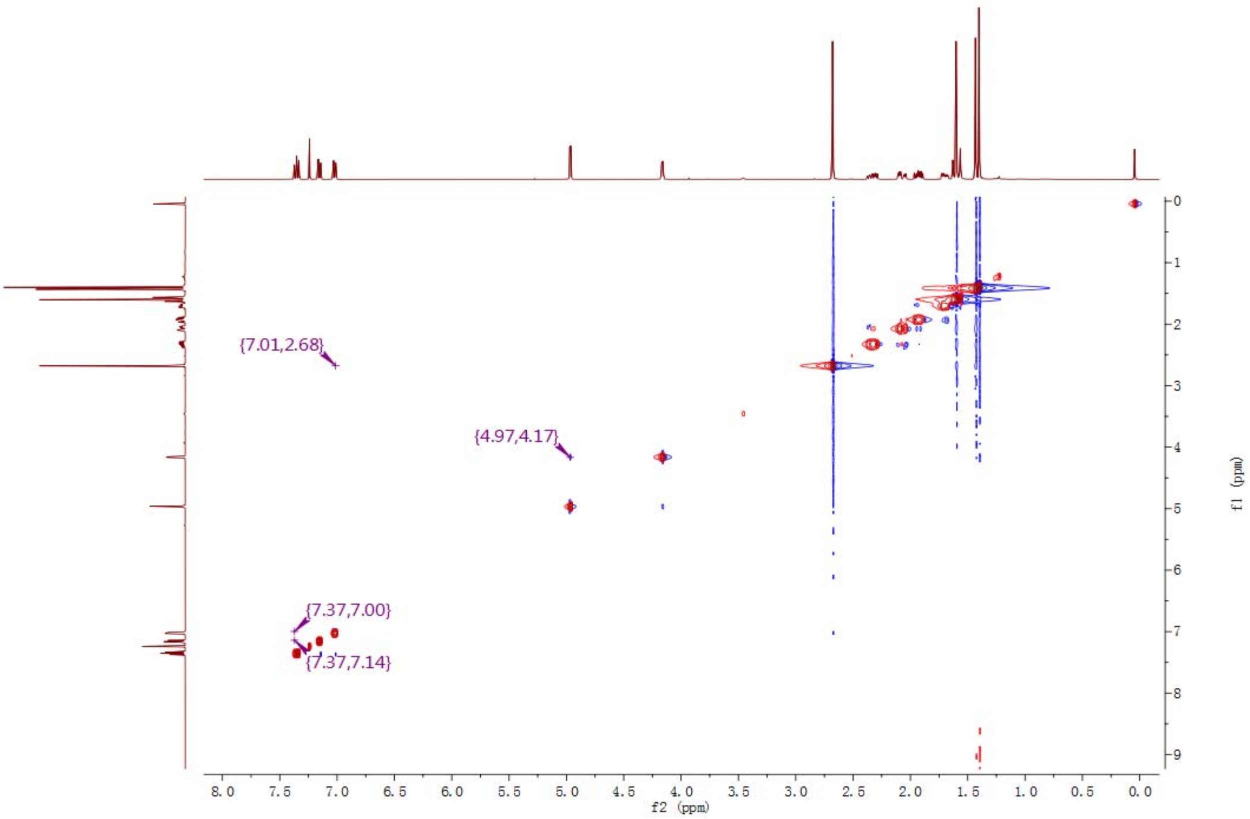

Figure S24 The crystal packing of Compound **2**

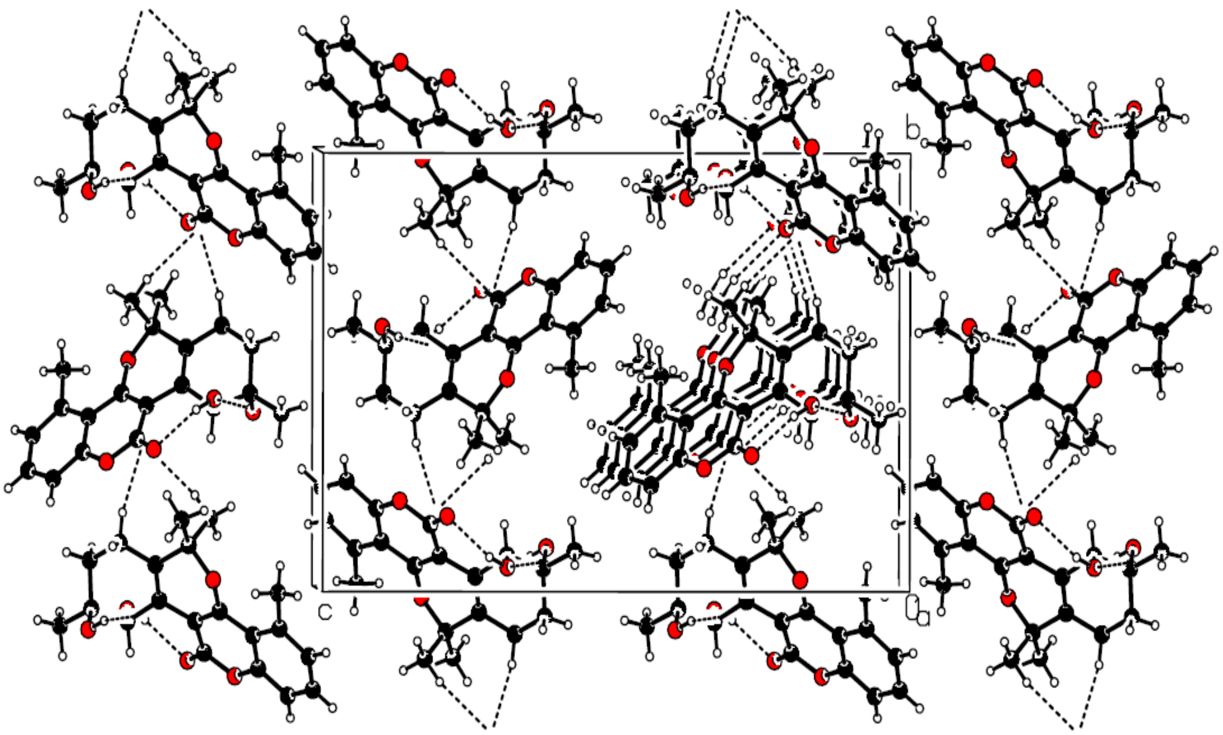

Figure S25 Enantioseparation of Compound **2** by HPLC.

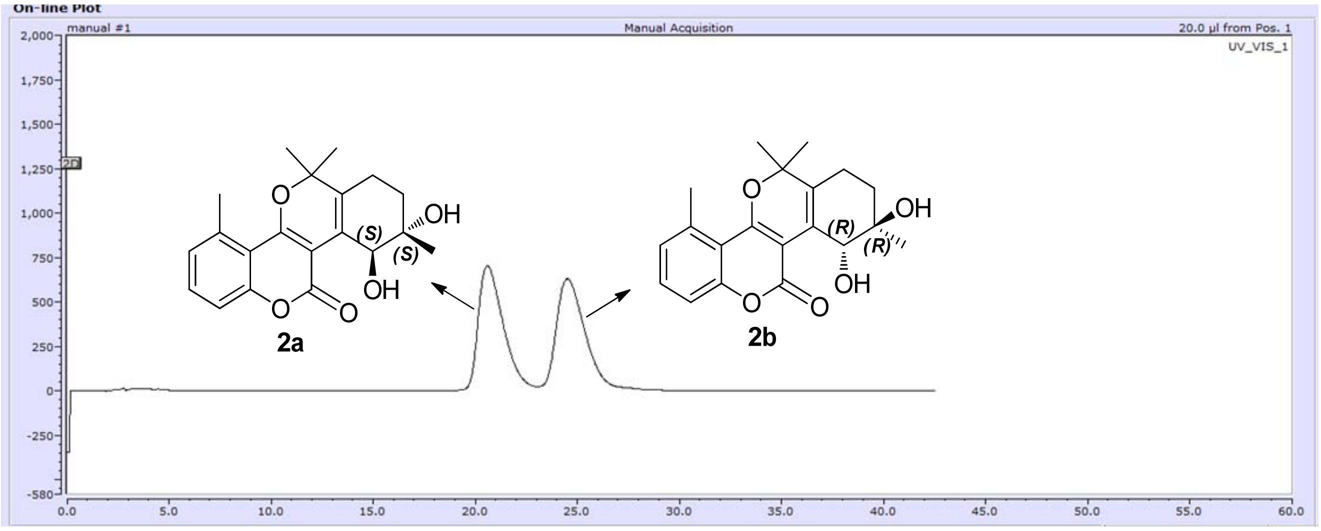

three-wavelength UV detector at 230nm

Figure S26 The crystal packing of Compound **2a**

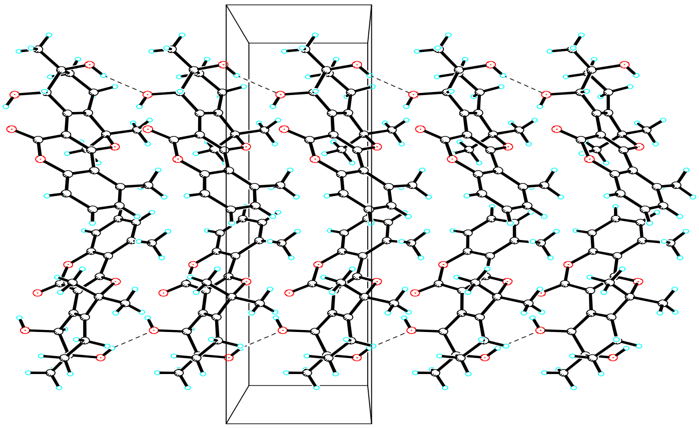

Figure S27 ORTEP View of Compound **2a**

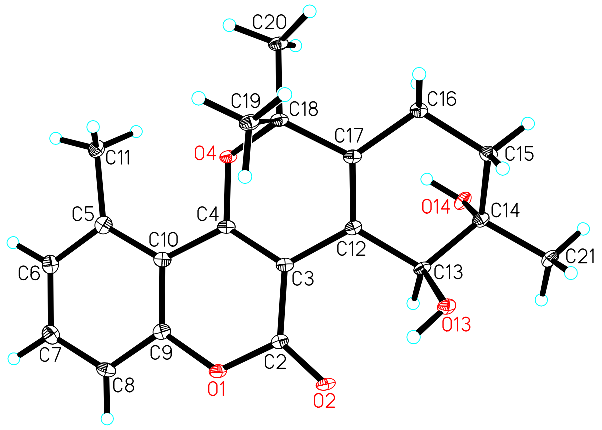

Figure S28 HRESIMS of compound **3**

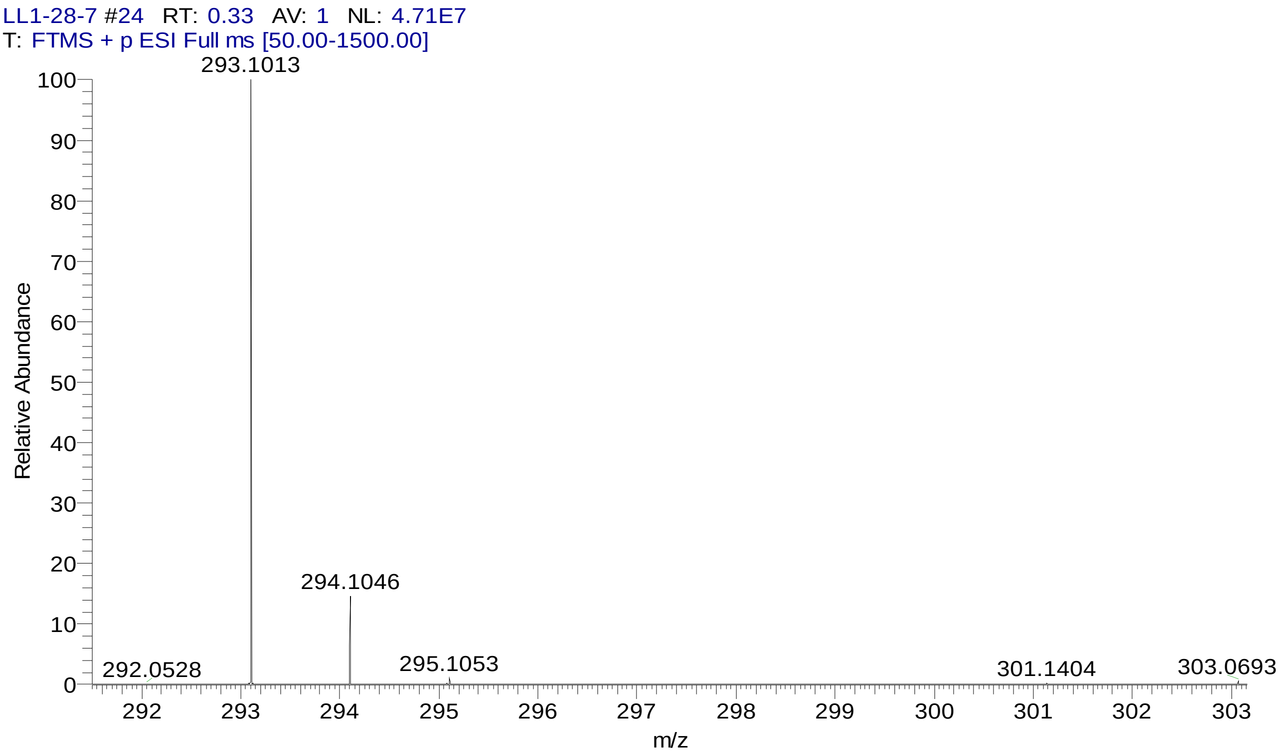

Figure S29 IR spectrum of Compound **3**

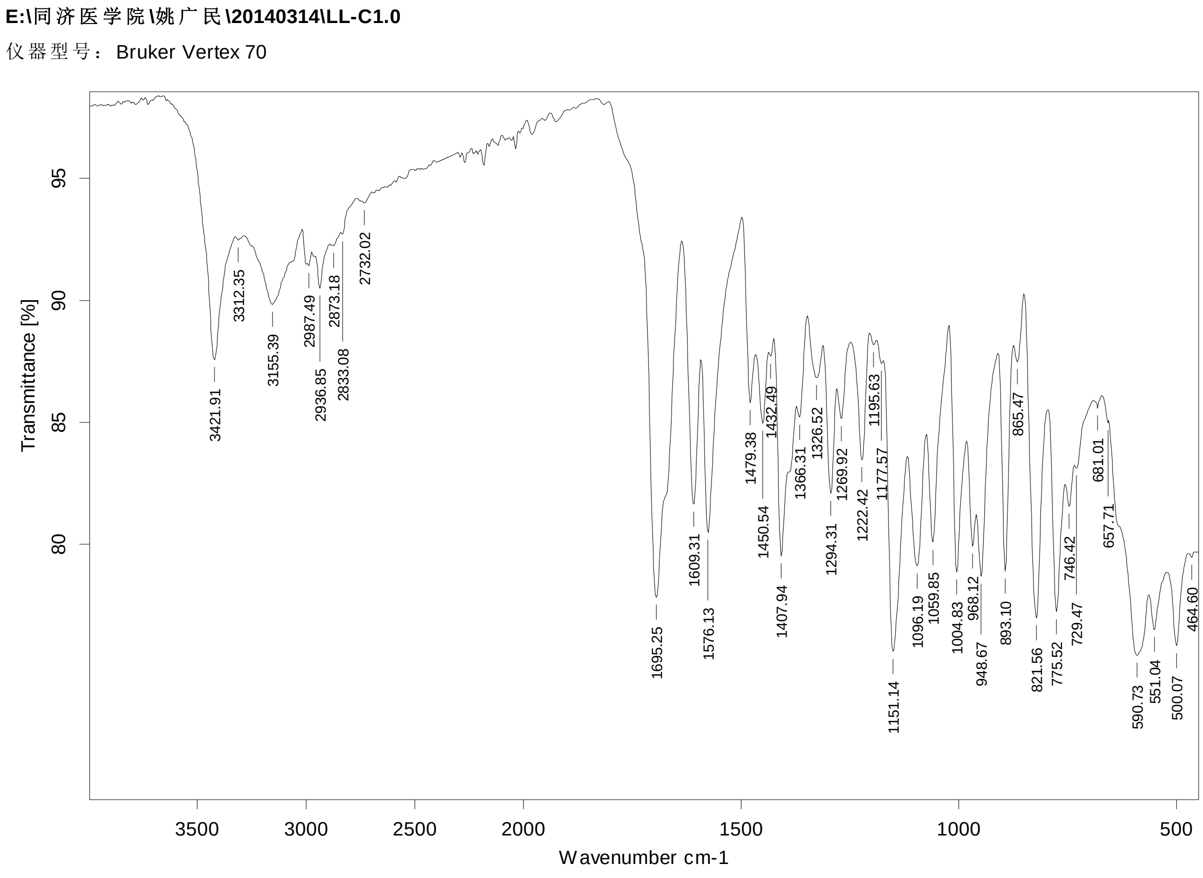

Figure S30UV spectrum of Compound **3**

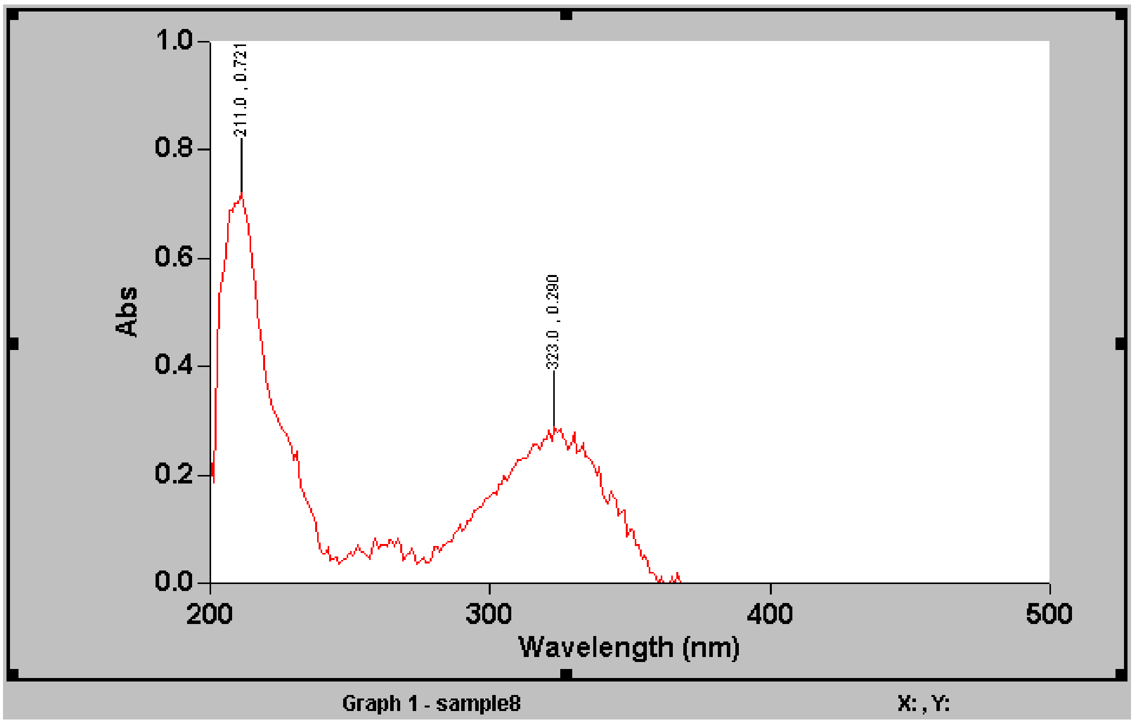

Figure S31 ^{1}H-NMR of Compound **3** in CD_3OD, 400 MHz

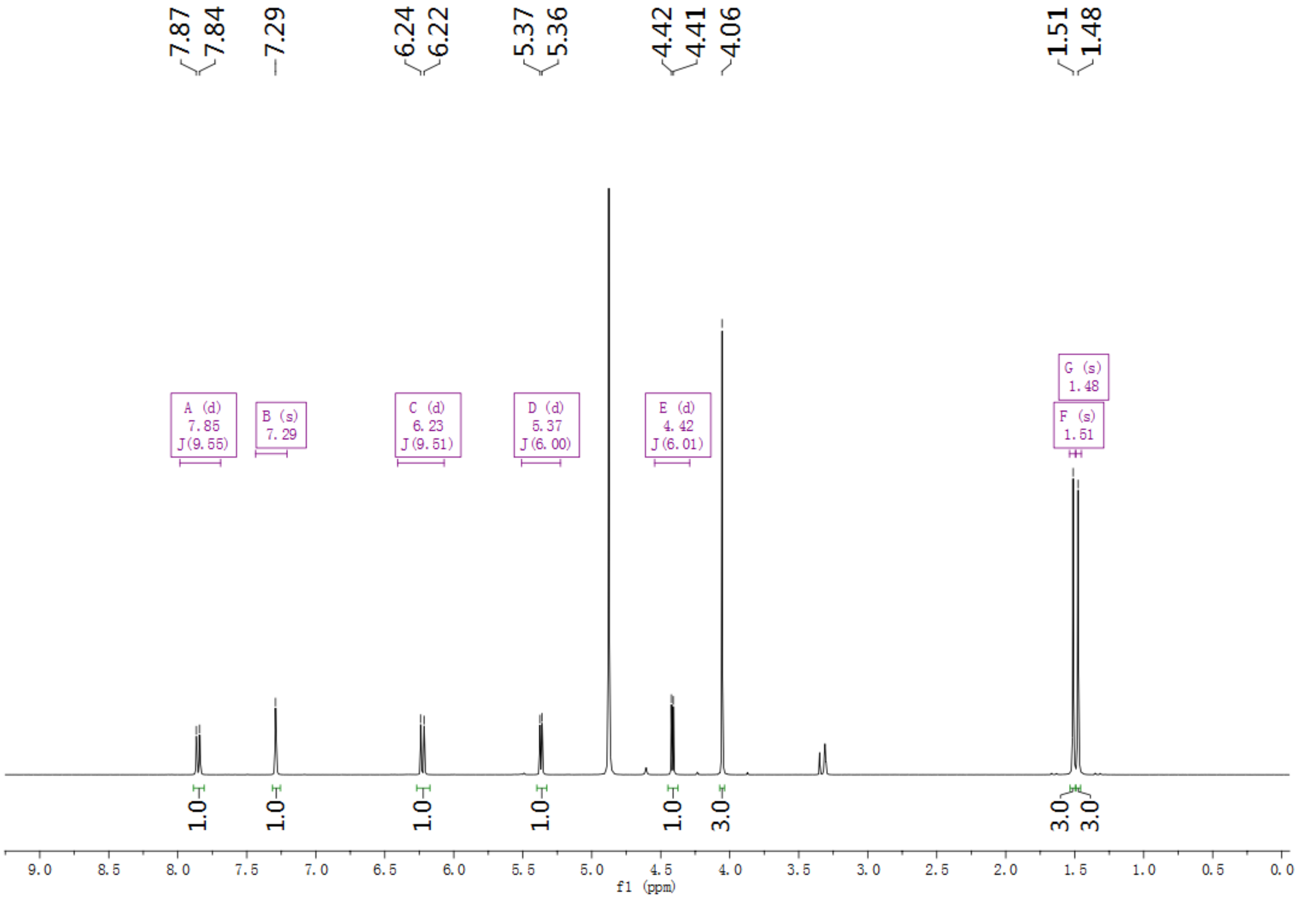

Figure S32 ^{13}C-NMR and DEPT of Compound **3** in CD_3OD, 100 MHz

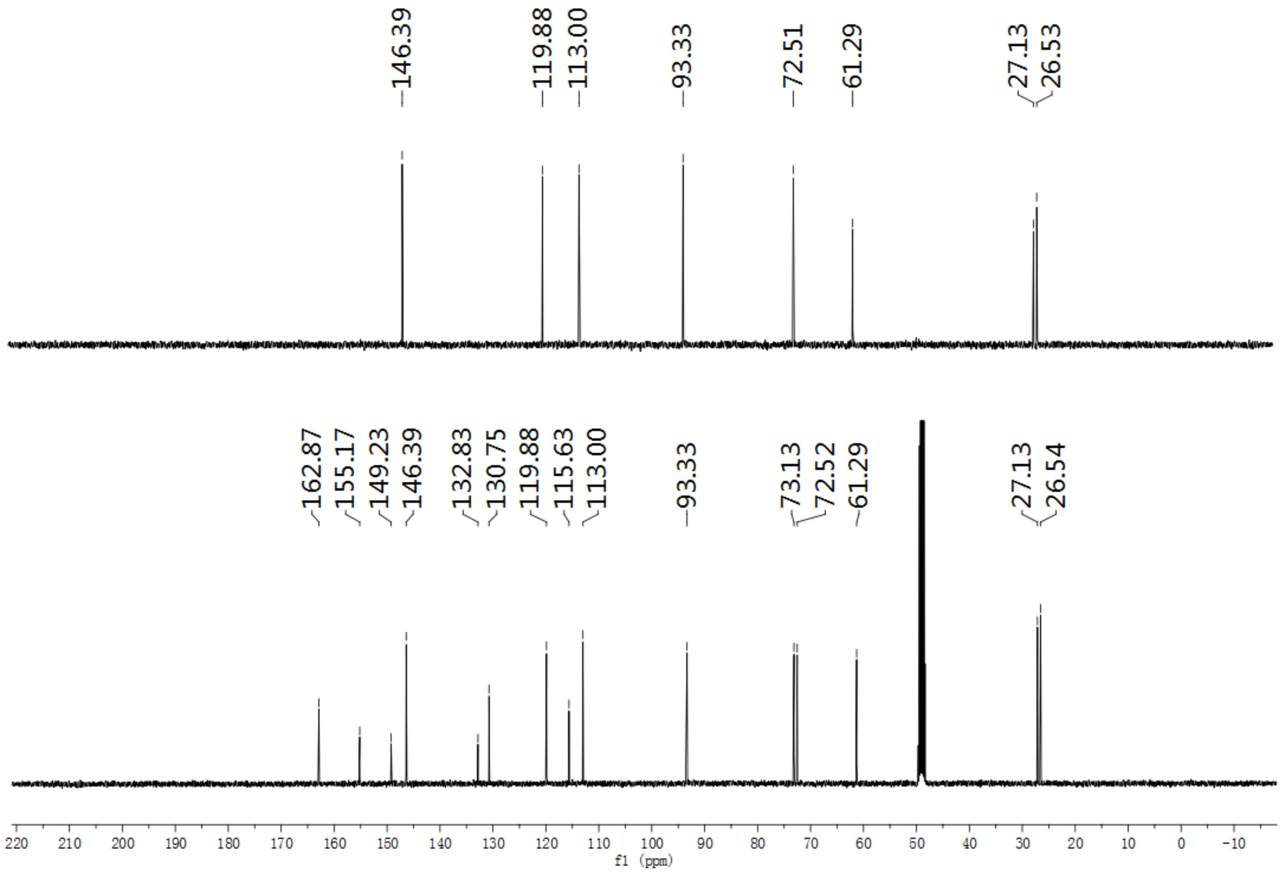

Figure S33 HSQC of Compound **3** in CD_3OD.

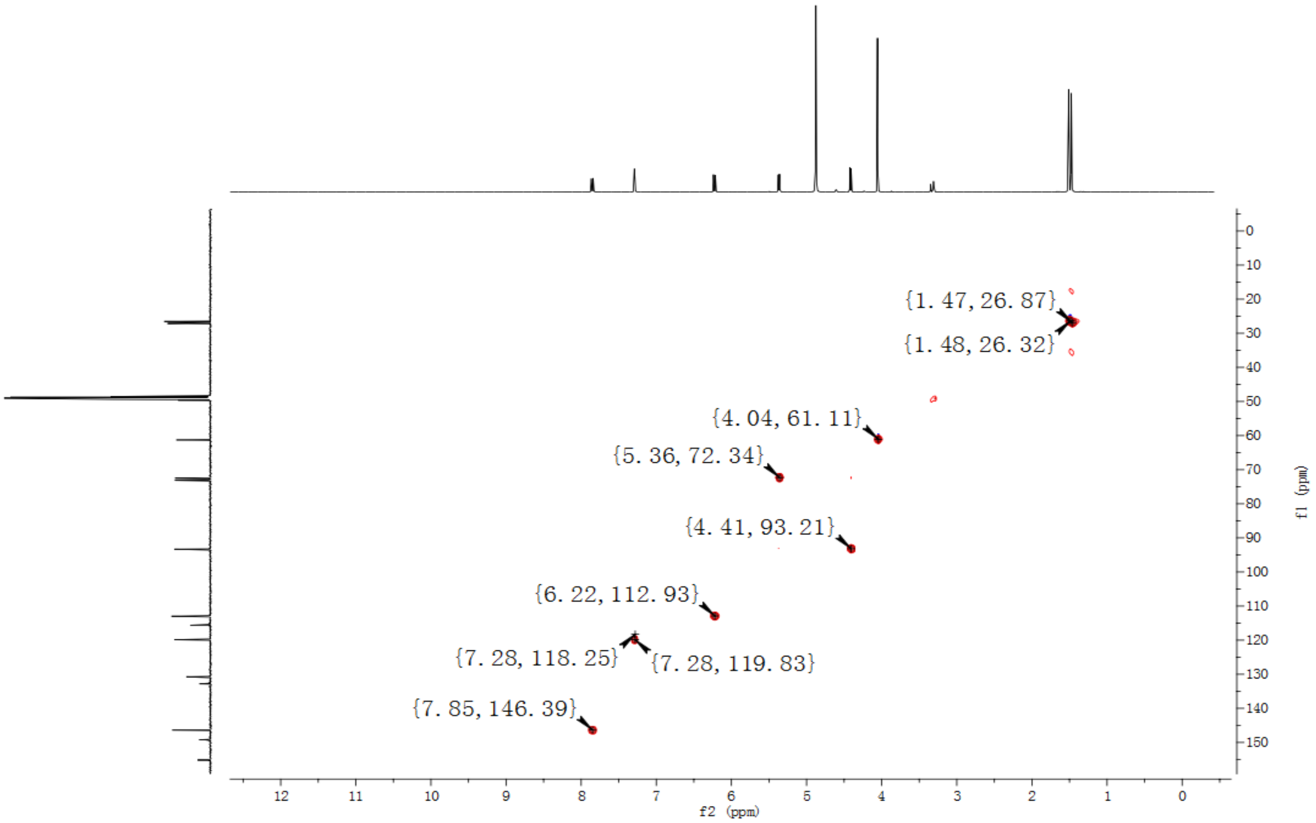

Figure S34 HMBC of Compound **3** in CD_3OD.

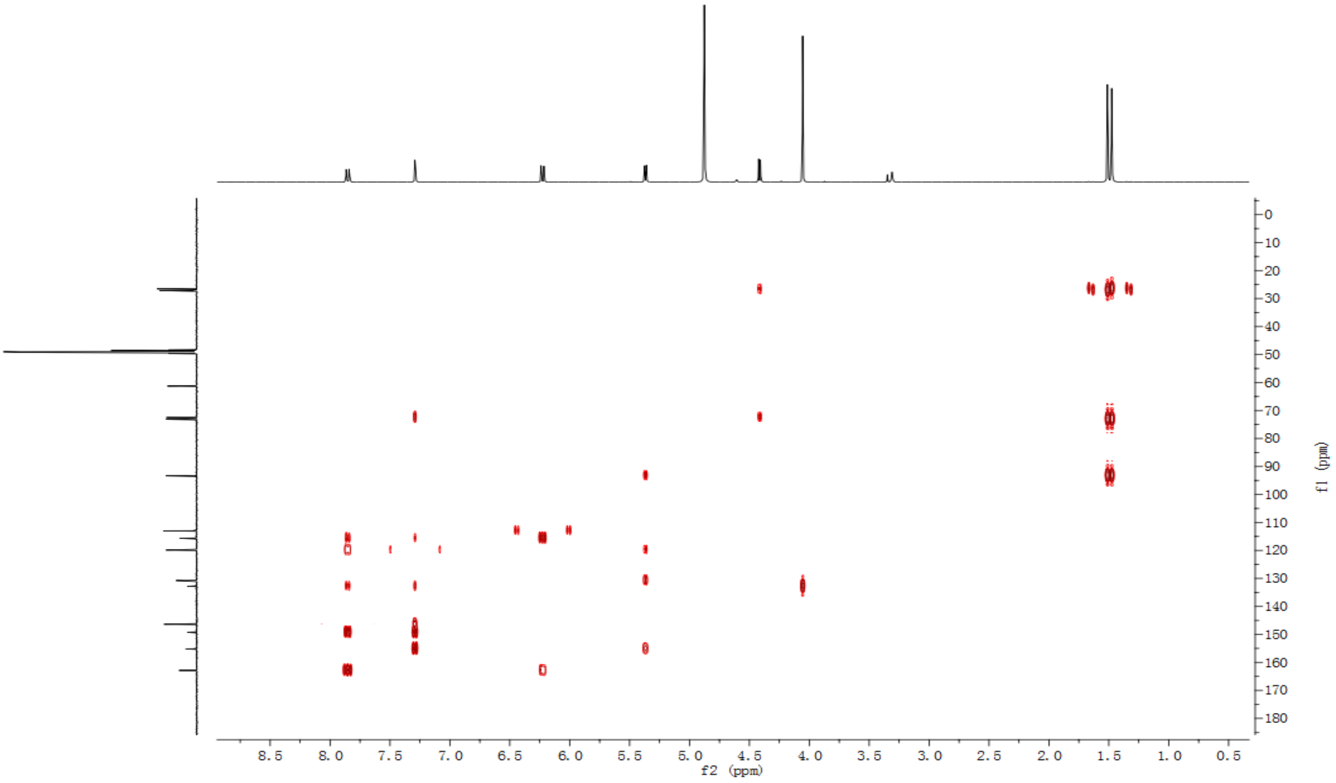

Figure S35 ^1H-^1H COSY of Compound **3** in CD_3OD.

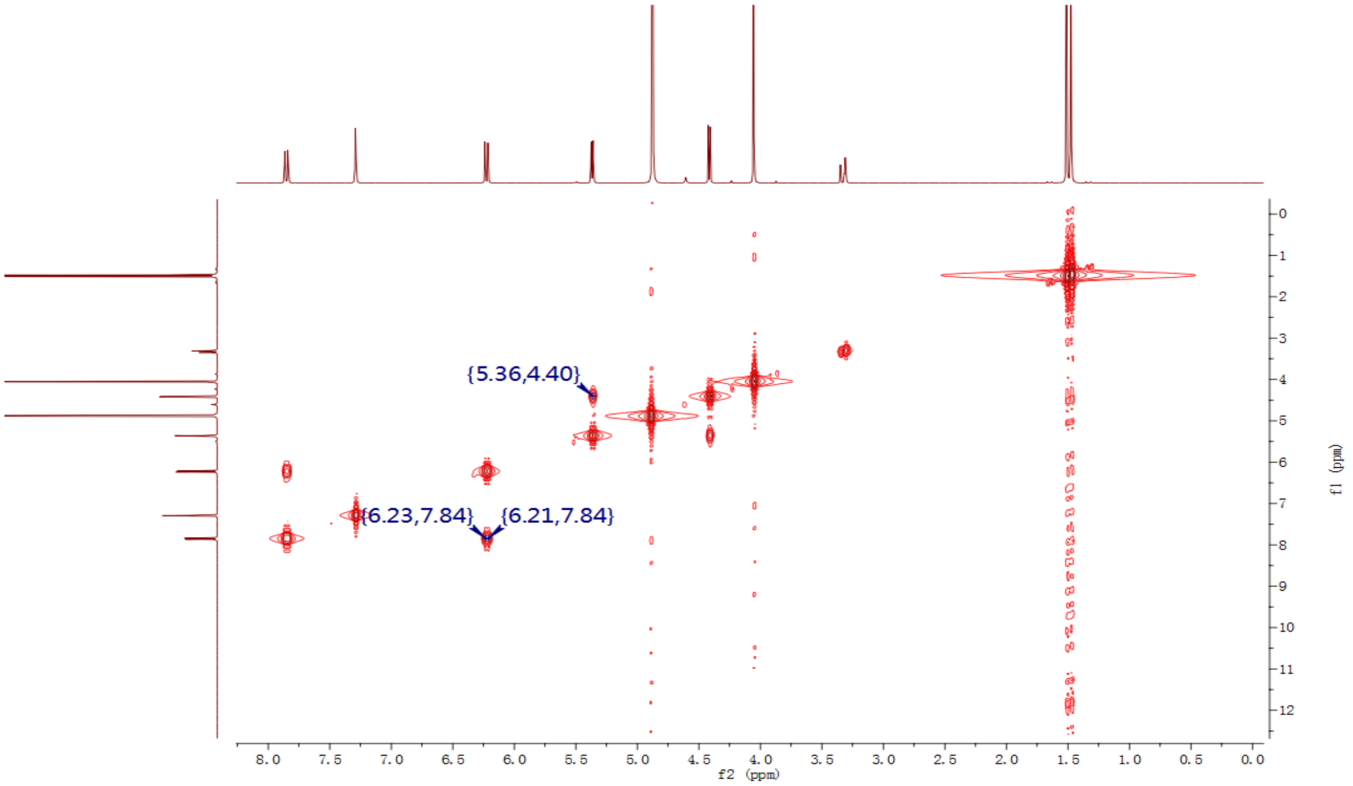

Figure S36 The NOE of Compound **3** in CD_3OD.

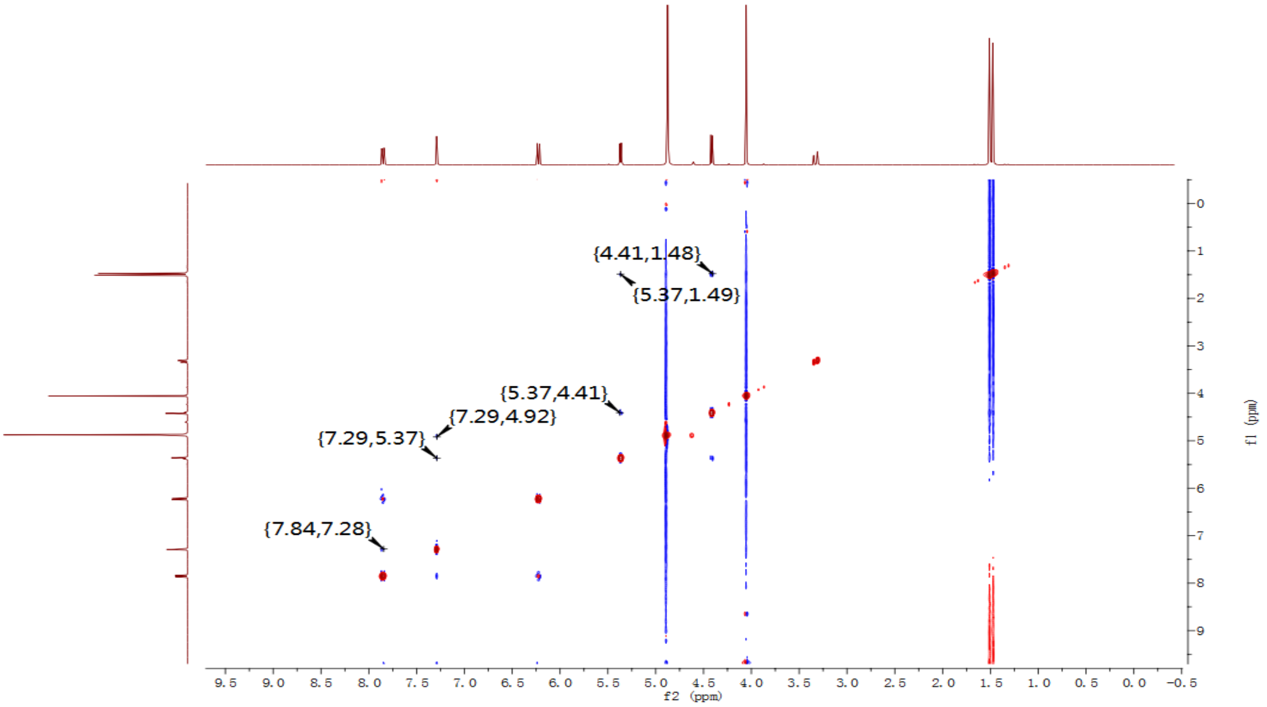

Figure S37 The crystal packing of Compound **3**

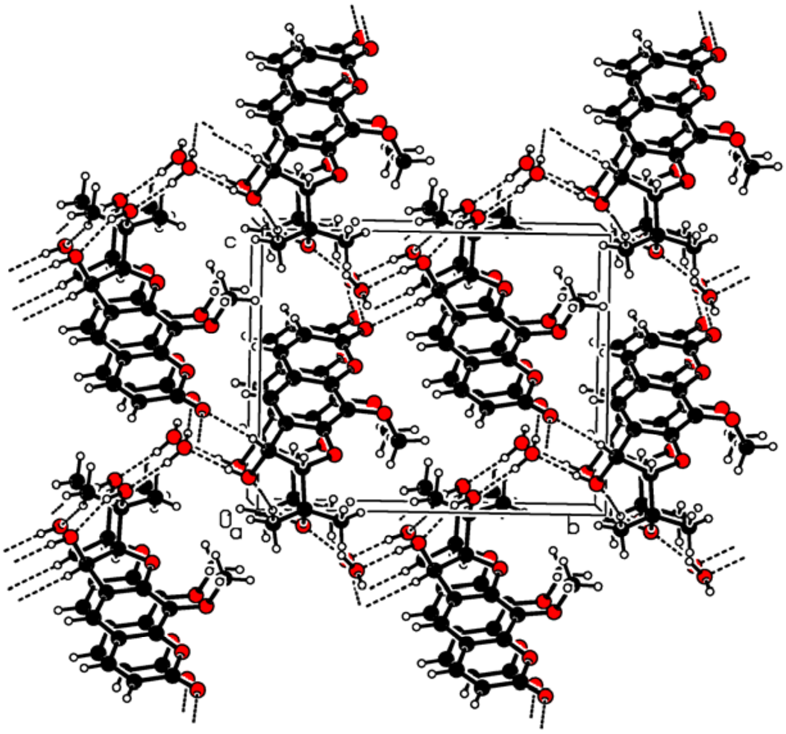

Figure S38 ^{1}H-NMR of Compound **4** in CD_3OD, 400 MHz

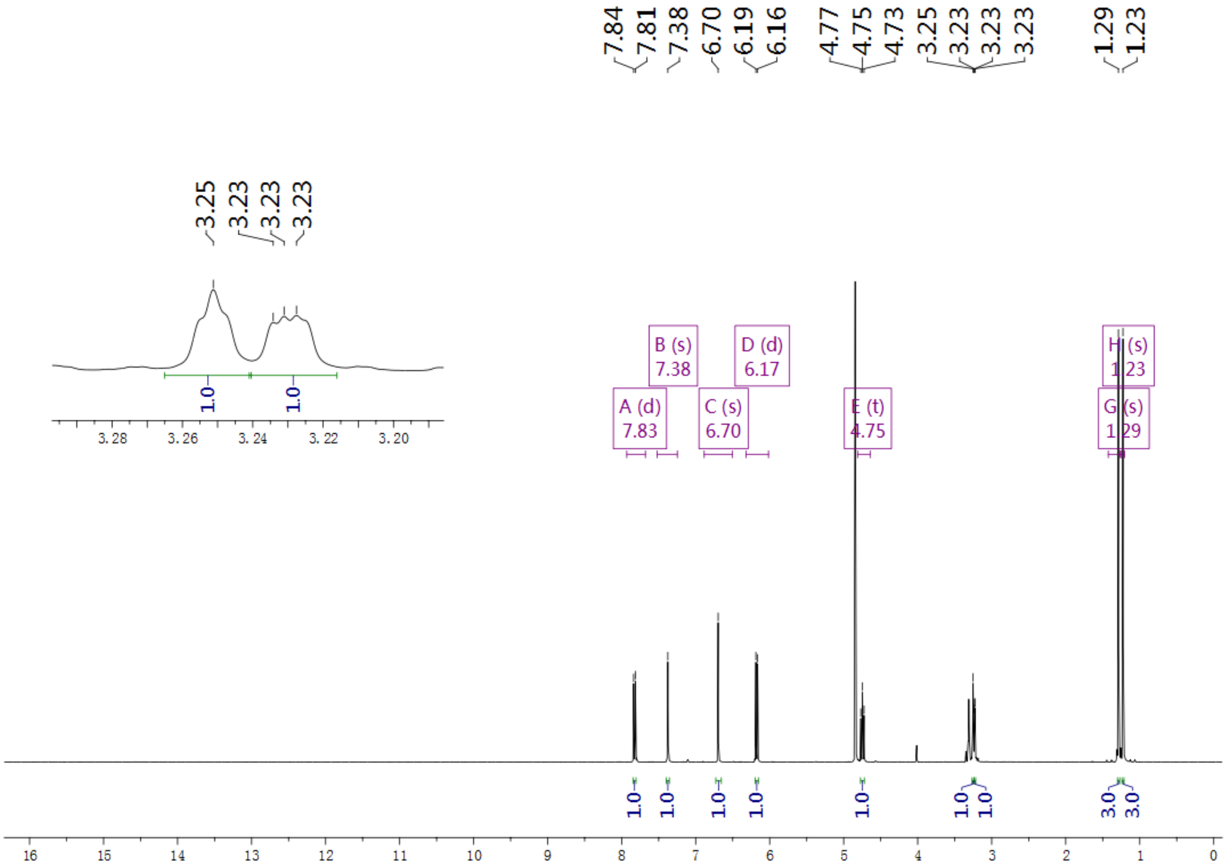

Figure S39 ^{13}C-NMR and DEPT of Compound **4** in CD_3OD, 100 MHz

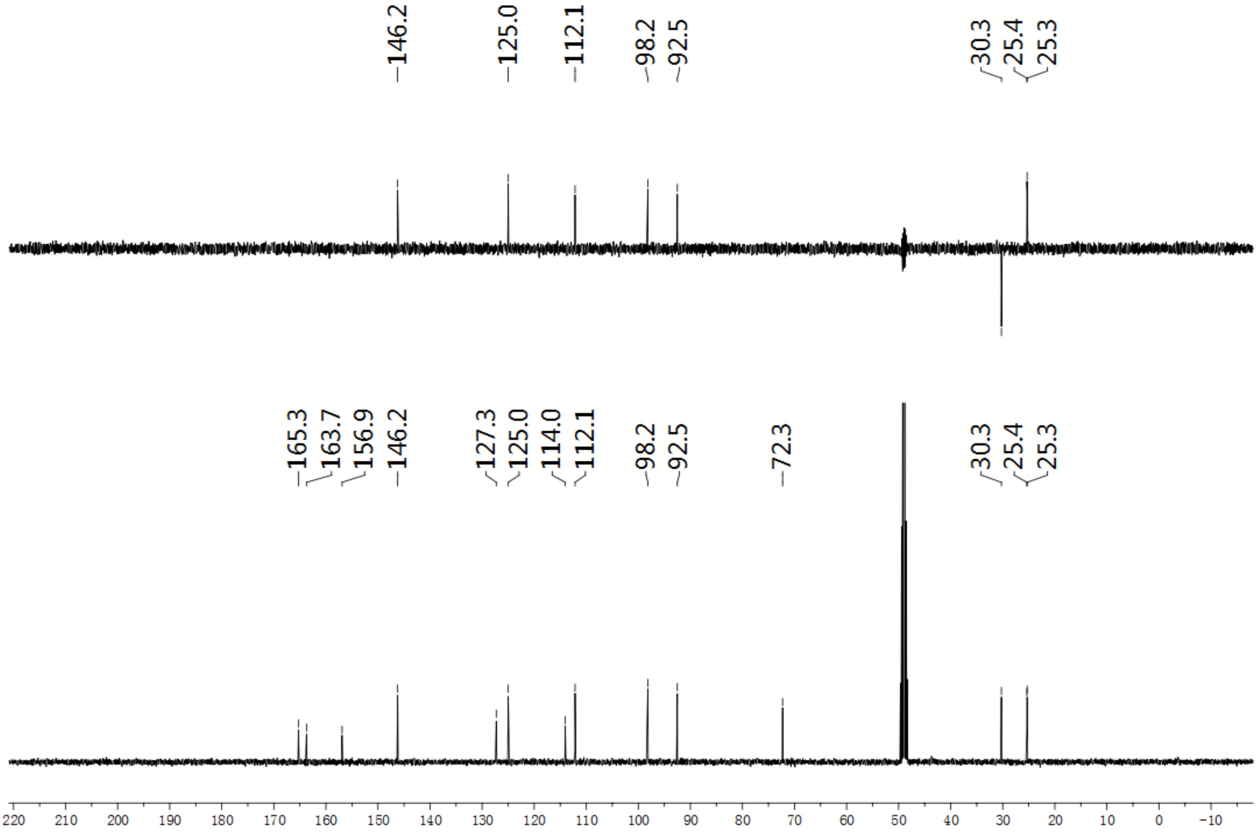

Figure S40 ORTEP View and crystal packing of Compound **4**

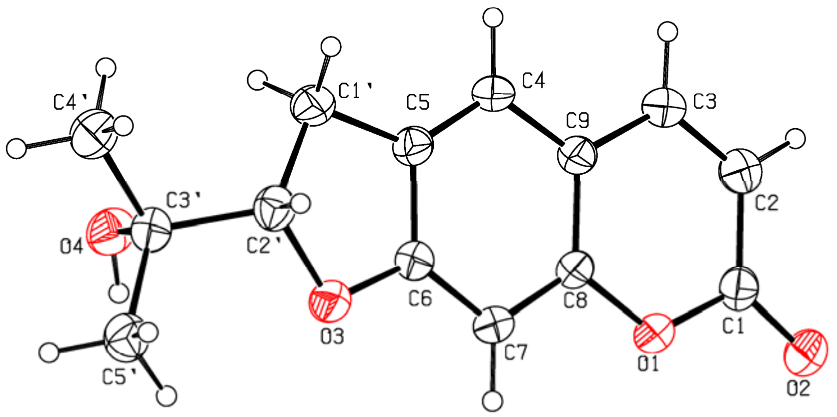

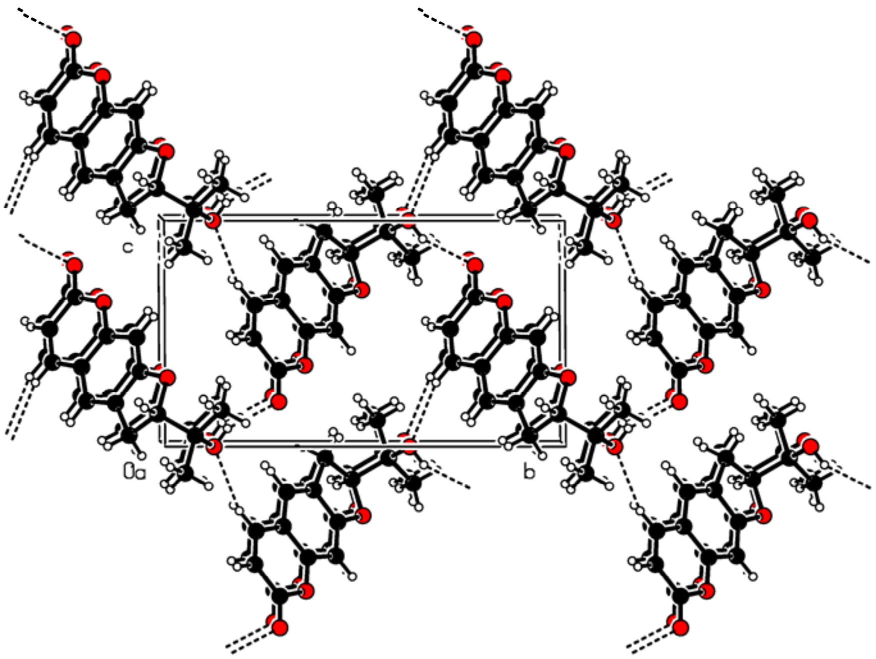

(CCDC No. 981340)

Table 1. Crystal data and structure refinement for Compound **4**

Crystal data and structure refinement for Compound **4** (CCDC No. 981340)	
Empirical formula	$C_{14} H_{14} O_4$
Formula weight	246.25
Temperature	298(2) K
Wavelength	1.54178 Å
Crystal system	Monoclinic
Space group	P2(1)
Unit cell dimensions	a = 5.72240(10) Å, = 90°
	b = 13.8219(3) Å, = 100.5080(10)°
	c = 7.8652(2) Å, = 90°
Volume	611.66(2) $Å^3$
Z	2
Density (calculated)	1.337 Mg/m^3
Absorption coefficient	0.812 mm^{-1}
F(000)	260
Crystal size	0.30 × 0.20 × 0.20 mm^3
Theta range for data collection	5.72 to 71.23°
Index ranges	–6<=h<=5, –16<=k<=16, –9<=l<=9
Reflections collected	15379
Independent reflections	2265 [R(int) = 0.0386]
Completeness to theta = 71.23°	96.6 %
Absorption correction	None
Max. and min. transmission	0.8544 and 0.7927
Refinement method	Full-matrix least-squares on F^2
Data / restraints / parameters	2265 / 1 / 167
Goodness-of-fit on F^2	1.051
Final R indices [I>2sigma(I)]	R1 = 0.0290, wR2 = 0.0809
R indices (all data)	R1 = 0.0290, wR2 = 0.0809
Absolute structure parameter	0.06(14)
Extinction coefficient	0.073(4)
Largest diff. peak and hole	0.181 and -0.144 e. $Å^{-3}$

Figure S41. ^{1}H-NMR of Compound **5** in CD_3Cl, 400 MHz

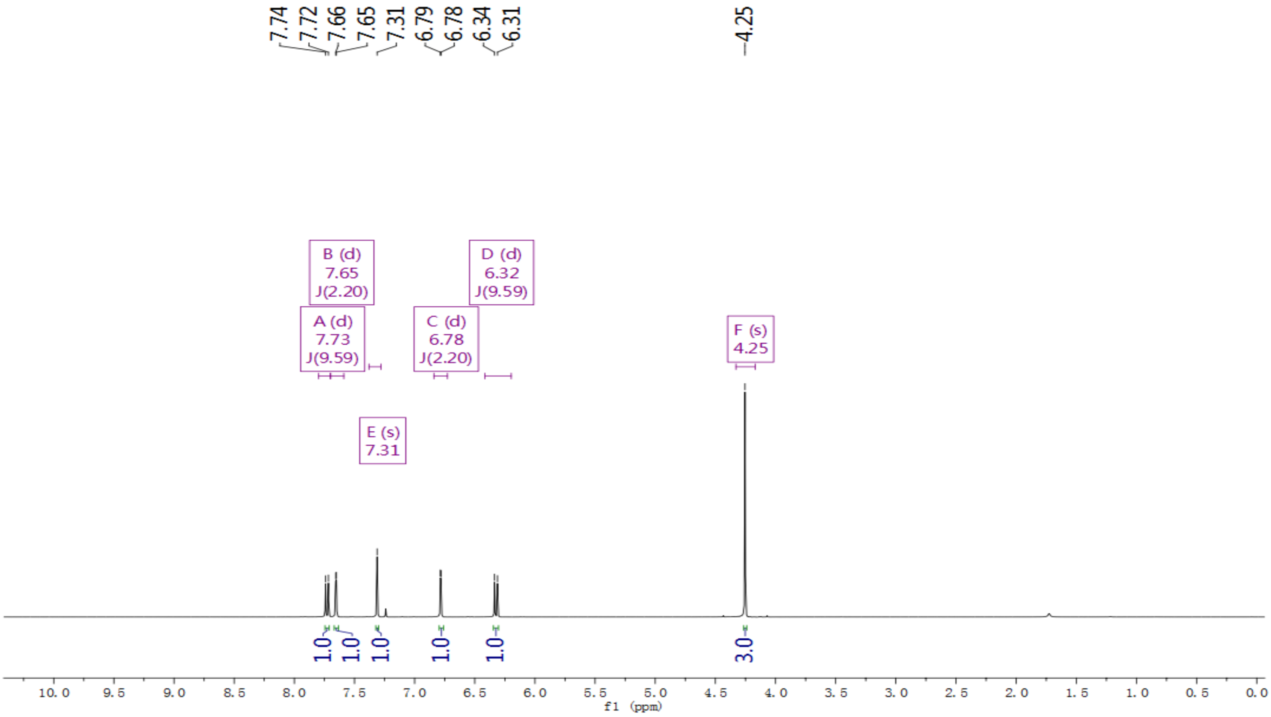

Figure S42. ^{13}C-NMR of Compound **5** in CD_3Cl, 100 MHz

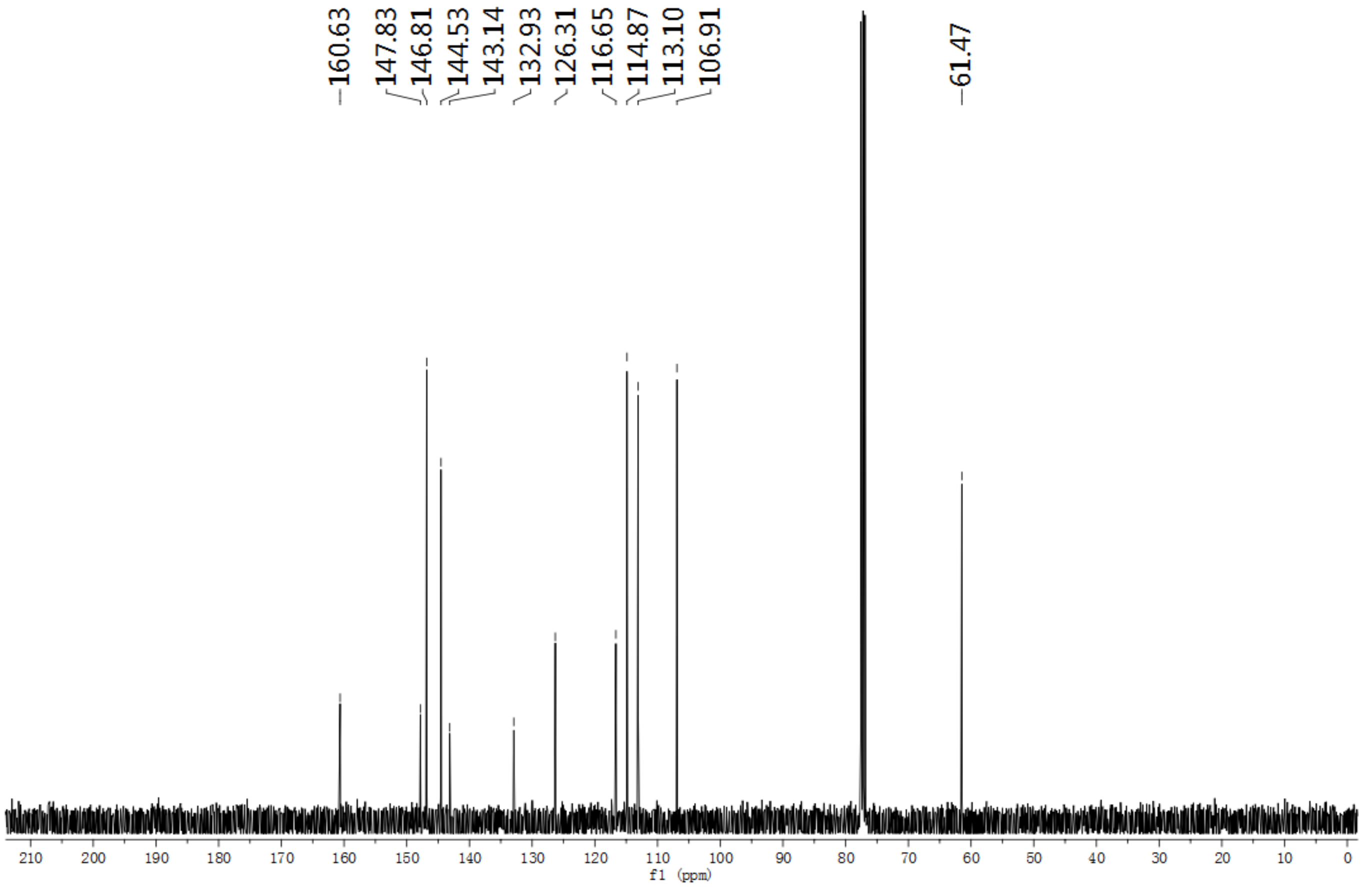

Figure S43 ORTEP View and crystal packing of Compound **5**

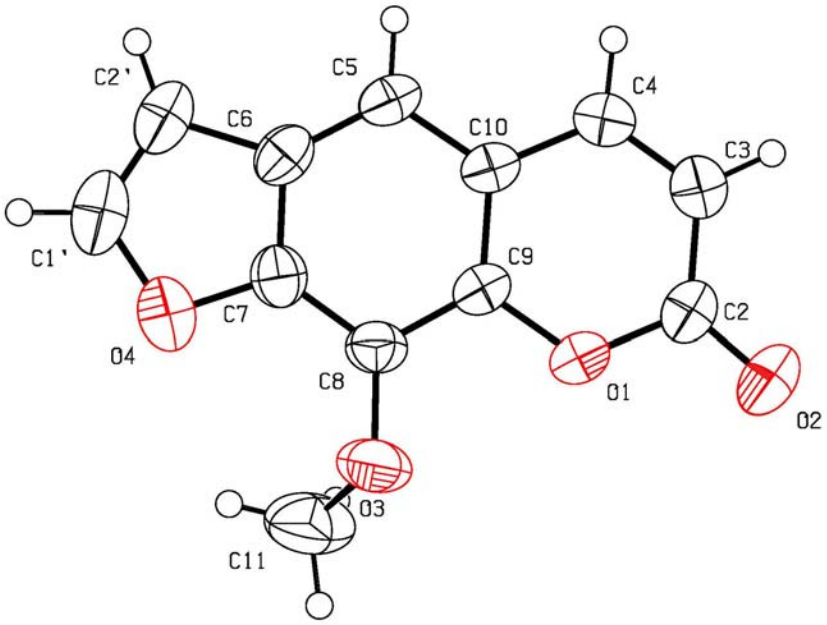

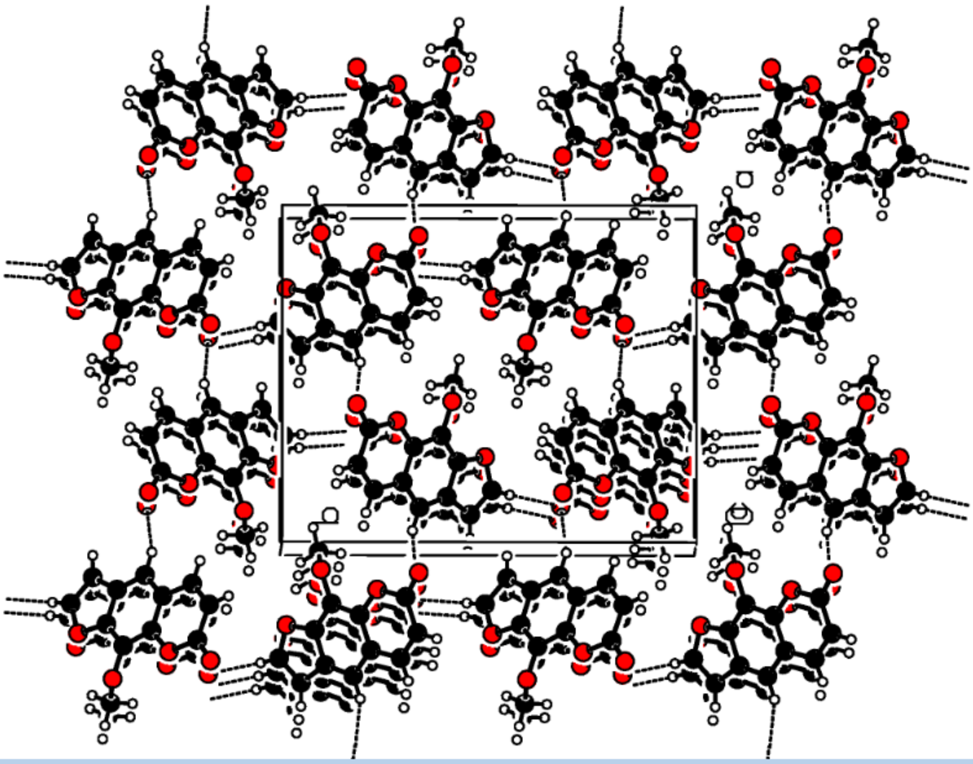

(CCDC No. 981341)

Table 2. Crystal data and structure refinement for Compound **5**

Crystal data and structure refinement for Compound **5** (CCDC No. 981341)	
Empirical formula	$C_{12} H_8 O_4$
Formula weight	246.25
Temperature	298(2) K
Wavelength	0.71073 Å
Crystal system	Orthorhombic
Space group	Pna2(1)
Unit cell dimensions	a = 12.883(2) Å, = 90°
	b = 15.779(2) Å, = 90°
	c = 4.8658(8) Å, = 90°
Volume	989.1(3) Å^3
Z	4
Density (calculated)	1.452 Mg/m^3
Absorption coefficient	0.110 mm^{-1}
F(000)	448
Crystal size	0.15 × 0.12 ×0.10 mm^3
Theta range for data collection	2.04 to 29.98°
Index ranges	–18<=h<=15, –22<=k<=22, –6<=l<=6
Reflections collected	9922
Independent reflections	2863 [R(int) = 0.0749]
Completeness to theta = 71.23°	100.0 %
Absorption correction	None
Max. and min. transmission	0.9890 and 0.9836
Refinement method	Full-matrix least-squares on F^2
Data / restraints / parameters	2863 / 1 / 146
Goodness-of-fit on F^2	1.007
Final R indices [I>2sigma(I)]	R1 = 0.0486, wR2 = 0.1265
R indices (all data)	R1 = 0.0694, wR2 = 0.1467
Largest diff. peak and hole	0.234 and -0.267 e.Å^{-3}

Figure S44. ^{1}H-NMR of Compound **6** in CD_3Cl, 400 MHz

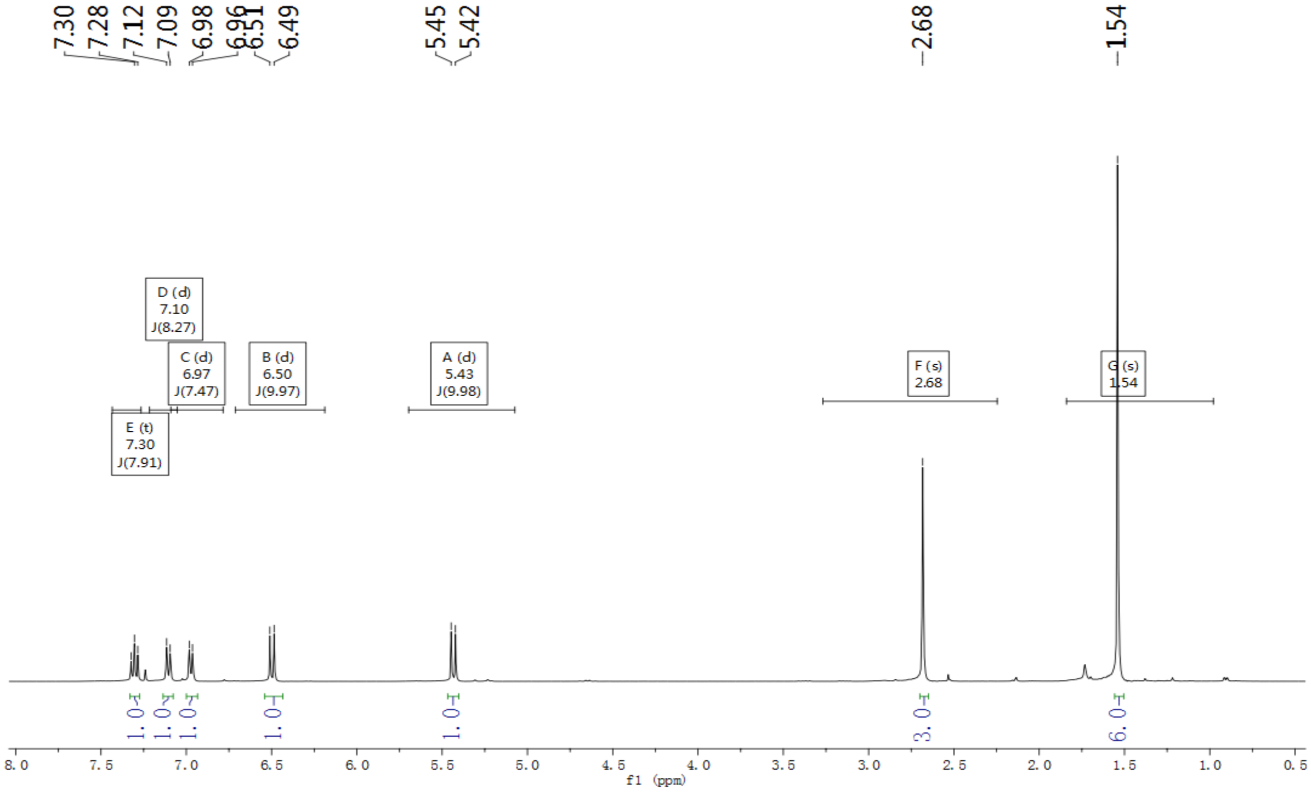

Figure S45. ^{13}C-NMR of Compound **6** in CD_3Cl, 100 MHz

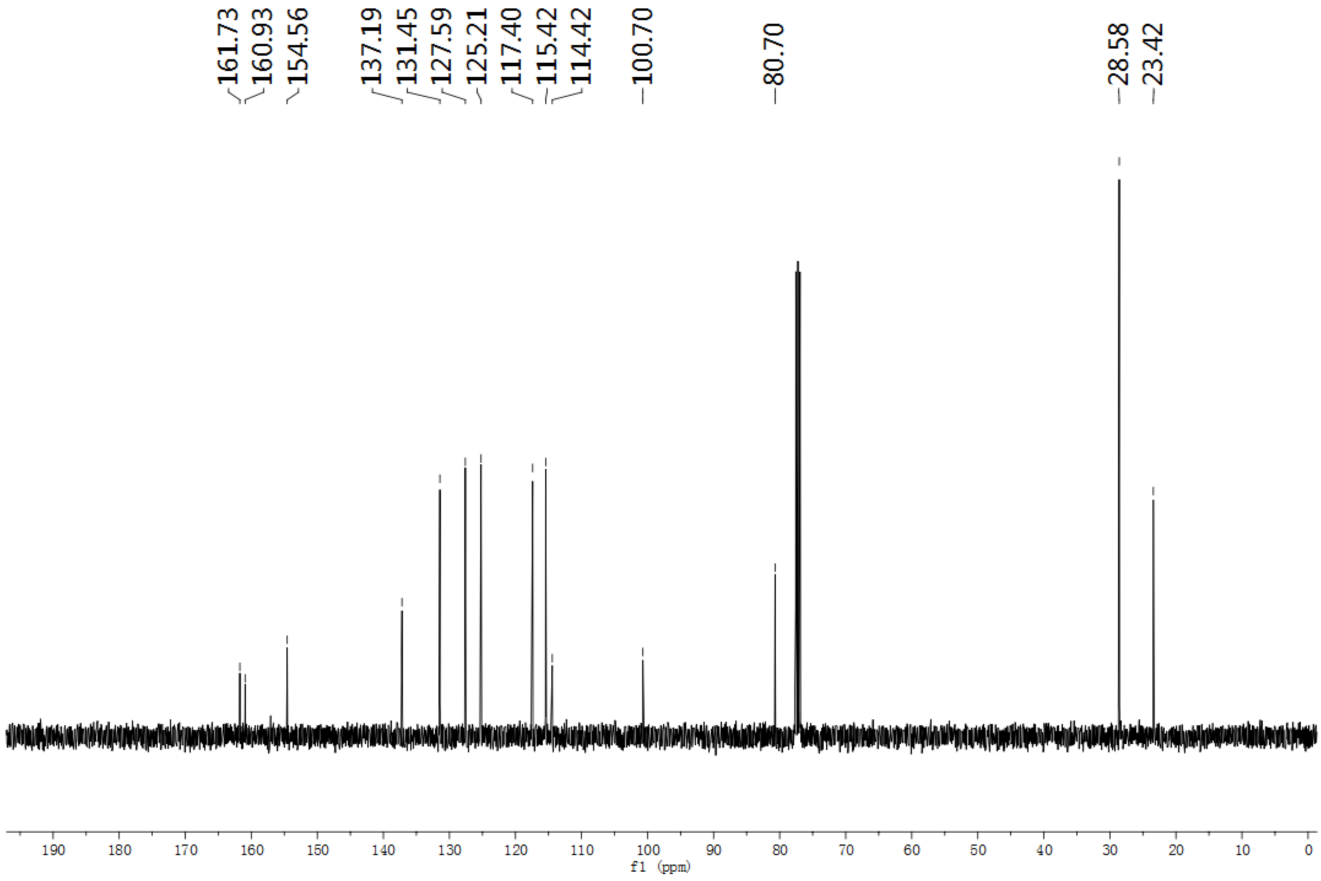

checkCIF/PLATON report

You have not supplied any structure factors. As a result the full set of tests cannot be run.

THIS REPORT IS FOR GUIDANCE ONLY. IF USED AS PART OF A REVIEW PROCEDURE FOR PUBLICATION, IT SHOULD NOT REPLACE THE EXPERTISE OF AN EXPERIENCED CRYSTALLOGRAPHIC REFEREE.

No syntax errors found.   CIF dictionary   Interpreting this report

Datablock: g__140425f_work_cu_140425f_0m

```
Bond precision:  C-C = 0.0027 A          Wavelength=1.54178

Cell:          a=7.2247(2)       b=7.6916(2)      c=16.5807(3)
               alpha=79.102(1)   beta=82.835(1)   gamma=89.821(1)
Temperature:   298 K

                Calculated                 Reported
Volume          897.50(4)                  897.50(4)
Space group     P -1                       P-1
Hall group      -P 1                       ?
Moiety formula  C20 H26 O4                 ?
Sum formula     C20 H26 O4                 C20 H26 O4
Mr              330.41                     330.41
Dx,g cm-3       1.223                      1.223
Z               2                          2
Mu (mm-1)       0.675                      0.675
F000            356.0                      356.0
F000’           357.08
h,k,lmax        8,9,19                     8,8,19
Nref            3044                       2917
Tmin,Tmax       0.922,0.935                0.877,0.936
Tmin’           0.874

Correction method= NONE

Data completeness= 0.958          Theta(max)= 64.960

R(reflections)= 0.0482( 2568)     wR2(reflections)= 0.1336( 2917)

S = 1.028                  Npar= 363
```

The following ALERTS were generated. Each ALERT has the format
test-name_ALERT_alert-type_alert-level.
Click on the hyperlinks for more details of the test.

Alert level B

```
PLAT029_ALERT_3_B _diffrn_measured_fraction_theta_full Low .......      0.958 Note
```

Alert level C

```
ABSTY03_ALERT_1_C  The _exptl_absorpt_correction_type has been given as none.
            However values have been given for Tmin and Tmax. Remove
            these if an absorption correction has not been applied.
  From the CIF: _exptl_absorpt_correction_T_min   0.877
  From the CIF: _exptl_absorpt_correction_T_max   0.936
REFNR01_ALERT_3_C  Ratio of reflections to parameters is < 10 for a
            centrosymmetric structure
            sine(theta)/lambda                0.5876
            Proportion of unique data used    1.0000
            Ratio reflections to parameters   8.0358
THETM01_ALERT_3_C  The value of sine(theta_max)/wavelength is less than 0.590
            Calculated sin(theta_max)/wavelength =    0.5876
PLAT088_ALERT_3_C Poor Data / Parameter Ratio ....................       8.39 Note
PLAT220_ALERT_2_C Large Non-Solvent  C     Ueq(max)/Ueq(min) Range        3.8 Ratio
PLAT222_ALERT_3_C Large Non-Solvent    H    Uiso(max)/Uiso(min) ..        4.7 Ratio
PLAT234_ALERT_4_C Large Hirshfeld Difference O1'    --  C9      ..       0.16 Ang.
PLAT250_ALERT_2_C Large U3/U1 Ratio for Average U(i,j) Tensor ....        2.3 Note
```

Alert level G

```
PLAT002_ALERT_2_G Number of Distance or Angle Restraints on AtSite         34 Note
PLAT005_ALERT_5_G No _iucr_refine_instructions_details  in the CIF     Please Do !
PLAT007_ALERT_5_G Number of Unrefined Donor-H Atoms ..............          4 Report
PLAT093_ALERT_1_G No su's on H-positions, refinement reported as .      mixed
PLAT154_ALERT_1_G The su's on the Cell Angles are Equal ..........    0.00100 Degree
PLAT230_ALERT_2_G Hirshfeld Test Diff for    C11    --  C19     ..        6.8 su
PLAT301_ALERT_3_G Main Residue  Disorder ............ Percentage =         71 Note
PLAT779_ALERT_4_G Suspect or Irrelevant (Bond) Angle in CIF .... #         16 Check
              O1   -C9   -O1'     1.555   1.555   1.555              2.50 Deg.
PLAT779_ALERT_4_G Suspect or Irrelevant (Bond) Angle in CIF .... #         24 Check
              C4   -C10  -C4'     1.555   1.555   1.555             20.70 Deg.
PLAT793_ALERT_4_G The Model has Chirality at C11     .............          R Verify
PLAT793_ALERT_4_G The Model has Chirality at C12     .............          R Verify
PLAT793_ALERT_4_G The Model has Chirality at C11'    .............          S Verify
PLAT793_ALERT_4_G The Model has Chirality at C12'    .............          S Verify
PLAT811_ALERT_5_G No ADDSYM Analysis: Too Many Excluded Atoms ....          ! Info
PLAT860_ALERT_3_G Number of Least-Squares Restraints .............         40 Note
PLAT899_ALERT_4_G SHELXL97   is Deprecated and Succeeded by SHELXL       2014 Note
```

```
   0 ALERT level A = Most likely a serious problem - resolve or explain
   1 ALERT level B = A potentially serious problem, consider carefully
   8 ALERT level C = Check. Ensure it is not caused by an omission or oversight
  16 ALERT level G = General information/check it is not something unexpected

   3 ALERT type 1 CIF construction/syntax error, inconsistent or missing data
   4 ALERT type 2 Indicator that the structure model may be wrong or deficient
   7 ALERT type 3 Indicator that the structure quality may be low
   8 ALERT type 4 Improvement, methodology, query or suggestion
   3 ALERT type 5 Informative message, check
```

It is advisable to attempt to resolve as many as possible of the alerts in all categories. Often the minor alerts point to easily fixed oversights, errors and omissions in your CIF or refinement strategy, so attention to these fine details can be worthwhile. In order to resolve some of the more serious problems it may be necessary to carry out additional measurements or structure refinements. However, the purpose of your study may justify the reported deviations and the more serious of these should normally be commented upon in the discussion or experimental section of a paper or in the "special_details" fields of the CIF. checkCIF was carefully designed to identify outliers and unusual parameters, but every test has its limitations and alerts that are not important in a particular case may appear. Conversely, the absence of alerts does not guarantee there are no aspects of the results needing attention. It is up to the individual to critically assess their own results and, if necessary, seek expert advice.

Publication of your CIF in IUCr journals

A basic structural check has been run on your CIF. These basic checks will be run on all CIFs submitted for publication in IUCr journals (*Acta Crystallographica*, *Journal of Applied Crystallography*, *Journal of Synchrotron Radiation*); however, if you intend to submit to *Acta Crystallographica Section C* or *E*, you should make sure that full publication checks are run on the final version of your CIF prior to submission.

Publication of your CIF in other journals

Please refer to the *Notes for Authors* of the relevant journal for any special instructions relating to CIF submission.

PLATON version of 20/08/2014; check.def file version of 18/08/2014

Datablock g__140425f_work_cu_140425f_0m - ellipsoid plot

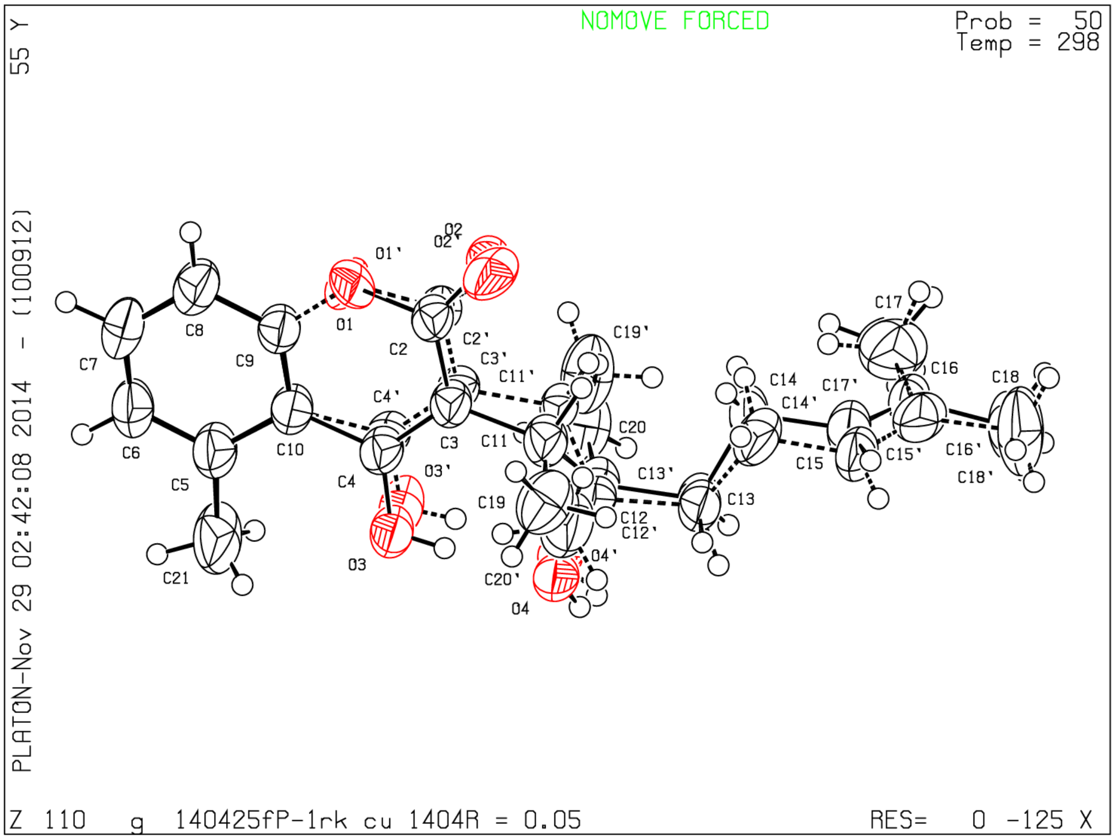

checkCIF/PLATON report

You have not supplied any structure factors. As a result the full set of tests cannot be run.

THIS REPORT IS FOR GUIDANCE ONLY. IF USED AS PART OF A REVIEW PROCEDURE FOR PUBLICATION, IT SHOULD NOT REPLACE THE EXPERTISE OF AN EXPERIENCED CRYSTALLOGRAPHIC REFEREE.

No syntax errors found. CIF dictionary Interpreting this report

Datablock: e__131228a_work_cu_131228a_0m

```
Bond precision:  C-C = 0.0068 A          Wavelength=1.54178

Cell:          a=7.2264(2)        b=7.6852(2)      c=16.5860(3)
               alpha=100.897(1)   beta=96.983(1)   gamma=90.124(1)
Temperature:   296 K

                Calculated                 Reported
Volume          897.51(4)                  897.51(4)
Space group     P 1                        P1
Hall group      P 1                        ?
Moiety formula  C20 H26 O4                 ?
Sum formula     C20 H26 O4                 C20 H26 O4
Mr              330.41                     330.41
Dx,g cm-3       1.223                      1.223
Z               2                          2
Mu (mm-1)       0.675                      0.675
F000            356.0                      356.0
F000'           357.08
h,k,lmax        8,9,19                     8,9,19
Nref            6482[ 3241]                5691
Tmin,Tmax       0.922,0.935                0.877,0.936
Tmin'           0.874

Correction method= NONE

Data completeness= 1.76/0.88      Theta(max)= 67.460

R(reflections)= 0.0681( 5462)     wR2(reflections)= 0.1932( 5691)

S = 1.120                  Npar= 446
```

The following ALERTS were generated. Each ALERT has the format
test-name_ALERT_alert-type_alert-level.
Click on the hyperlinks for more details of the test.

Alert level B

```
PLAT029_ALERT_3_B _diffrn_measured_fraction_theta_full Low .......      0.950 Note
PLAT230_ALERT_2_B Hirshfeld Test Diff for    C15    --  C16     ..        7.2 su
PLAT416_ALERT_2_B Short Intra D-H..H-D       H3A1   ..  H4A     ..       1.74 Ang.
```

Alert level C

```
ABSTY03_ALERT_1_C  The _exptl_absorpt_correction_type has been given as none.
            However values have been given for Tmin and Tmax. Remove
            these if an absorption correction has not been applied.
  From the CIF: _exptl_absorpt_correction_T_min   0.877
  From the CIF: _exptl_absorpt_correction_T_max   0.936
PLAT089_ALERT_3_C Poor Data / Parameter Ratio (Zmax < 18) ........       7.27 Note
PLAT111_ALERT_2_C ADDSYM Detects (Pseudo) Centre of Symmetry .....         95 %Fit
PLAT113_ALERT_2_C ADDSYM Suggests Possible Pseudo/New Space group.        P-1 Check
PLAT213_ALERT_2_C Atom O3A             has ADP max/min Ratio .....        3.3 prolat
PLAT213_ALERT_2_C Atom C21A            has ADP max/min Ratio .....        3.2 prolat
PLAT230_ALERT_2_C Hirshfeld Test Diff for    C15A   --  C16A    ..        5.2 su
PLAT234_ALERT_4_C Large Hirshfeld Difference C12A   --  C20A    ..       0.18 Ang.
PLAT241_ALERT_2_C High      Ueq as Compared to Neighbors for .....        C14 Check
PLAT241_ALERT_2_C High      Ueq as Compared to Neighbors for .....       C14A Check
PLAT242_ALERT_2_C Low       Ueq as Compared to Neighbors for .....        C11 Check
PLAT242_ALERT_2_C Low       Ueq as Compared to Neighbors for .....        C13 Check
PLAT242_ALERT_2_C Low       Ueq as Compared to Neighbors for .....        C16 Check
PLAT242_ALERT_2_C Low       Ueq as Compared to Neighbors for .....        C5A Check
PLAT242_ALERT_2_C Low       Ueq as Compared to Neighbors for .....       C11A Check
PLAT242_ALERT_2_C Low       Ueq as Compared to Neighbors for .....       C13A Check
PLAT242_ALERT_2_C Low       Ueq as Compared to Neighbors for .....       C16A Check
PLAT250_ALERT_2_C Large U3/U1 Ratio for Average U(i,j) Tensor ....        2.7 Note
PLAT250_ALERT_2_C Large U3/U1 Ratio for Average U(i,j) Tensor ....        3.0 Note
PLAT340_ALERT_3_C Low Bond Precision on  C-C Bonds ...............     0.0068 Ang.
```

Alert level G

```
PLAT002_ALERT_2_G Number of Distance or Angle Restraints on AtSite          4 Note
PLAT005_ALERT_5_G No _iucr_refine_instructions_details  in the CIF     Please Do !
PLAT007_ALERT_5_G Number of Unrefined Donor-H Atoms ..............          4 Report
PLAT032_ALERT_4_G Std. Uncertainty on Flack Parameter Value High .      0.300 Report
PLAT093_ALERT_1_G No su's on H-positions, refinement reported as .      mixed
PLAT154_ALERT_1_G The su's on the Cell Angles are Equal ..........    0.00100 Degree
PLAT720_ALERT_4_G Number of Unusual/Non-Standard Labels ..........          1 Note
PLAT791_ALERT_4_G The Model has Chirality at C11      .............         R Verify
PLAT791_ALERT_4_G The Model has Chirality at C11A     .............         R Verify
PLAT791_ALERT_4_G The Model has Chirality at C12      .............         R Verify
PLAT791_ALERT_4_G The Model has Chirality at C12A     .............         R Verify
PLAT860_ALERT_3_G Number of Least-Squares Restraints .............          5 Note
PLAT899_ALERT_4_G SHELXL97   is Deprecated and Succeeded by SHELXL       2014 Note
```

---

   0 **ALERT level A** = Most likely a serious problem - resolve or explain
   3 **ALERT level B** = A potentially serious problem, consider carefully
  20 **ALERT level C** = Check. Ensure it is not caused by an omission or oversight
  13 **ALERT level G** = General information/check it is not something unexpected

```
   3 ALERT type 1 CIF construction/syntax error, inconsistent or missing data
  19 ALERT type 2 Indicator that the structure model may be wrong or deficient
   4 ALERT type 3 Indicator that the structure quality may be low
   8 ALERT type 4 Improvement, methodology, query or suggestion
   2 ALERT type 5 Informative message, check
```

It is advisable to attempt to resolve as many as possible of the alerts in all categories. Often the minor alerts point to easily fixed oversights, errors and omissions in your CIF or refinement strategy, so attention to these fine details can be worthwhile. In order to resolve some of the more serious problems it may be necessary to carry out additional measurements or structure refinements. However, the purpose of your study may justify the reported deviations and the more serious of these should normally be commented upon in the discussion or experimental section of a paper or in the "special_details" fields of the CIF. checkCIF was carefully designed to identify outliers and unusual parameters, but every test has its limitations and alerts that are not important in a particular case may appear. Conversely, the absence of alerts does not guarantee there are no aspects of the results needing attention. It is up to the individual to critically assess their own results and, if necessary, seek expert advice.

Publication of your CIF in IUCr journals

A basic structural check has been run on your CIF. These basic checks will be run on all CIFs submitted for publication in IUCr journals (*Acta Crystallographica*, *Journal of Applied Crystallography*, *Journal of Synchrotron Radiation*); however, if you intend to submit to *Acta Crystallographica Section C* or *E*, you should make sure that full publication checks are run on the final version of your CIF prior to submission.

Publication of your CIF in other journals

Please refer to the *Notes for Authors* of the relevant journal for any special instructions relating to CIF submission.

PLATON version of 20/08/2014; check.def file version of 18/08/2014

Datablock e__131228a_work_cu_131228a_0m - ellipsoid plot

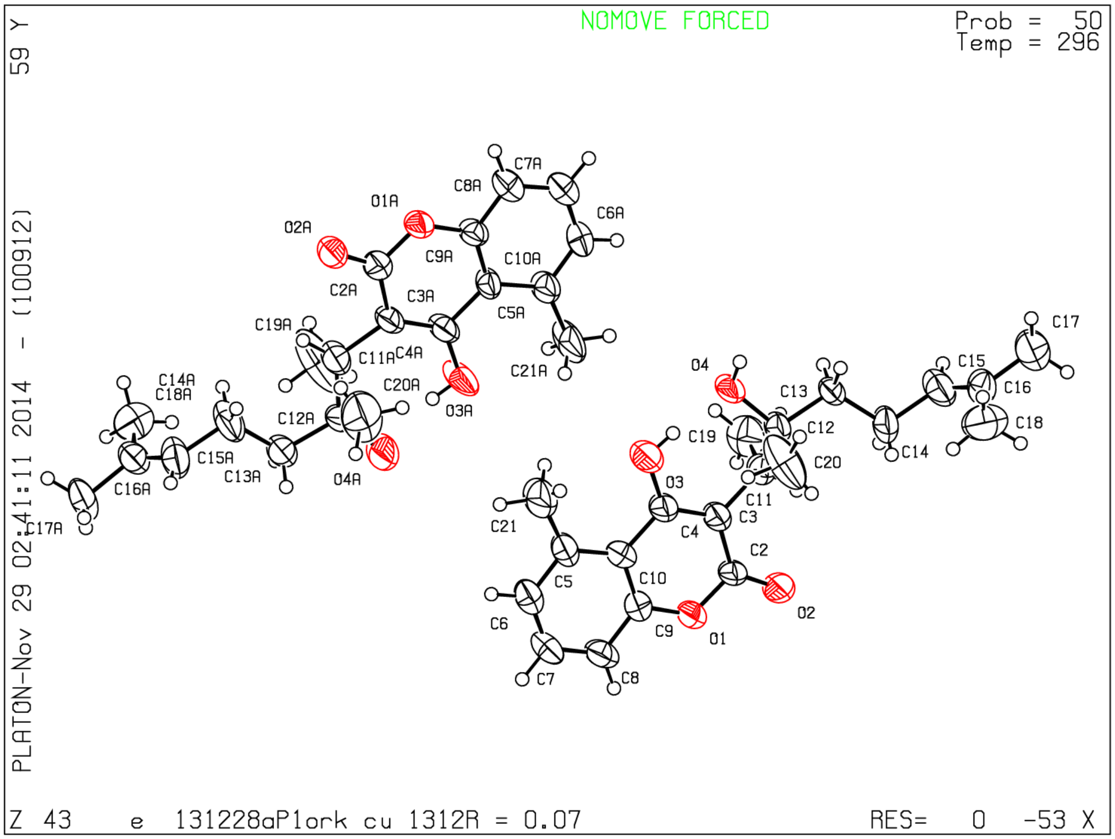

checkCIF/PLATON report

You have not supplied any structure factors. As a result the full set of tests cannot be run.

THIS REPORT IS FOR GUIDANCE ONLY. IF USED AS PART OF A REVIEW PROCEDURE FOR PUBLICATION, IT SHOULD NOT REPLACE THE EXPERTISE OF AN EXPERIENCED CRYSTALLOGRAPHIC REFEREE.

No syntax errors found. CIF dictionary Interpreting this report

Datablock: g__140323e_work_cu_140323e_0m

```
Bond precision:  C-C = 0.0017 A          Wavelength=1.54178

Cell:            a=5.6562(1)     b=13.7286(2)       c=21.7344(4)
                 alpha=90        beta=91.225(1)     gamma=90
Temperature:     298 K

                Calculated                 Reported
Volume          1687.33(5)                 1687.33(5)
Space group     P 21/c                     P2(1)/c
Hall group      -P 2ybc                    ?
Moiety formula  C20 H22 O5                 ?
Sum formula     C20 H22 O5                 C20 H22 O5
Mr              342.38                     342.38
Dx,g cm-3       1.348                      1.348
Z               4                          4
Mu (mm-1)       0.790                      0.790
F000            728.0                      728.0
F000'           730.35
h,k,lmax        6,16,26                    6,16,26
Nref            3260                       3173
Tmin,Tmax       0.789,0.854                0.798,0.858
Tmin'           0.789

Correction method= NONE

Data completeness= 0.973          Theta(max)= 71.130

R(reflections)= 0.0352( 3095)     wR2(reflections)= 0.0954( 3173)

S = 1.044                   Npar= 233
```

The following ALERTS were generated. Each ALERT has the format
test-name_ALERT_alert-type_alert-level.
Click on the hyperlinks for more details of the test.

Alert level C

```
ABSTY03_ALERT_1_C  The _exptl_absorpt_correction_type has been given as none.
            However values have been given for Tmin and Tmax. Remove
            these if an absorption correction has not been applied.
  From the CIF: _exptl_absorpt_correction_T_min   0.798
  From the CIF: _exptl_absorpt_correction_T_max   0.858
PLAT029_ALERT_3_C _diffrn_measured_fraction_theta_full Low .......      0.973 Note
PLAT413_ALERT_2_C Short Inter XH3 .. XHn     H11A   ..  H11A    ..       2.10 Ang.
```

Alert level G

```
PLAT005_ALERT_5_G No _iucr_refine_instructions_details  in the CIF     Please Do !
PLAT007_ALERT_5_G Number of Unrefined Donor-H Atoms .............          2 Report
PLAT093_ALERT_1_G No su’s on H-positions, refinement reported as .      mixed
PLAT793_ALERT_4_G The Model has Chirality at C13     .............          S Verify
PLAT793_ALERT_4_G The Model has Chirality at C14     .............          S Verify
PLAT899_ALERT_4_G SHELXL97   is Deprecated and Succeeded by SHELXL       2014 Note
```

```
   0 ALERT level A = Most likely a serious problem - resolve or explain
   0 ALERT level B = A potentially serious problem, consider carefully
   3 ALERT level C = Check. Ensure it is not caused by an omission or oversight
   6 ALERT level G = General information/check it is not something unexpected

   2 ALERT type 1 CIF construction/syntax error, inconsistent or missing data
   1 ALERT type 2 Indicator that the structure model may be wrong or deficient
   1 ALERT type 3 Indicator that the structure quality may be low
   3 ALERT type 4 Improvement, methodology, query or suggestion
   2 ALERT type 5 Informative message, check
```

It is advisable to attempt to resolve as many as possible of the alerts in all categories. Often the minor alerts point to easily fixed oversights, errors and omissions in your CIF or refinement strategy, so attention to these fine details can be worthwhile. In order to resolve some of the more serious problems it may be necessary to carry out additional measurements or structure refinements. However, the purpose of your study may justify the reported deviations and the more serious of these should normally be commented upon in the discussion or experimental section of a paper or in the "special_details" fields of the CIF. checkCIF was carefully designed to identify outliers and unusual parameters, but every test has its limitations and alerts that are not important in a particular case may appear. Conversely, the absence of alerts does not guarantee there are no aspects of the results needing attention. It is up to the individual to critically assess their own results and, if necessary, seek expert advice.

Publication of your CIF in IUCr journals

A basic structural check has been run on your CIF. These basic checks will be run on all CIFs submitted for publication in IUCr journals (*Acta Crystallographica*, *Journal of Applied Crystallography*, *Journal of Synchrotron Radiation*); however, if you intend to submit to *Acta Crystallographica Section C* or *E*, you should make sure that full publication checks are run on the final version of your CIF prior to submission.

Publication of your CIF in other journals

Please refer to the *Notes for Authors* of the relevant journal for any special instructions relating to CIF submission.

PLATON version of 20/08/2014; check.def file version of 18/08/2014

Datablock g__140323e_work_cu_140323e_0m - ellipsoid plot

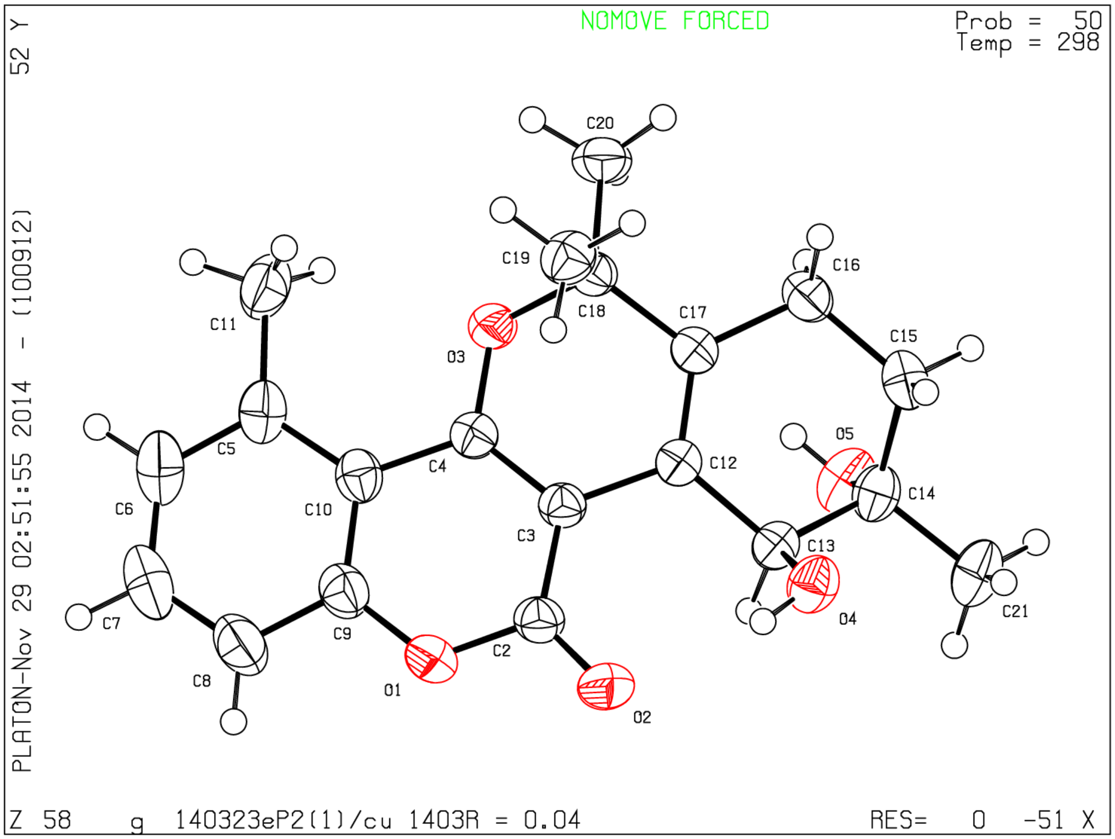

checkCIF/PLATON report

You have not supplied any structure factors. As a result the full set of tests cannot be run.

THIS REPORT IS FOR GUIDANCE ONLY. IF USED AS PART OF A REVIEW PROCEDURE FOR PUBLICATION, IT SHOULD NOT REPLACE THE EXPERTISE OF AN EXPERIENCED CRYSTALLOGRAPHIC REFEREE.

No syntax errors found.   CIF dictionary   Interpreting this report

Datablock: cu_zyh_ll1331a_0m

```
Bond precision:  C-C = 0.0021 A          Wavelength=1.54178

Cell:             a=13.7242(3)      b=22.2290(5)      c=5.5733(1)
                  alpha=90          beta=90           gamma=90
Temperature:      100 K

                Calculated                  Reported
Volume          1700.28(6)                  1700.28(6)
Space group     P 21 21 2                   P 21 21 2
Hall group      P 2 2ab                     P 2 2ab
Moiety formula  C20 H22 O5                  C20 H22 O5
Sum formula     C20 H22 O5                  C20 H22 O5
Mr              342.38                      342.38
Dx,g cm-3       1.337                       1.337
Z               4                           4
Mu (mm-1)       0.784                       0.784
F000            728.0                       728.0
F000’           730.35
h,k,lmax        16,26,6                     16,26,6
Nref            3187[ 1873]                 2998
Tmin,Tmax       0.844,0.917                 0.367,0.919
Tmin’           0.285

Correction method= MULTI-SCAN

Data completeness= 1.60/0.94      Theta(max)= 69.260

R(reflections)= 0.0376( 2969)     wR2(reflections)= 0.1153( 2998)

S = 1.112                 Npar= 232
```

The following ALERTS were generated. Each ALERT has the format
test-name_ALERT_alert-type_alert-level.
Click on the hyperlinks for more details of the test.

Alert level B

```
CRYSS02_ALERT_3_B  The value of _exptl_crystal_size_max is > 1.0
   Maximum crystal size given =   1.600
PLAT029_ALERT_3_B _diffrn_measured_fraction_theta_full Low .......      0.954 Note
```

Alert level G

```
PLAT005_ALERT_5_G No _iucr_refine_instructions_details  in the CIF     Please Do !
PLAT007_ALERT_5_G Number of Unrefined Donor-H Atoms .............          2 Report
PLAT063_ALERT_4_G Crystal Size Likely too Large for Beam Size ....       1.60 mm
PLAT791_ALERT_4_G The Model has Chirality at C13     .............          S Verify
PLAT791_ALERT_4_G The Model has Chirality at C14     .............          S Verify
PLAT899_ALERT_4_G SHELXL97   is Deprecated and Succeeded by SHELXL       2014 Note
```

```
   0 ALERT level A = Most likely a serious problem - resolve or explain
   2 ALERT level B = A potentially serious problem, consider carefully
   0 ALERT level C = Check. Ensure it is not caused by an omission or oversight
   6 ALERT level G = General information/check it is not something unexpected

   0 ALERT type 1 CIF construction/syntax error, inconsistent or missing data
   0 ALERT type 2 Indicator that the structure model may be wrong or deficient
   2 ALERT type 3 Indicator that the structure quality may be low
   4 ALERT type 4 Improvement, methodology, query or suggestion
   2 ALERT type 5 Informative message, check
```

It is advisable to attempt to resolve as many as possible of the alerts in all categories. Often the minor alerts point to easily fixed oversights, errors and omissions in your CIF or refinement strategy, so attention to these fine details can be worthwhile. In order to resolve some of the more serious problems it may be necessary to carry out additional measurements or structure refinements. However, the purpose of your study may justify the reported deviations and the more serious of these should normally be commented upon in the discussion or experimental section of a paper or in the "special_details" fields of the CIF. checkCIF was carefully designed to identify outliers and unusual parameters, but every test has its limitations and alerts that are not important in a particular case may appear. Conversely, the absence of alerts does not guarantee there are no aspects of the results needing attention. It is up to the individual to critically assess their own results and, if necessary, seek expert advice.

Publication of your CIF in IUCr journals

A basic structural check has been run on your CIF. These basic checks will be run on all CIFs submitted for publication in IUCr journals (*Acta Crystallographica*, *Journal of Applied Crystallography*, *Journal of Synchrotron Radiation*); however, if you intend to submit to *Acta Crystallographica Section C* or *E*, you should make sure that full publication checks are run on the final version of your CIF prior to submission.

Publication of your CIF in other journals

Please refer to the *Notes for Authors* of the relevant journal for any special instructions relating to CIF submission.

PLATON version of 20/08/2014; check.def file version of 18/08/2014

Datablock cu_zyh_ll1331a_0m - ellipsoid plot

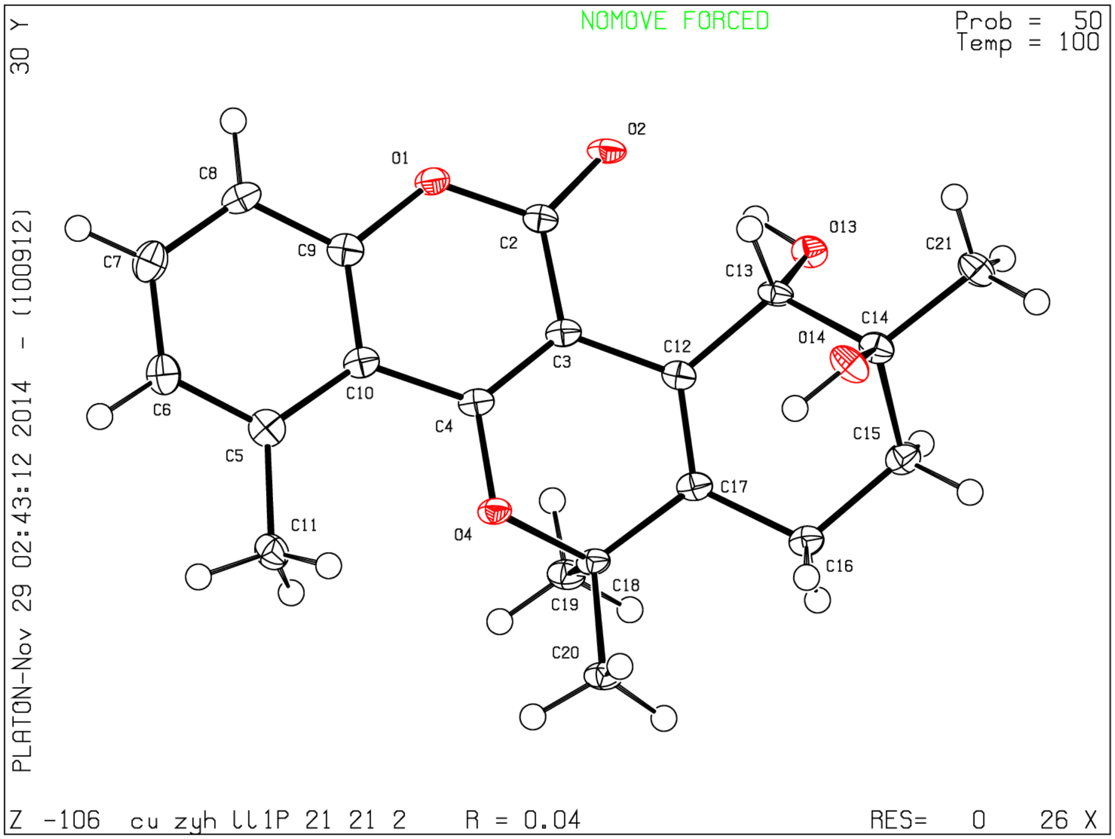

checkCIF/PLATON report

You have not supplied any structure factors. As a result the full set of tests cannot be run.

THIS REPORT IS FOR GUIDANCE ONLY. IF USED AS PART OF A REVIEW PROCEDURE FOR PUBLICATION, IT SHOULD NOT REPLACE THE EXPERTISE OF AN EXPERIENCED CRYSTALLOGRAPHIC REFEREE.

No syntax errors found. CIF dictionary Interpreting this report

Datablock: e__131227d_work_cu_131227d_0m

```
Bond precision:  C-C = 0.0019 A          Wavelength=1.54178

Cell:            a=5.5649(1)     b=12.7775(3)       c=10.2937(2)
                 alpha=90        beta=93.934(1)     gamma=90
Temperature:     296 K

                Calculated                 Reported
Volume          730.21(3)                  730.21(3)
Space group     P 21                       P2(1)
Hall group      P 2yb                      ?
Moiety formula  C15 H16 O6, H2 O           ?
Sum formula     C15 H18 O7                 C15 H18 O7
Mr              310.29                     310.29
Dx,g cm-3       1.411                      1.411
Z               2                          2
Mu (mm-1)       0.955                      0.955
F000            328.0                      328.0
F000'           329.20
h,k,lmax        6,15,12                    6,15,12
Nref            2830[ 1481]                2700
Tmin,Tmax       0.892,0.909                0.894,0.910
Tmin'           0.892

Correction method= NONE

Data completeness= 1.82/0.95      Theta(max)= 71.550

R(reflections)= 0.0265( 2695)     wR2(reflections)= 0.0735( 2700)

S = 1.071                  Npar= 215
```

The following ALERTS were generated. Each ALERT has the format
test-name_ALERT_alert-type_alert-level.
Click on the hyperlinks for more details of the test.

Alert level B

```
PLAT035_ALERT_1_B No _chemical_absolute_configuration info given .     Please Do !
```

Alert level C

```
ABSTY03_ALERT_1_C  The _exptl_absorpt_correction_type has been given as none.
            However values have been given for Tmin and Tmax. Remove
            these if an absorption correction has not been applied.
  From the CIF: _exptl_absorpt_correction_T_min   0.894
  From the CIF: _exptl_absorpt_correction_T_max   0.910
PLAT029_ALERT_3_C _diffrn_measured_fraction_theta_full Low .......      0.968 Note
PLAT089_ALERT_3_C Poor Data / Parameter Ratio (Zmax < 18) ........       6.89 Note
```

Alert level G

```
PLAT002_ALERT_2_G Number of Distance or Angle Restraints on AtSite          7 Note
PLAT005_ALERT_5_G No _iucr_refine_instructions_details  in the CIF     Please Do !
PLAT066_ALERT_1_G Predicted and Reported Tmin&Tmax Range Identical          ? Check
PLAT720_ALERT_4_G Number of Unusual/Non-Standard Labels ..........          6 Note
PLAT791_ALERT_4_G The Model has Chirality at C2'     .............          S Verify
PLAT791_ALERT_4_G The Model has Chirality at C3'     .............          R Verify
PLAT860_ALERT_3_G Number of Least-Squares Restraints .............          5 Note
PLAT899_ALERT_4_G SHELXL97   is Deprecated and Succeeded by SHELXL       2014 Note
```

```
   0 ALERT level A = Most likely a serious problem - resolve or explain
   1 ALERT level B = A potentially serious problem, consider carefully
   3 ALERT level C = Check. Ensure it is not caused by an omission or oversight
   8 ALERT level G = General information/check it is not something unexpected

   3 ALERT type 1 CIF construction/syntax error, inconsistent or missing data
   1 ALERT type 2 Indicator that the structure model may be wrong or deficient
   3 ALERT type 3 Indicator that the structure quality may be low
   4 ALERT type 4 Improvement, methodology, query or suggestion
   1 ALERT type 5 Informative message, check
```

It is advisable to attempt to resolve as many as possible of the alerts in all categories. Often the minor alerts point to easily fixed oversights, errors and omissions in your CIF or refinement strategy, so attention to these fine details can be worthwhile. In order to resolve some of the more serious problems it may be necessary to carry out additional measurements or structure refinements. However, the purpose of your study may justify the reported deviations and the more serious of these should normally be commented upon in the discussion or experimental section of a paper or in the "special_details" fields of the CIF. checkCIF was carefully designed to identify outliers and unusual parameters, but every test has its limitations and alerts that are not important in a particular case may appear. Conversely, the absence of alerts does not guarantee there are no aspects of the results needing attention. It is up to the individual to critically assess their own results and, if necessary, seek expert advice.

Publication of your CIF in IUCr journals

A basic structural check has been run on your CIF. These basic checks will be run on all CIFs submitted for publication in IUCr journals (*Acta Crystallographica*, *Journal of Applied Crystallography*, *Journal of Synchrotron Radiation*); however, if you intend to submit to *Acta Crystallographica Section C* or *E*, you should make sure that full publication checks are run on the final version of your CIF prior to submission.

Publication of your CIF in other journals

Please refer to the *Notes for Authors* of the relevant journal for any special instructions relating to CIF submission.

PLATON version of 20/08/2014; check.def file version of 18/08/2014

Datablock e__131227d_work_cu_131227d_0m - ellipsoid plot

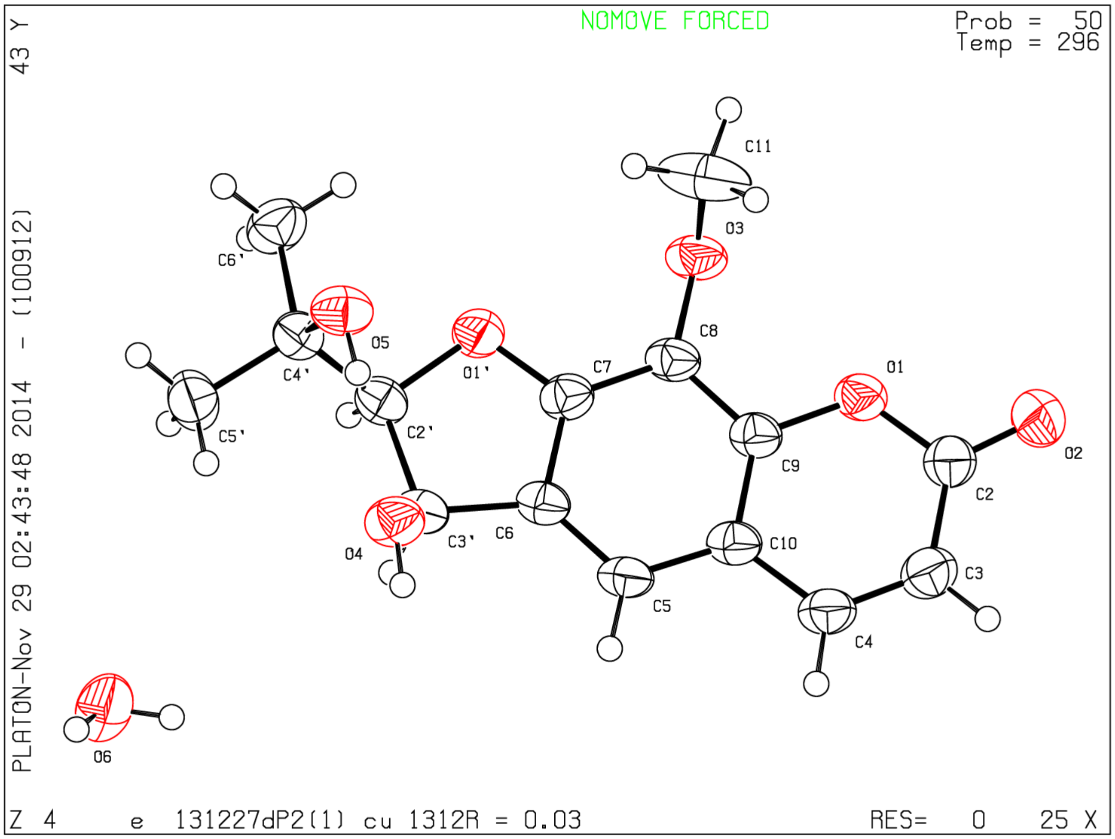

checkCIF/PLATON report

You have not supplied any structure factors. As a result the full set of tests cannot be run.

THIS REPORT IS FOR GUIDANCE ONLY. IF USED AS PART OF A REVIEW PROCEDURE FOR PUBLICATION, IT SHOULD NOT REPLACE THE EXPERTISE OF AN EXPERIENCED CRYSTALLOGRAPHIC REFEREE.

No syntax errors found.   CIF dictionary   Interpreting this report

Datablock: e__131205d_work_cu_131205d_0m

```
Bond precision:  C-C = 0.0019 A          Wavelength=1.54178

Cell:            a=5.7224(1)     b=13.8219(3)        c=7.8652(2)
                 alpha=90        beta=100.508(1)     gamma=90
Temperature:     298 K

                Calculated                 Reported
Volume          611.66(2)                  611.66(2)
Space group     P 21                       P2(1)
Hall group      P 2yb                      ?
Moiety formula  C14 H14 O4                 ?
Sum formula     C14 H14 O4                 C14 H14 O4
Mr              246.25                     246.25
Dx,g cm-3       1.337                      1.337
Z               2                          2
Mu (mm-1)       0.812                      0.812
F000            260.0                      260.0
F000'           260.87
h,k,lmax        7,16,9                     6,16,9
Nref            2361[ 1232]                2265
Tmin,Tmax       0.823,0.850                0.793,0.854
Tmin'           0.784

Correction method= NONE

Data completeness= 1.84/0.96      Theta(max)= 71.230

R(reflections)= 0.0290( 2258)     wR2(reflections)= 0.0809( 2265)

S = 1.051                   Npar= 167
```

The following ALERTS were generated. Each ALERT has the format
test-name_ALERT_alert-type_alert-level.
Click on the hyperlinks for more details of the test.

Alert level B

```
PLAT035_ALERT_1_B No _chemical_absolute_configuration info given .     Please Do !
```

Alert level C

```
ABSTY03_ALERT_1_C  The _exptl_absorpt_correction_type has been given as none.
            However values have been given for Tmin and Tmax. Remove
            these if an absorption correction has not been applied.
  From the CIF: _exptl_absorpt_correction_T_min   0.793
  From the CIF: _exptl_absorpt_correction_T_max   0.854
PLAT029_ALERT_3_C _diffrn_measured_fraction_theta_full Low .......      0.966 Note
PLAT089_ALERT_3_C Poor Data / Parameter Ratio (Zmax < 18) ........       7.38 Note
```

Alert level G

```
PLAT005_ALERT_5_G No _iucr_refine_instructions_details  in the CIF     Please Do !
PLAT007_ALERT_5_G Number of Unrefined Donor-H Atoms ..............          1 Report
PLAT093_ALERT_1_G No su's on H-positions, refinement reported as .      mixed
PLAT720_ALERT_4_G Number of Unusual/Non-Standard Labels ..........          8 Note
PLAT791_ALERT_4_G The Model has Chirality at C2'     .............          S Verify
PLAT899_ALERT_4_G SHELXL97   is Deprecated and Succeeded by SHELXL       2014 Note
```

```
   0 ALERT level A = Most likely a serious problem - resolve or explain
   1 ALERT level B = A potentially serious problem, consider carefully
   3 ALERT level C = Check. Ensure it is not caused by an omission or oversight
   6 ALERT level G = General information/check it is not something unexpected

   3 ALERT type 1 CIF construction/syntax error, inconsistent or missing data
   0 ALERT type 2 Indicator that the structure model may be wrong or deficient
   2 ALERT type 3 Indicator that the structure quality may be low
   3 ALERT type 4 Improvement, methodology, query or suggestion
   2 ALERT type 5 Informative message, check
```

It is advisable to attempt to resolve as many as possible of the alerts in all categories. Often the minor alerts point to easily fixed oversights, errors and omissions in your CIF or refinement strategy, so attention to these fine details can be worthwhile. In order to resolve some of the more serious problems it may be necessary to carry out additional measurements or structure refinements. However, the purpose of your study may justify the reported deviations and the more serious of these should normally be commented upon in the discussion or experimental section of a paper or in the "special_details" fields of the CIF. checkCIF was carefully designed to identify outliers and unusual parameters, but every test has its limitations and alerts that are not important in a particular case may appear. Conversely, the absence of alerts does not guarantee there are no aspects of the results needing attention. It is up to the individual to critically assess their own results and, if necessary, seek expert advice.

Publication of your CIF in IUCr journals

A basic structural check has been run on your CIF. These basic checks will be run on all CIFs submitted for publication in IUCr journals (*Acta Crystallographica*, *Journal of Applied Crystallography*, *Journal of Synchrotron Radiation*); however, if you intend to submit to *Acta Crystallographica Section C* or *E*, you should make sure that full publication checks are run on the final version of your CIF prior to submission.

Publication of your CIF in other journals

Please refer to the *Notes for Authors* of the relevant journal for any special instructions relating to CIF submission.

PLATON version of 20/08/2014; check.def file version of 18/08/2014

Datablock e__131205d_work_cu_131205d_0m - ellipsoid plot

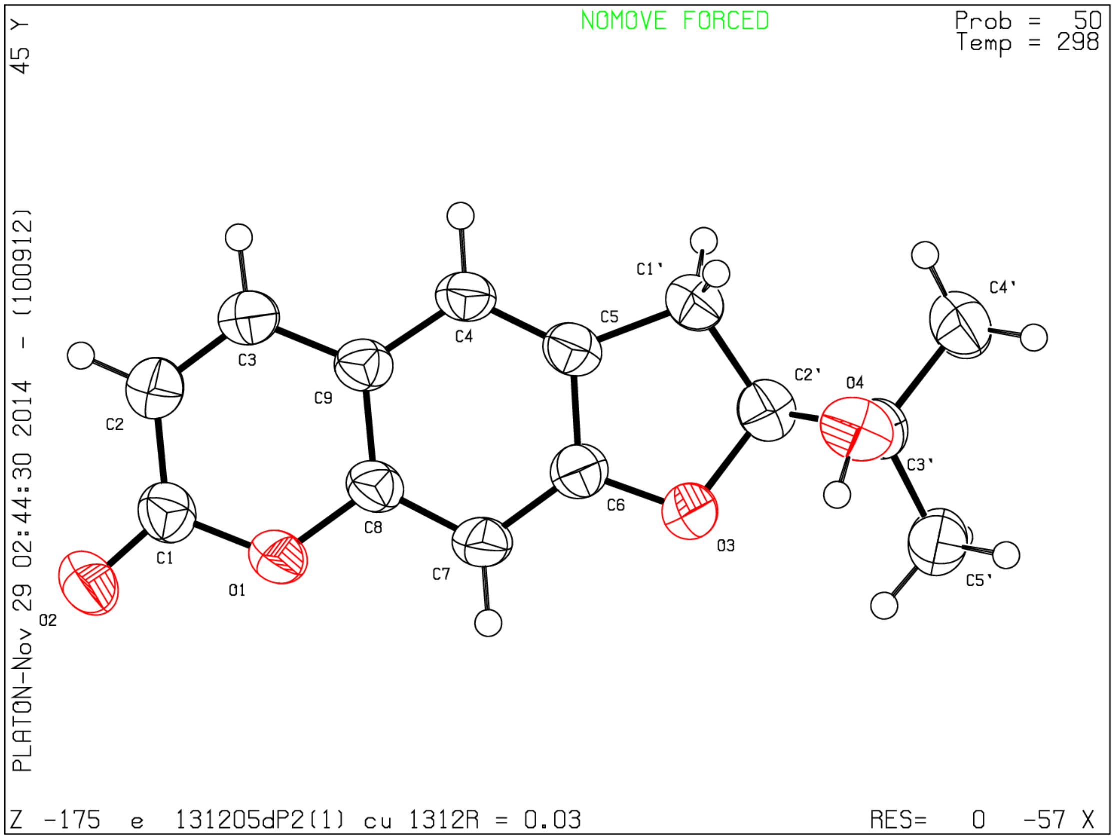

checkCIF/PLATON report

You have not supplied any structure factors. As a result the full set of tests cannot be run.

THIS REPORT IS FOR GUIDANCE ONLY. IF USED AS PART OF A REVIEW PROCEDURE FOR PUBLICATION, IT SHOULD NOT REPLACE THE EXPERTISE OF AN EXPERIENCED CRYSTALLOGRAPHIC REFEREE.

No syntax errors found.   CIF dictionary   Interpreting this report

Datablock: t

```
Bond precision:  C-C = 0.0029 A         Wavelength=0.71073

Cell:             a=12.883(2)      b=15.779(2)      c=4.8658(8)
                  alpha=90         beta=90          gamma=90
Temperature:      298 K

                Calculated                Reported
Volume          989.1(3)                  989.1(3)
Space group     P n a 21                  Pna2(1)
Hall group      P 2c -2n                  ?
Moiety formula  C12 H8 O4                 ?
Sum formula     C12 H8 O4                 C12 H8 O4
Mr              216.18                    216.18
Dx,g cm-3       1.452                     1.452
Z               4                         4
Mu (mm-1)       0.110                     0.110
F000            448.0                     448.0
F000'           448.28
h,k,lmax        18,22,6                   18,22,6
Nref            2880[ 1598]               2863
Tmin,Tmax       0.984,0.989               0.984,0.989
Tmin'           0.984

Correction method= NONE

Data completeness= 1.79/0.99      Theta(max)= 29.980

R(reflections)= 0.0486( 2074)     wR2(reflections)= 0.1467( 2863)

S = 1.007                  Npar= 146
```

The following ALERTS were generated. Each ALERT has the format
test-name_ALERT_alert-type_alert-level.
Click on the hyperlinks for more details of the test.

Alert level C

```
ABSTY03_ALERT_1_C  The _exptl_absorpt_correction_type has been given as none.
            However values have been given for Tmin and Tmax. Remove
            these if an absorption correction has not been applied.
  From the CIF: _exptl_absorpt_correction_T_min   0.984
  From the CIF: _exptl_absorpt_correction_T_max   0.989
STRVA01_ALERT_4_C           Flack parameter is too small
           From the CIF: _refine_ls_abs_structure_Flack   -0.500
           From the CIF: _refine_ls_abs_structure_Flack_su    1.400
PLAT230_ALERT_2_C Hirshfeld Test Diff for    C1'    --  C2'     ..        5.8 su
```

Alert level G

```
PLAT005_ALERT_5_G No _iucr_refine_instructions_details  in the CIF     Please Do !
PLAT032_ALERT_4_G Std. Uncertainty on Flack Parameter Value High .      1.400 Report
PLAT066_ALERT_1_G Predicted and Reported Tmin&Tmax Range Identical          ? Check
PLAT093_ALERT_1_G No su's on H-positions, refinement reported as .      mixed
PLAT899_ALERT_4_G SHELXL97   is Deprecated and Succeeded by SHELXL       2014 Note
```

```
   0 ALERT level A = Most likely a serious problem - resolve or explain
   0 ALERT level B = A potentially serious problem, consider carefully
   3 ALERT level C = Check. Ensure it is not caused by an omission or oversight
   5 ALERT level G = General information/check it is not something unexpected

   3 ALERT type 1 CIF construction/syntax error, inconsistent or missing data
   1 ALERT type 2 Indicator that the structure model may be wrong or deficient
   0 ALERT type 3 Indicator that the structure quality may be low
   3 ALERT type 4 Improvement, methodology, query or suggestion
   1 ALERT type 5 Informative message, check
```

It is advisable to attempt to resolve as many as possible of the alerts in all categories. Often the minor alerts point to easily fixed oversights, errors and omissions in your CIF or refinement strategy, so attention to these fine details can be worthwhile. In order to resolve some of the more serious problems it may be necessary to carry out additional measurements or structure refinements. However, the purpose of your study may justify the reported deviations and the more serious of these should normally be commented upon in the discussion or experimental section of a paper or in the "special_details" fields of the CIF. checkCIF was carefully designed to identify outliers and unusual parameters, but every test has its limitations and alerts that are not important in a particular case may appear. Conversely, the absence of alerts does not guarantee there are no aspects of the results needing attention. It is up to the individual to critically assess their own results and, if necessary, seek expert advice.

Publication of your CIF in IUCr journals

A basic structural check has been run on your CIF. These basic checks will be run on all CIFs submitted for publication in IUCr journals (*Acta Crystallographica*, *Journal of Applied Crystallography*, *Journal of Synchrotron Radiation*); however, if you intend to submit to *Acta Crystallographica Section C* or *E*, you should make sure that full publication checks are run on the final version of your CIF prior to submission.

Publication of your CIF in other journals

Please refer to the *Notes for Authors* of the relevant journal for any special instructions relating to CIF submission.

PLATON version of 20/08/2014; check.def file version of 18/08/2014

Datablock t - ellipsoid plot

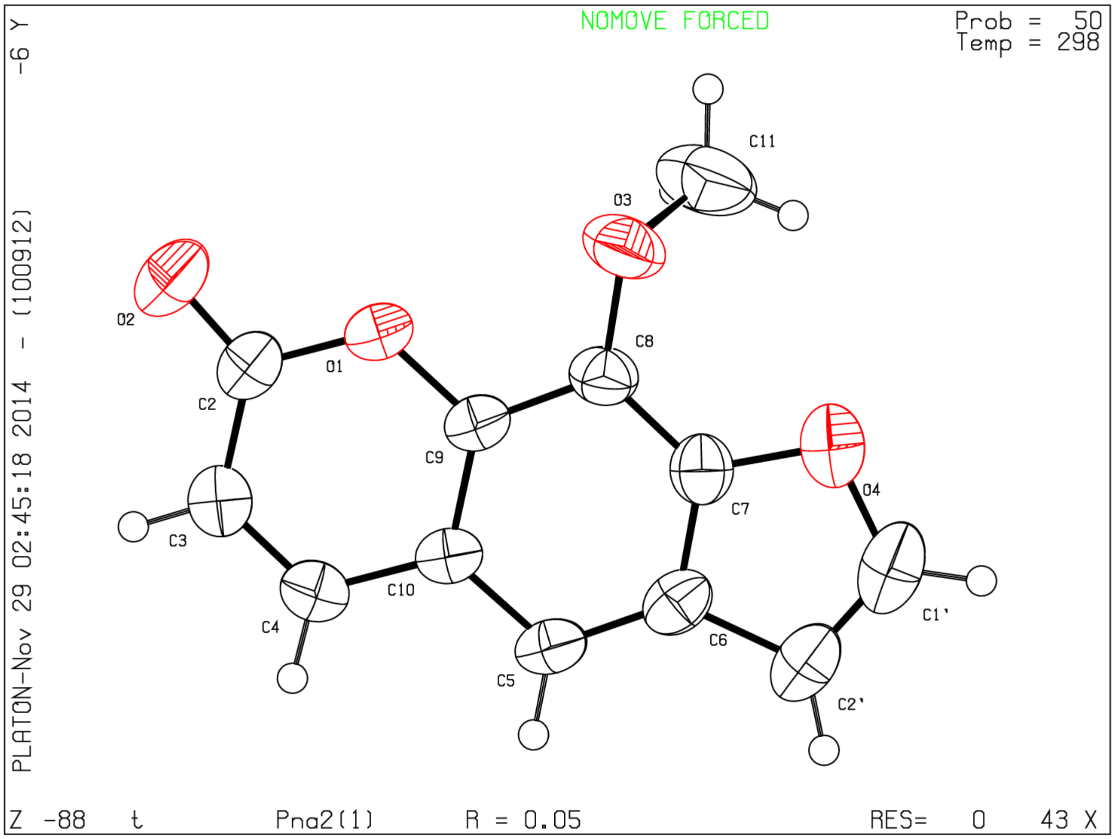

Supplement: Supporting Information [file srep13544-s1.pdf]
